# Fe-Doped Ni-Based Catalysts Surpass Ir-Baselines for Oxygen Evolution Due to Optimal Charge-Transfer Characteristics

**DOI:** 10.1021/acscatal.4c04489

**Published:** 2024-11-11

**Authors:** Mai-Anh Ha, Shaun M. Alia, Andrew G. Norman, Elisa M. Miller

**Affiliations:** † Computational Science Center, National Renewable Energy Laboratory, 15013 Denver West Parkway, Golden, Colorado 80401, United States; ‡ Chemistry and Nanoscience Center, National Renewable Energy Laboratory, 15013 Denver West Parkway, Golden, Colorado 80401, United States; § Materials Science Center, National Renewable Energy Laboratory, 15013 Denver West Parkway, Golden, Colorado 80401, United States

**Keywords:** doped-metal catalysts, electrolysis, oxygen evolution reaction, earth-abundant materials, non-platinum group metals, computational chemistry, mechanistic study of reactions

## Abstract

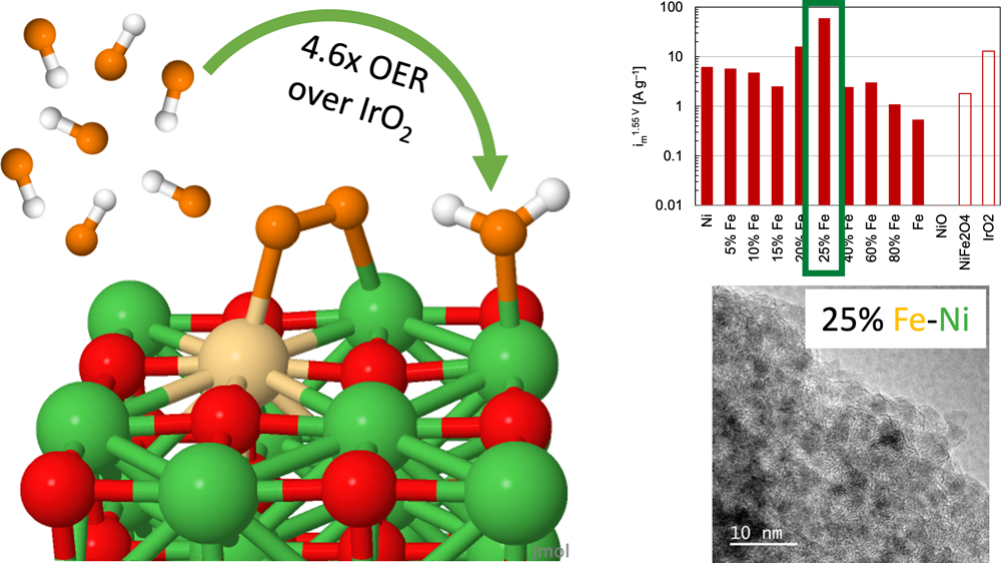

Ni-based catalysts with Co or Fe can potentially replace precious Ir-based catalysts for the rate-limiting oxygen evolution reaction (OER) in anion-exchange membrane (AEM) electrolyzers. In this study, density functional theory (DFT) calculations provide atomic- and electronic-level resolution on how the inclusion of Co or Fe can overcome the inactivity of NiO catalysts and even enable them to surpass IrO_2_ in activating key steps to the OER. Namely, NiO resists binding the key OH* intermediate and presents a high energetic barrier to forming the O*. Co- and Fe-substitution of Ni active sites allows for the stronger binding of OH* and preferentially activates O*/O_2_* formation, with Fe-substitution increasing the OER activity substantially as compared to Co-substitution. Whereas IrO_2_ requires an activation energy of 0.34–0.49 eV to form O_2_, this step is spontaneous on Fe_sub_-NiO. Electrodeposition of polycrystalline electrodes and synthesized nanoparticles exploit the Co or Fe presence, with Fe particularly exhibiting greater activity: Tafel slopes indicate a significant change in the mechanism as compared to pure NiO, validating the theoretical predictions of OER activation at different steps. High-performing synthesized nanoparticles of 25% Fe–Ni exhibited a 4.6 times improvement over IrO_2_ and a 34% improvement over RuO_2_, showcasing that non-platinum group metal catalysts can outperform platinum group metals. High-resolution transmission electron microscopy further highlights the advantages of Fe–Ni oxide synthesized nanoparticles over commercial catalysts: small, randomly oriented nanoparticles expose greater edge sites than large nanoparticles typical of commercially available materials.

## Introduction

Worldwide, multiple initiatives are in place to reduce carbon emissions and increase the clean production of hydrogen: the United States’ Department of Energy Hydrogen Shot aims for $1 per 1 kg of hydrogen in 1 decade;^1,2^ the European REPowerEU Plan projects an increase of 10 million tonnes of domestic renewable hydrogen production by 2030; similarly, Japan’s Green Growth Strategy plans to reduce hydrogen cost to less than one-third within the same time period.^3,4^ Electrolysis, the electrochemical splitting of water, remains a key aspect of these initiatives, requiring relatively ambient operating conditions and coupling to intermittent, renewable energy sources such as solar and wind to further lower hydrogen production costs.^1,2^ Of particular interest is anion-exchange membrane (AEM) electrolysis, which allows for the use and development of cheap, earth-abundant metals for catalysts and other electrode components, reducing stack costs considerably: our Fe-doped NiO catalysts exhibit activity comparable to that of commercial Ir-based catalysts, with higher performance at moderate current densities.

For electrolysis to be cost-competitive with steam methane reforming, a high-temperature process utilizing fossil fuels to cheaply produce hydrogen (H_2_) at <$2/kg, electrolyzer stacks must reduce capital costs, and much of this cost reduction will occur through advanced manufacturing. The use of platinum group metal (PGM)-free catalysts in the oxygen evolution reaction (OER) with improved electrolyzer performance and stability, however, is critical to the cost and value-added proposition of alkaline systems.^5^ Currently, OER requires the highest catalyst loading and thus significantly drives net catalyst cost: this cost is magnified in proton exchange membrane electrolysis, where Ir-based catalysts are utilized, and partially mitigated in AEM electrolysis, where earth-abundant materials can be used but often require much higher overpotentials than Ir.^6,7^

Recent experimental studies suggest that Ni-based catalysts in basic media may be the strongest alternative to Ir-based catalysts when optimized with Fe or Co.^6,8−12^ However, the efficiency of these catalysts is strongly synthesis-dependent. Notably, solution-cast metal oxides^8^ resulted in catalyst efficiency on the order of Ni_0.9_Fe_0.1_O_*x*_ > NiO_*x*_ > Ni_*y*_Co_1–*y*_O, whereas electrodeposited thin films^9^ classified activity into these three categories of FeNiO_*x*_, CoFeO_*x*_, CoFeNiO_*x*_ > CoO_*x*_, CoNiO_*x*_ > FeO_*x*_, NiO_*x*_. In benchmarking studies of commercial materials, McCrory et al.^6^ reported OER overpotentials increased from IrO_*x*_ < NiFeO_*x*_ < NiCoO_*x*_ < CoO_*x*_ < NiO_*x*_; Anderson et al.^7^ observed activities on the order of Ir > IrO_2_ > Co > Ni > NiFe_2_O_4_ ≫ NiO; and Volk et al.^11^ noted that Ni- and Co-based commercial catalysts often met or outperformed IrO_2_ in both rotating disk electrode (RDE) and membrane electrode assemblies (MEAs). Furthermore, Volk et al. found that over time-on-stream of benchmark tests, NiFe_2_O_4_’s activity increased with increasing ratios of Ni:Fe due to Fe dissolution, suggesting that similar to solution-cast metal oxides, where Ni_0.9_Fe_0.1_O_*x*_ was the high performer, a higher percentage of Ni:Fe in these mixed-metal oxide catalysts may be preferable.

Much of our mechanistic understanding of OER activity has been concentrated on reaction pathways in an acidic environment, e.g., the four-step, proton-coupled electron transfer mechanism, whereas the alkaline environment of AEM electrolysis will necessarily induce other electrochemical processes:^13−15^
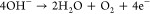
1

We note that multiple pathways at each step of the OER mechanism may be possible in the high potential environment of electrolysis.^13,16,17^ Furthermore, electronic structure calculations can more accurately reflect a material’s properties through statistical mechanical arguments detailing the ensemble effects of adsorbates on reactivity and through coadsorption of key intermediates, which can highlight low-energy pathways.^16,18,19^ Understanding how and why these catalysts work and do not work is critical to enabling these materials to achieve commercial viability: our fundamental understanding of OER mechanisms based on the chemical interactions possible in an alkaline environment coupled with the experimental synthesis and characterization of NiO compared to Fe- and Co-NiO catalysts can significantly inform and advance AEM electrolysis.

(Oxy)hydroxide catalysts have been studied extensively, but the stable NiO catalyst has been minimally studied.^9,13,14,20−22^ He et al. noted that the spin state of Fe is key to the high reactivity of NiOOH-based catalysts, whereas Martirez et al. cited the presence of an Fe^4+^=O species for lowering the overpotential for OER.^13,22^ These trends may apply similarly to other Ni-based catalysts such as rock-salt NiO. Synthesis procedures often feature thermal annealing in reducing or oxidizing environments, acid- or base-leaching treatments, and electrochemical cycling in order to optimize the activity and durability of catalysts.^23,24^ Ni-based catalysts can cycle through the α-, β-, γ-(oxy)hydroxides,^21,24^ and NiO may form depending on applied potentials, thermal treatments, and starting materials (metal, hydroxide, or oxide).^25−27^ Following holds at ca. 200 °C, powdered hydroxides transitioned into a mixture of NiO and Ni-(oxy)hydroxides, and beyond 200 °C, the NiO became dominant.^25^ Likewise, the oxidation of Ni (111) surfaces at 300 K (26.85 °C) followed by Langmuirs of water resulted in a transition of Ni (111) to NiO (111) to β-Ni(OH)_2_, whereas oxidation at 500 K (226.85 °C) followed by similar water treatments of Ni (111) primarily formed NiO (100).^27^

Interest in NiO remains high since it can potentially be bifunctional for the hydrogen evolution revolution^28,29^ and, beyond electrolysis, has been utilized for the selective epoxidation of styrene to styrene oxide (a key component for the production of fine chemicals and pharmaceuticals);^30^ as a reactive gas sensor for formaldehyde, methane, and acetone;^31^ and in electrochromic uses as coatings for modulating daylight in windows or reflective transmittance in rear-view mirrors.^32^ Therefore, a greater understanding of the binding motifs and reactivity present in NiO versus Fe- and Co-modified NiO may have far-reaching implications across a diverse range of applications beyond electrolysis and energy.

Recently, Sun et al. attributed a 200-fold enhancement in OER activity to edge sites along the NiO (100) nanofacet, suggesting that greater attention to this facet should be considered.^14^ In this work, we extensively outline the mechanisms for achieving the OER on NiO (100) in an alkaline environment, examine the pathways limiting NiO activity, and compare this to IrO_2_ (110), Co-NiO (100), and Fe-NiO (100). The rock-salt NiO resists the charge transfer necessary to favor OH* adsorption and O* formation, whereas Co- and Fe-substitution supplies the charge for these initial steps to OER. Site access and activity in theoretical calculations were then correlated to various catalysts: commercial particles were examined, electrodeposition on metal electrodes was performed for controlled comparisons of site access, and synthesized nanoparticles highlighted the size and edge effects.

## Results and Discussion

### I Understanding the Viability of Rock-Salt NiO-Based Materials for the OER

**Figure 1:**
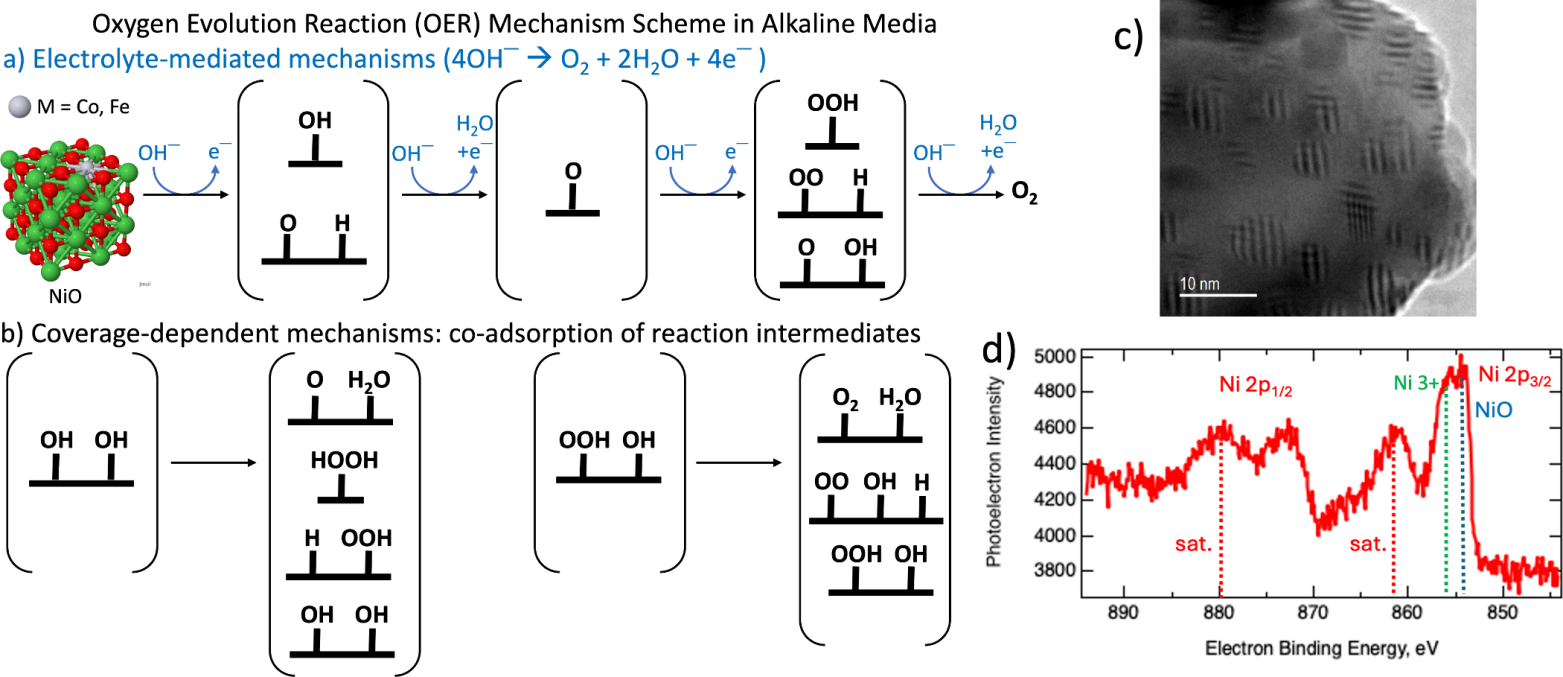
(a) The electrolyte-mediated mechanism is a four-step OER mechanism that relies on a hydroxide ion interacting with adsorbates. (b) The coverage-dependent mechanism utilizes the interaction between neighboring coadsorbed species to produce OER intermediates. Brackets [] signify that there can be multiple products, resulting in different, accessible pathways depending on the experiment (thermal conditions and electrochemical potentials). (c) HRTEM of commercial NiO catalysts. (d) XPS data of commercial NiO catalysts.

Our theoretical model examined one metal dopant at various sites in order to focus on the impact of Co and Fe in NiO (100): adsorbed to the surface as a monomer, substituting a Ni site, and embedded as an interstitial defect (Supporting Information, SI Tables 1–3). The interstitial site relaxed to a Co or Fe rising to the surface to displace a Ni, resulting in a Co- or Fe-substituted surface (see SI Figure 1a). In contrast, the adsorbed dopant on a surface oxygen may behave similarly to a monomer, where it can exhibit high reactivity but also be unstable since monomers are known to be mobile and sinter into larger clusters.^33,34^ Upon adsorption of an OH*, the enthalpy of the adsorbed dopant to shift from being atop a surface oxygen to a bridging Ni–Ni site is 0.00 eV for Fe_ads_ and 0.01 eV for Co_ads_ (see SI Figure 1b). These results suggest that both the interstitial and adsorbed sites are unstable, and subsequently, OH* adsorption was stronger and ranged between −4 and −5 eV. In contrast, the substituted dopant remained in the same site, even with OH* adsorption, highlighting that it may be more stable in the alkaline environment of AEM electrolysis. Therefore, Co_sub_-NiO and Fe_sub_-NiO were the focus of more detailed OER calculations (Figure 1a,b for the surface and mechanisms). These different doped-NiO surfaces represent an active metal site percentage of 11–13% for the dopant, with 87–89% of Ni available for O_*x*_H_*y*_ intermediates to adsorb onto. How our model compares to the various catalysts (commercial particles, galvanically displaced electrodes, and synthesized nanoparticles) examined in this paper will be discussed.

In addition to theoretical calculations, further characterization of available commercial NiFe_2_O_4_, Ni-, and Ir-based catalysts was performed. In high-resolution transmission electron microscopy (HRTEM) images of commercial NiO catalysts, we did detect the presence of defects or precipitates regions showing moiré fringes, which could contribute to the reactivity of this material (Figure 1c). Transmission electron diffraction (TED) suggests a crystalline catalyst (SI Figure 2) with lattice spacings consistent with rock-salt NiO; this phase was further confirmed by XRD. Energy-dispersive X-ray spectroscopy (EDS) found, as expected, that both Ni and O were present (SI Figure 3). The small Cu EDS peak is from the Cu TEM grid that was used. X-ray photoelectron spectroscopy (XPS) data of commercial Ni (SI Figures 4, 8, 9) and NiO_*x*_ (Figure 1d; SI Figures 8 and 9) catalysts detected the presence of NiO and Ni^3+^ on the surface. The NiO peak position of 854.12 eV for commercial NiO matches closely with the 853.7 eV by Biesinger et al., with some difference due to the different surface oxidation.^35^ Detailed discussion and XPS figures may be found in the SI. While the commercial Ni metal sample contained Ni^0^, we did not detect Ni^0^ in the commercial NiO sample. Similarly, XPS spectra revealed Ir metal and IrO_2_ in commercial Ir and only IrO_2_ in the commercial IrO_*x*_ (SI Figures 5–7). The IrO_2_ peak positions at 61.76 for Ir and 62.13 for IrO_*x*_ in these commercial materials complemented the 61.9–62.5 eV range specified by Freakley et al. (SI Figure 7).^36^ Although commercial catalysts are often promoted as a “metal” or “metal oxide catalyst,” all of the “metal” catalysts examined in this study through XPS contained metal oxides. In half-cell and single-cell electrochemical benchmarks of commercial catalysts, experiment observed that “Ir metal” catalysts exhibited the same activity as “Ir metal oxide” catalysts after 13.5 h.^37^ Due to the high potentials and the long operation times of commercial electrolysis (5–10 years), the catalysts are expected to be metal oxides instead of metals.^2,5^ For this study, the theoretical calculations focused on metal oxides under the following alkaline conditions: IrO_2_ and rock-salt NiO, CoO, FeO, and M_sub_-NiO.

In AEM electrolysis, O_2_ evolution requires the adsorption and interaction of a total of 4OH^—^ to form the different O-containing intermediates leading to O_2_ evolution. Figure 1a,b illustrates the OER pathways available: in Figure 1a, OH may interact with the interface as an OH^—^ (anion) in an “electrolyte-mediated mechanism” (Section I.a), or in Figure 1b, OH may coadsorb with other O-containing intermediates in a “coverage-dependent mechanism” to form the different OER products (Section I.b). In our previous work on IrO_2_ (110), we found that “coverage-dependent” mechanisms were able to showcase multiple, low-energy pathways to form OER products and potentially reflect more accurately the catalytic properties of a material.^16^ Various OER products are accessible at room temperature or at the high operating potentials of electrolysis (up to ca. 2.0 V).

#### I.a Oxygen Evolution at Low O_*x*_H_*y*_ Coverage, Reliant upon an “Electrolyte-Mediated Mechanism”

**Figure 2:**
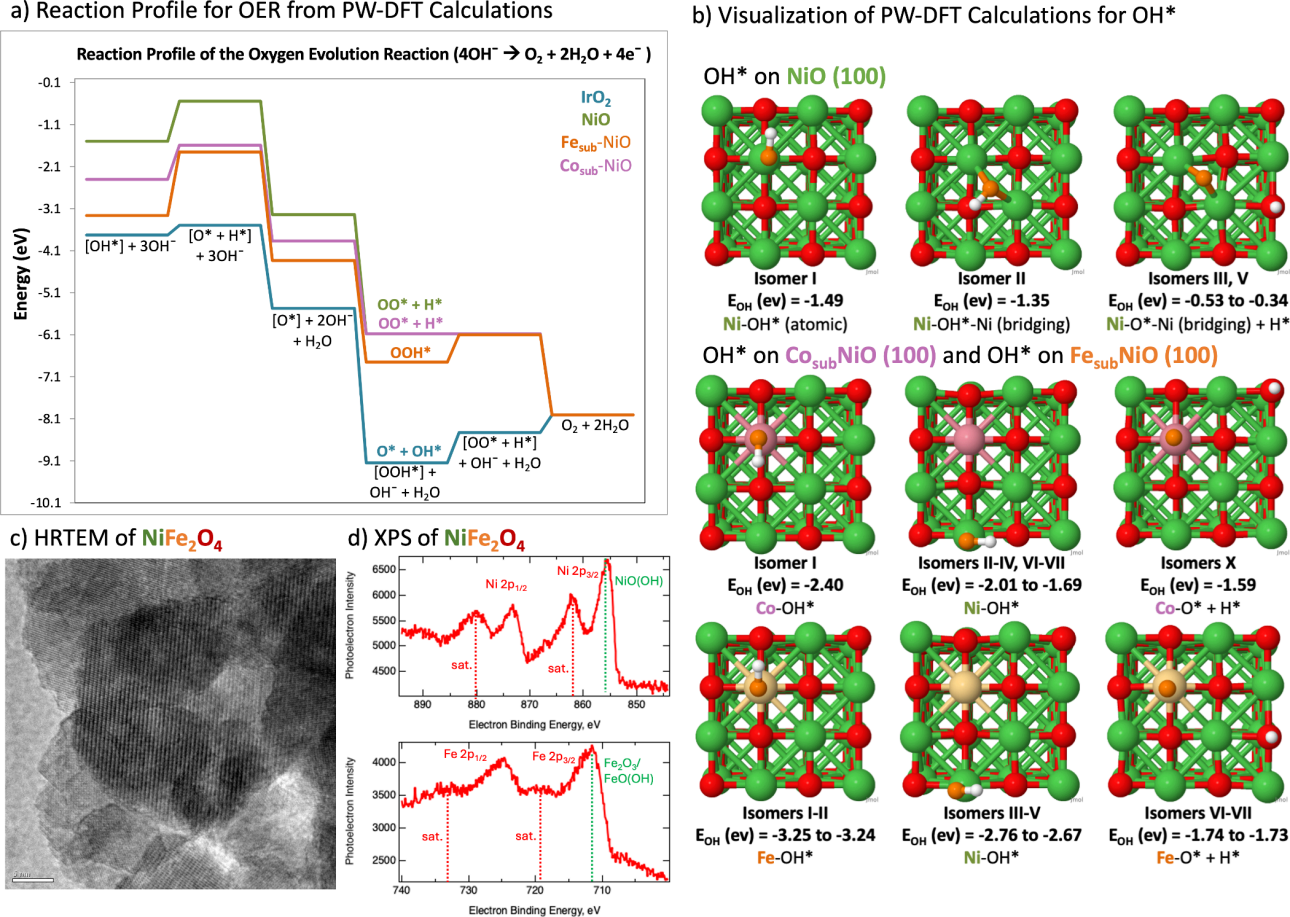
(a) Reaction profile outlining the OER. Only the global minimum (the lowest energy isomer) was utilized for this reaction profile. (b) Summary of OH* isomers with adsorption energy (*E*_OH_), where Ni atoms are shown in green, surface O in red, adsorbate O in orange, H in white, Co in pink, and Fe in yellow. Visualization of individual isomers, Bader charges, and Boltzmann populations may be found in the Supporting Information. (c) HRTEM of commercial NiFe_2_O_4_ showcasing the large grains present (5 nm bar). (d) XPS detected multiple distinct phases in commercial NiFe_2_O_4_ of Ni^3+^/NiO(OH) and Fe_2_O_3_/FeO(OH).

In Figure 2a, we highlight the reaction profile based on the global minimum (the most stable and lowest energy isomer) structures in the electrolyte-mediated mechanism. We point out that this mechanism and reaction profile rely upon a low coverage of adsorbates, wherein a key reaction intermediate is adsorbed on a single metal active site on the surface. This gives an overview of the possible differences in activity displayed by the PGM-baseline material IrO_2_ as compared to the non-PGM materials such as NiO and Co_sub_-, Fe_sub_-NiO. A free energy reaction profile is also provided in SI Figure 10, but the trends displayed in Figure 2a remain the same. Different theoretical studies may utilize other programs and approximations (thermodynamic versus applied potential, implicit versus explicit solvation) in order to calculate free energies.^15,38,39^ Total and relative energies may be directly compared regardless of the program utilized: this study focuses on the mechanistic pathways available to doped-NiO catalysts as compared to benchmark NiO and IrO_2_, and the effect of the complex, electrochemical environment will be delved into in future work.

We summarize and highlight the first key step to OER, the adsorption of OH* followed by the formation of O* + H* in Figure 2b. As noted in eq 1, 4OH^–^ are required to form H_2_O and O_2_. Without enough OH* adsorbed to the surface to form these two products, the OER will be halted. We found that for O*, the hollow site is the most stable at NiO, but for the Co- and Fe-doped NiO surfaces, the metal dopant site is the most stable. In particular, OOH* is not the most stable isomer on NiO (100), Co_sub_-NiO (100), or IrO_2_ (110), and at low potentials, the mechanism of these coadsorbed intermediates of O* + OH* or OO* + H* will dominate. At higher potentials, the less stable OOH* isomer becomes accessible, providing an additional pathway to forming O_2_. NiO and Co_sub_-NiO may be particularly advantageous to deprotonating the OOH* since this intermediate spontaneously dissociates on these surfaces, whereas Fe_sub_-NiO stabilizes the OOH* (isomers I–III, V–VI), and the dissociated OO* + H* products (isomers X, XII–XX) are ca. 0.6–0.9 eV higher in energy. SI Figures 12–14, 16–19 display the considerable number of isomers available to NiO, Co_sub_-NiO, and Fe_sub_-NiO for the key reaction intermediates of [OH*], [O*], and [OOH*].

Bronoel et al. established in their electrochemical studies of NiO that O_2_ evolution most likely utilized the mechanism of OH* → O*, followed by the recombination of 2O* → O_2_.^40^ Most importantly, Figure 2a,b showcases that NiO (100) may adsorb the OH* reactant too weakly to form O_2_ and 2H_2_O as compared to IrO_2_ (eq 1). Indeed, while metal oxides are often known to hydroxylate in the presence of water vapor, Cappus et al. found only NiO (111) adsorbed OH, whereas NiO (100) was unable to adsorb OH until defects were created on the surface.^41^ This resistance to adsorbing OH* may be attributed to (100) NiO’s charge-transfer capability: this nonpolar surface features balanced electrostatic interactions between the metal and oxygen, and this immense stability leads to less reactivity as compared to the polar (111) surface with its dangling bonds.

Calculations of pure NiO, CoO, and FeO in both the bulk and the (100) surface yielded Bader charges (ΔQ) of circa +1.2 to +1.3 for metal atoms and −1.2 to −1.3 for oxygen atoms (SI Tables 1 and 2). This charge-transfer difference between metal and oxygen atoms remains similar to the Co_sub_-NiO and Fe_sub_-NiO surfaces. It is upon adsorption of key OER intermediates, however, that the Bader charges of the dopant metal atom change significantly. The dopants, Co and Fe, essentially become electron donors to all of the OER intermediates, whether they adsorb to the dopant site or the Ni site. This effect leads to the greatest increase in the OH* binding energy, specifically, on the Co and Fe dopant sites: ca. 1 eV increase on Co_sub_-NiO and ca. 2 eV increase on Fe_sub_-NiO (Table 1). Indeed, even Ni sites on the Co_sub_-NiO and Fe_sub_-NiO surfaces bind OH* more strongly than those on the pure NiO surface. All other OER intermediates follow a similar but less pronounced trend. The Fe dopant offers the most significant charge transfer at +1.6 to +1.7 e (comparable to Ir sites, Table 1), resulting in stronger binding of the OER intermediates. These results suggest that transition metal dopants can potentially allow non-PGM catalysts to mimic the binding motifs present in a PGM catalyst and, most importantly, increase the reactivity of the non-PGM catalyst: the OH* binding strength on Fe_sub_-NiO is nearly comparable to that of IrO_2_ (110).

**Table 1:** Summary of OH* Isomers of NiO, Co_sub_-NiO, and Fe_sub_-NiO with Adsorption Energy (E_OH_) and Bader Charges (ΔQ)

	IrO_2_	NiO	Co_sub_-NiO		Fe_sub_-NiO	
	I–III	I–II (Ni site)	I (Co site)	II–IX (Ni site)	I–II (Fe site)	III–V (Ni site)
*E*_OH_ (ev)	–3.72 to −3.66	–1.37 to −1.27	–2.40	–2.01 to −1.19	–3.25 to −3.24	–2.76 to −2.67
Δ*Q*_OH_ (e)	–0.41 to −0.48	–0.43 to −0.50	–0.46	–0.63 to −0.75	–0.55	–0.68 to −0.73
Δ*Q*_M_ (e)	+1.69 to +1.71		+1.49	+1.39 to +1.56	+1.65	+1.64 to +1.69
Δ*Q*_Ni_ (e)		+1.20 to +1.29		+1.19 to +1.29		+1.18 to +1.29

Fe’s advantageous charge-transfer capability may contribute to the high activity observed in mixed Ni–Fe oxides beyond the rock-salt phase. Depending on the synthesis methods of Fe–Ni oxide catalysts, the mole % of Fe can significantly influence the crystalline forms: mixed NiO/NiFe_2_O_4_ (≤20 mol % Fe) or NiO/NiFe_2_O_4_/Fe_2_O_3_ (≥25 mol % Fe) material.^12^ This showcases the importance of deconvoluting the contributions of specific crystalline phases to the catalytic activity. Our XPS of commercial NiFe_2_O_4_ found Ni to be N^3+^/NiO(OH), and not NiO, while the Fe was found to be Fe^3+^/Fe_2_O_3_/FeO(OH) (Figure 2d). XPS spectra suggest a surface ratio of Ni:Fe 1.94:1. EDS in TEM found elemental ratios close to those expected for NiFe_2_O_4_ (SI Figure 15). HRTEM (see Figure 2b) found that the commercial NiFe_2_O_4_ catalysts are highly crystalline and composed of 10–15 nm aggregates, and lattice spacings calculated from the TED pattern indicate spinel NiFe_2_O_4_ (SI Figure 15). Volk et al. observed a similar inhomogeneity to different samples of commercial NiFe_2_O_4_, where XRD found both the spinel and α-Fe_2_O_3_.^11^ Lower Fe content (<25%), similar to the theoretical model, was often associated with greater activity and the presence of NiO.^6,8−12^

However, these non-PGM catalysts are not as adept at spontaneously splitting OH* into O* + H*: on IrO_2_ (110) the dissociated O* + H* isomer IV is only ca. 0.23 eV higher in energy than the intact OH*. The Ir metal exhibits a greater charge transfer of +1.9 e to the dissociated O* + H* isomer, suggesting that an additional 0.2 e may be required to aid in this step (SI Figure 11). In contrast, on NiO, the dissociated O* + H* is 0.96 eV higher in energy; on Co_sub_-NiO, 0.89 eV for isomer X; and on Fe_sub_-NiO, 1.51 eV for isomer VI. In fact, these materials resist charge transfer with metal sites at circa +1.2 to +1.4 e for the dissociated O* + H* isomer (SI Figures 12−14).

#### I.b Oxygen Evolution at High O_*x*_H_*y*_ Coverage for the “Coverage-Dependent Mechanism”

**Figure 3:**
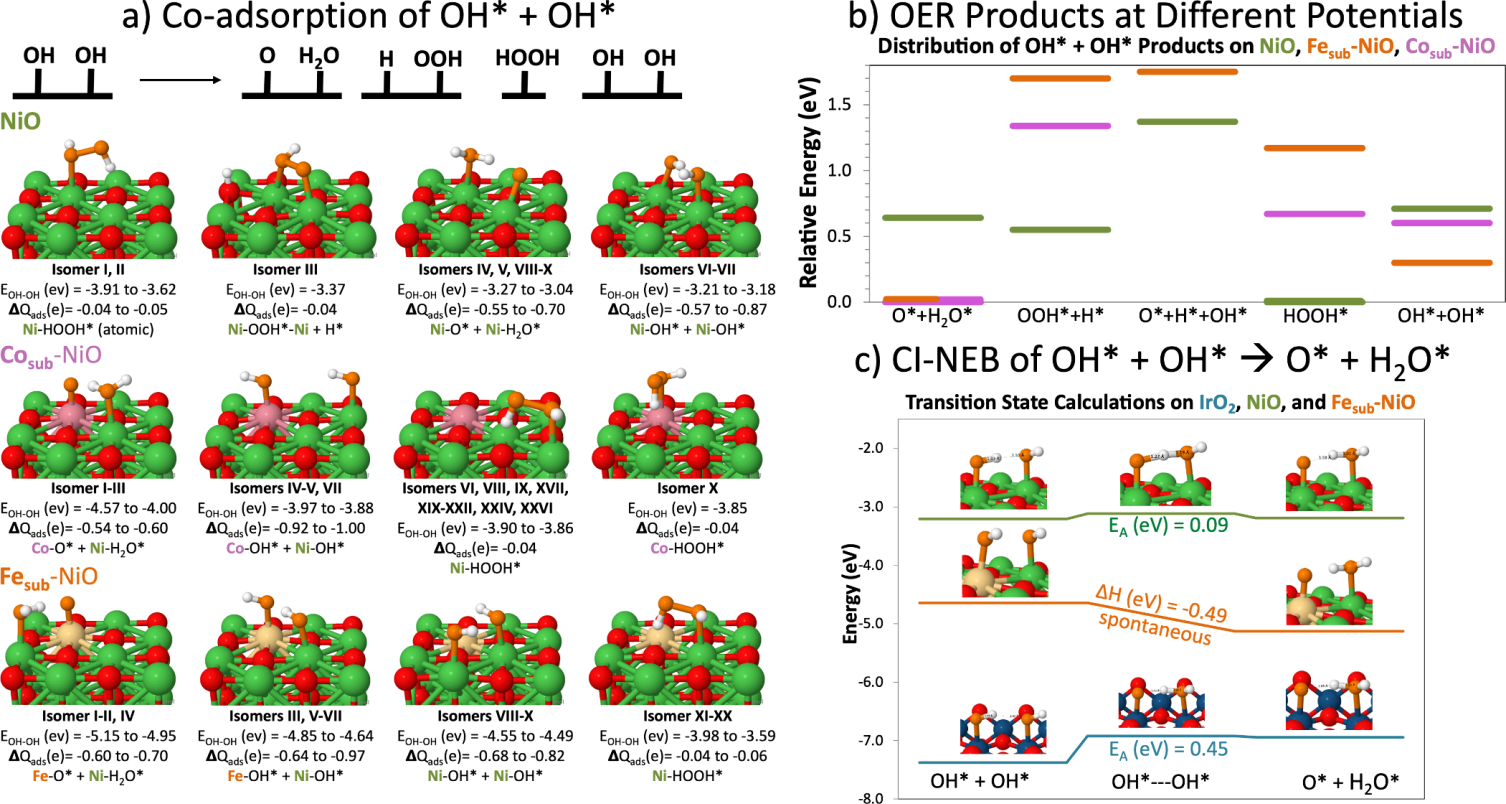
O* Formation: (a) OH* + OH* → O* + H_2_O*, H* + OOH*, HOOH*, or unreacted OH*+ OH* isomers with adsorption energy (*E*_OH+OH_) and Bader charges (Δ*Q*). All isomers are illustrated in detail in the SI. Ni atoms are in green; surface O—red; adsorbate O—orange; H—white; Co—pink; and Fe—yellow. (b) Plot of isomers within 2.0 V of electrolysis. For Co_sub_- and Fe_sub_-NiO, the global minimum is O* + H_2_O*, marked by orange and pink. Green reflects the isomers of NiO; pink is for Co_sub_-NiO; and orange is for Fe_sub_-NiO. (c) Climbing image nudged-elastic band calculations for OH* + OH* → O* + H_2_O*, showcasing transition state barriers for NiO and IrO_2_ as compared to the spontaneous, exothermic reaction on Fe_sub_-NiO.

**Figure 4:**
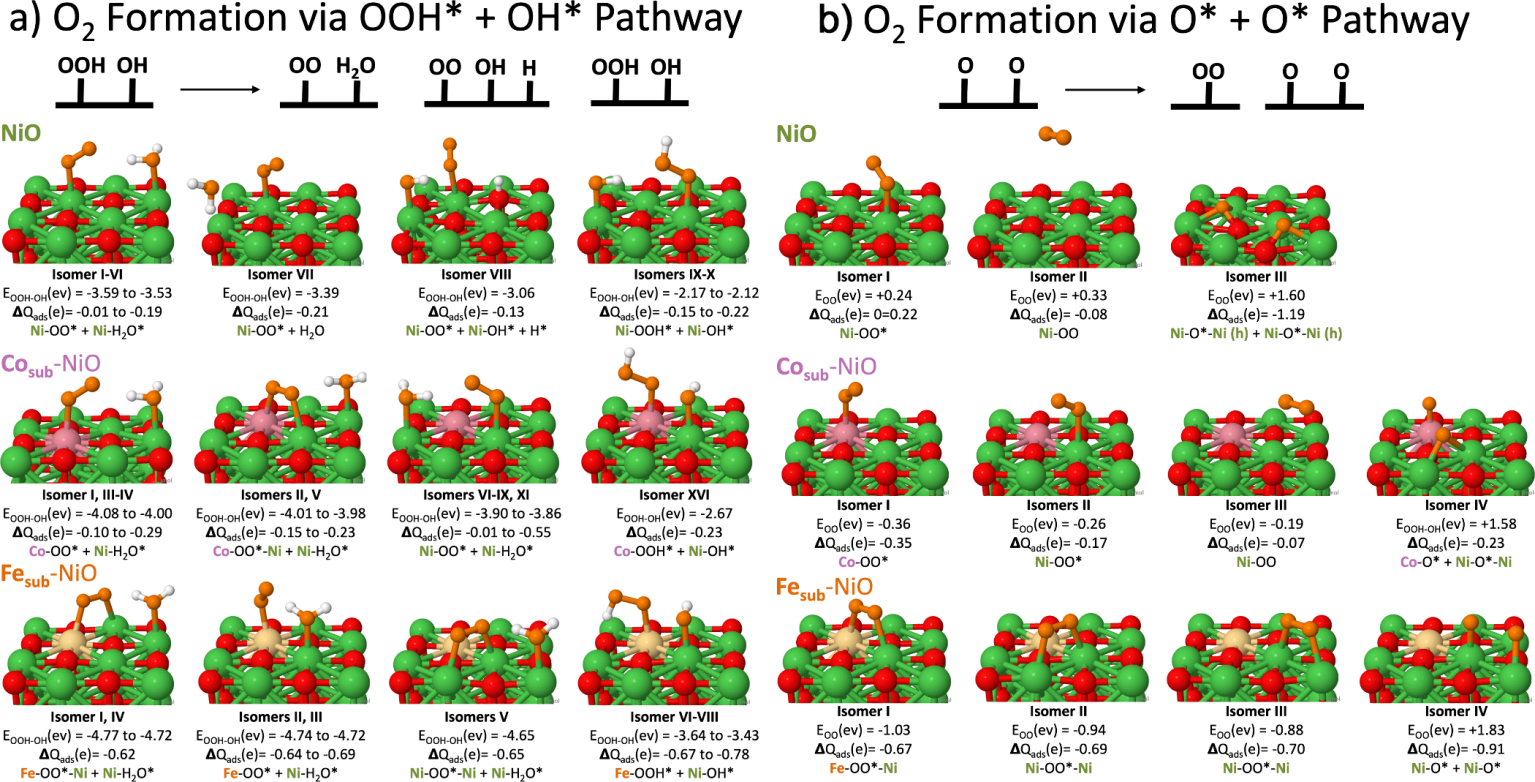
O_2_ Formation: (a) OOH* + OH* → OO* + H_2_O*, OO* + OH* + H*, or unreacted OOH*+ OH*. (b) O* + O* → O_2_* or unreacted O* + O*. All isomers are illustrated in detail in the SI. Ni atoms are in green; surface O—red; adsorbate O—orange; H—white; Co—pink; and Fe—yellow.

In our previous mechanistic study of IrO_2_ (110), we found multiple low-lying pathways to the dissociation of OH* and formation of O_2_* to be <0.4 eV when neighboring coadsorbed intermediates O_*x*_H_*y*_ were present. These pathways more accurately reflected the high reactivity of Ir-based materials observed by experiments as compared with the low-coverage pathway shown in the previous section. We examined in detail the OER mechanisms available to the NiO and doped-NiO to form O* via coadsorbed OH* + OH* (Figure 3) and O_2_* via the OOH* + OH* and O* + O* pathways (Figure 4), respectively. The coadsorbed mechanisms involving multiple O_*x*_H_*y*_ species more readily reflect the alkaline (pH 10–14) and high potential environment (1.6–2.2 V) of AEM electrolysis, where a mixture of surface O* and OH* is to be expected.^7,42,43^ Theoretical investigations of rutile oxides (RuO_2_, IrO_2_, TiO_2_) also identified the potential dependence of surface coverage: primarily OH* at <1.4 V; a mix of O* and OH* at potentials of 1.3–1.7 V; and primarily O* at >1.7 V.^44^

In Figure 3a, we showcase the various products that can arise from coadsorbed OH* + OH* on the NiO, Co_sub_-NiO, and Fe_sub_-NiO surfaces. We provide a line diagram in Figure 3b summarizing the relative energies of these products with respect to the global minimum structure, which are specifically accessible within the 2.0 V typical of electrolysis and in order of preferred OER products: namely, O* + H_2_O* followed by OOH* + H*, O* + H* + OH*, and HOOH*. We highlight in Figure 3c transition state calculations giving the activation energy for O* + H_2_O* formation on NiO and IrO_2_ as compared to the spontaneous, exothermic reaction on Fe_sub_-NiO. Bronoel et al.’s electrochemical study on NiO correlated the dependence of OH coverage to O_2_ formation: this may be due to the majority of the OH* being consumed to create the dominant product HOOH* and, secondarily, at ca. 0.6 V being utilized to form the preferred O* + H_2_O*. NiO’s activation energy is low at 0.10 eV compared to IrO_2_, but IrO_2_ is not reliant on OH* adsorption for the OER: IrO_2_ spontaneously splits water to form OH*, binds OH* more strongly than NiO, and remains active at both low and high coverages of O_*x*_H_*y*_.^16^ Fe_sub_-NiO advantageously makes O* + H_2_O* the dominant product with an exothermic reaction enthalpy of circa −0.5 eV.

These mechanistic trends may arise from the charge-transfer characteristics we observed in the previous section: NiO resists charge transfer to the key reaction intermediate OH* (Figure 2b). For coadsorption of 2OH*, this results in the surprising product of a peroxide HOOH* being the most stable on NiO, followed by OOH* + H*. Bader charge analysis reveals that both HOOH* (isomers I, II) and OOH* + H* (isomer III) products are nearly neutral with a negligible 0.04–0.05 e. In contrast, O* + H_2_O* often results in ca. 0.5–0.7 e from the surface to the adsorbates. For unreacted OH* + OH*, the charge transfer can range from 0.5 to 1.0 e to adsorbates. Similar to the bonding trends observed for OH* and O*, Co- and Fe-doped NiO surfaces are more oxophilic and promote charge transfer to adsorbate molecules: this stabilizes the O* + H_2_O* pathway, allowing O* + H_2_O* isomers to become the dominant product on Co_sub_-NiO (isomers I–III) and Fe_sub_-NiO (isomers I–III, IV).

We note that hydrogen peroxide (HOOH*) is both a competing product to the OER and potentially a poison to the membrane electrode assembly. Typically, HOOH decomposes in an alkaline environment, but trace amounts could react with the nitrogen-containing AEM ionomers, leading to possible degradation of these materials.^45−47^ In particular, HOOH is often used to oxidize amine- and pyridine-containing compounds into N-oxides.^47^ Trace amounts of Fe can catalyze the decomposition of hydrogen peroxide in alkaline environments into water and oxygen, which may be particularly advantageous for AEM electrolysis.^45,46^

In their electrochemical analysis of OER on a Ni electrode, Bronoel et al. considered five different mechanisms for OH* interacting with OH^—^, but a peroxide species was not a suggested product.^40^ Martirez et al. in their intensive, theoretical study of the possible mechanisms of OER on β-nickel oxyhydroxide and Fe-doped β-nickel oxyhydroxide only found this hydrogen peroxide species on the Fe-doped β-nickel oxyhydroxide.^13^ Moreover, the reaction free energy step versus RHE was situated at a limiting potential of 2.35 eV, considerably higher than those of other pathways. In contrast to our study of the OER mechanism, Martirez et al. focused on a proton-coupled electron transfer mechanism for their OER steps with H^+^ + e^—^ as the reference. Significantly, our results suggest that the crystalline phases of hydroxide versus rock-salt of Ni-based catalysts can yield radically different OER mechanisms.

With reference to the four-step OER mechanism often published in theoretical studies, we also considered O_2_ formation via the OOH* + OH* pathway or the O* + O* pathway (see Figure 4). As evidenced in Figure 3, the reactants OH* + OH* can yield OOH* + H* at moderate to high potentials: isomer III on NiO is 0.55 eV higher in energy; isomer XXVIII on Co_sub_-NiO is +1.34 eV; and isomer XXI on Fe_sub_-NiO is +1.70 eV more than the global minimum. While OOH* was the most stable on Fe_sub_-NiO in the low-coverage environment of the electrolyte-mediated mechanism in Figure 2, once coadsorption is considered, the OOH* pathway became the highest energy pathway on Fe_sub_-NiO. At moderate potentials, the OOH* pathway becomes more probable on the NiO surface due to the stabilizing effect of other coadsorbed species such as H*. However, as soon as there are neighboring OH* present (as can be expected in an alkaline environment), OOH* spontaneously deprotonates to form OO* and H_2_O* on both the NiO and doped-NiO surfaces (Figure 4a). Indeed, the intact OOH* coadsorbed with OH* isomers are often 1.1–1.4 eV higher in energy on the NiO and doped-NiO surfaces.

In Figure 4b, calculations of coadsorbed O* + O* on the NiO, Co_sub_-NiO, and Fe_sub_-NiO surfaces revealed that O_2_ spontaneously forms. In contrast, on IrO_2_ (110), the O* + O* → O_2_ requires an activation energy of 0.34–0.49 eV, depending on the surface coverage of the O*/OH* species.^16^ The OO* bond is ca. 1.2–1.3 Å on NiO, Co_sub_-NiO, and Fe_sub_-NiO, which complements the experimental gas-phase OO bond of 1.21 Å. The M(metal)–O bond for M-OO* on NiO, Co_sub_-NiO, and Fe_sub_-NiO is also elongated at 2.1–2.2 Å, allowing for easier desorption (desorbed isomers were ca. 0.1 eV higher in energy, SI Figure 25). This suggests that NiO may be deactivated for O_2_ formation early in the four-step OER process: it adsorbs OH* weakly in comparison to IrO_2_, and rather than forming O* and water, it produces the poisonous H_2_O_2_. However, if OH* binding is increased and O* + H_2_O* forms, then OER can become a thermodynamically downhill process since there is no barrier to O_2_ formation: we observe this trend the most strongly in Fe_sub_-NiO.

In conclusion, these theoretical calculations find that the bottlenecks to the OER activity of the OH* on NiO catalysts are (1) OH* adsorption and, possibly, (2) the HOOH* product, which may poison the ionomer and compete with the OER. Charge transfer from metal sites to the OER intermediates plays a significant role in affecting the binding strength and even directing mechanistic pathways to form the preferred O* + H_2_O*. The Co-dopant can increase OH* adsorption and even aid in the deprotonation of OH* to form O*, but at moderate potentials of ca. 0.6 V, it can still produce the poison HOOH*. The Fe dopant provides the greatest advantage to the OER: it strengthens OH* binding similar to IrO_2_; aids in the deprotonation of OH* to form O* + H_2_O*; and prevents the formation of the poison HOOH*. In the subsequent section, we will validate these theoretical predictions through experiment: manipulation of catalyst active sites through electrodeposition of metal electrodes and synthesis of nanoparticles, followed by characterization of these materials.

### II Experimental Validation and Characterization of Ni, Co–Ni, and Fe–Ni Catalysts

**Figure 5:**
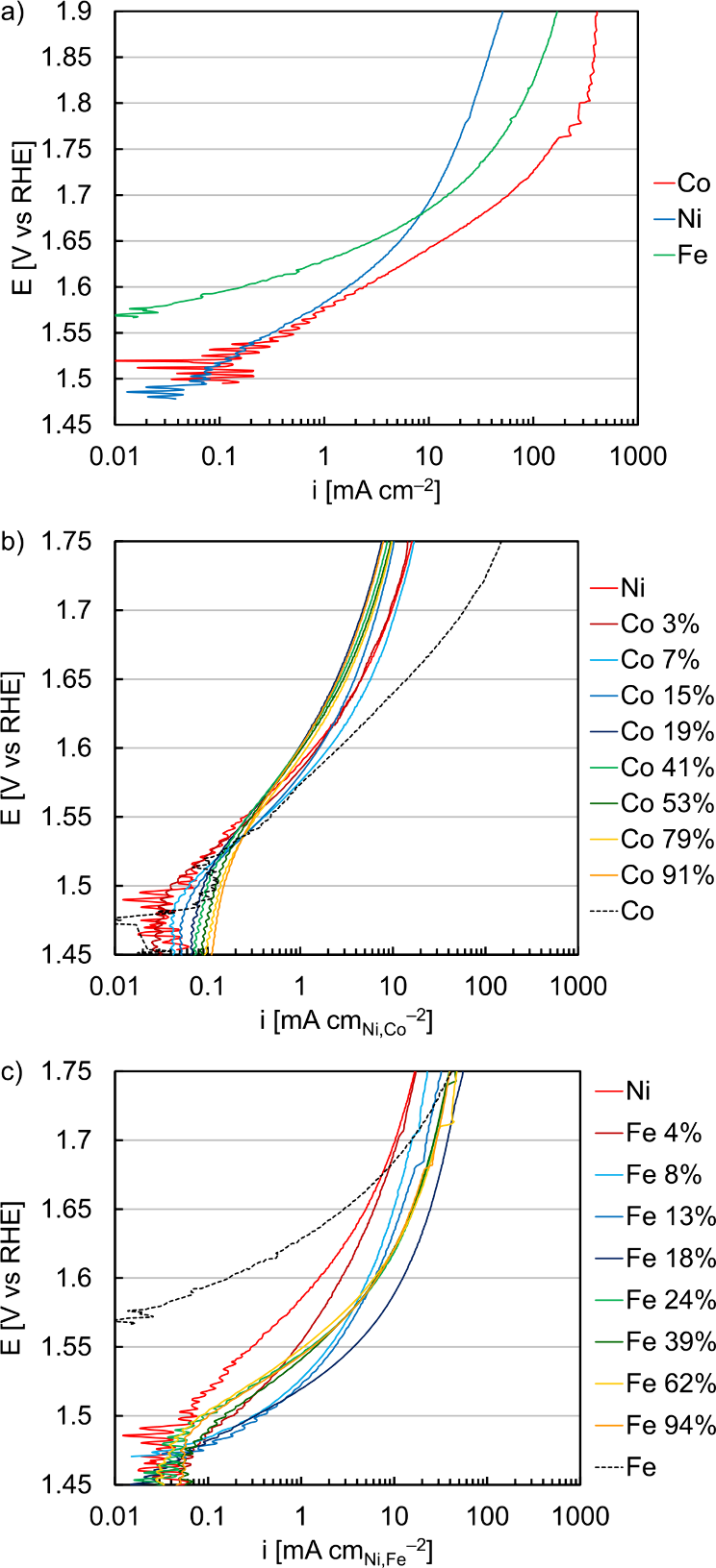
(a) Polycrystalline electrode data of Ni, Co, and Fe; (b) Co electrodeposited onto Ni; and (c) Fe electrodeposited onto Ni in pink and Fe in yellow.

To validate the findings from ab initio simulations, model surface compositions were tuned through electrodeposition and evaluated for the OER in basic electrolytes. On their own, Ni and Fe were unable to approach the activity of Co. Compared to a polycrystalline Co electrode, Ni quickly deviated at a relatively low current density (approximately 2 mA cm^–2^); Fe, at most, came within one-quarter of the activity in the kinetic region (Figure 5a).

The inclusion of Fe onto Ni surfaces, however, dramatically improved the OER kinetics (Figure 5b). For 18% Fe (82% Ni) and at 1.55 V, Fe–Ni exceeded the activity of Ni and Co by 11 and 9 times, respectively. The activity improvement then receded as the surface became Fe-dominant. In contrast, Co electrodeposition onto the Ni polycrystalline electrode did not enhance the kinetics of the OER (Figure 5b) but resulted in activities similar to or less than those of the Ni-only surface. These findings clearly indicate that the presence of Fe but not Co enhanced the OER kinetics of Ni surfaces. Ni deposited onto Co, Co onto Fe, and Fe onto Co combinations were also considered but yielded no improvements in activity (SI Figure 26). Ni–Co oxide catalysts in previous studies often underperformed compared to Fe–Ni or Co oxide.^6,8,11^

Electrochemical processes do not allow for the deposition of a spatially uniform monolayer of atoms. Surface area measurements calculated from redox transitions (Fe(II)/(III), Co(III/IV), Ni(III/IV)) provided insight into the degree of admetal–substrate interaction and indicated that at high degrees of Co/Fe deposition, the majority of admetal deposited onto itself instead of the Ni substrate (2.9 times for Co–Ni, 3.6 times for Fe–Ni, SI Figure 27). Heterogenous and incomplete coatings may add variability in specific OER activity determinations due to the persistence of Ni sites and the formation of Co/Fe sites absent in monolayers. While the use of heterogeneous electrochemical deposition introduced uncertainty in an optimal Fe–Ni composition, the presence of Ni and Fe sites in close proximity (below 20% Fe, compositions of relatively uniform depositions) clearly improved the OER activity, consistent with the improved oxophilicity and promoted charge transfer to adsorbate molecules, beyond the capabilities of the Ni surface, observed in theoretical calculations. At higher Fe contents, however, there were likely not enough Ni sites, and the lower OER kinetics of Fe contributed to this. While these studies focus on OER activity, there is likely a stability trade-off in Fe–Ni systems due to the increased mobility and dissolution rates of Fe.^48,49^

**Figure 6:**
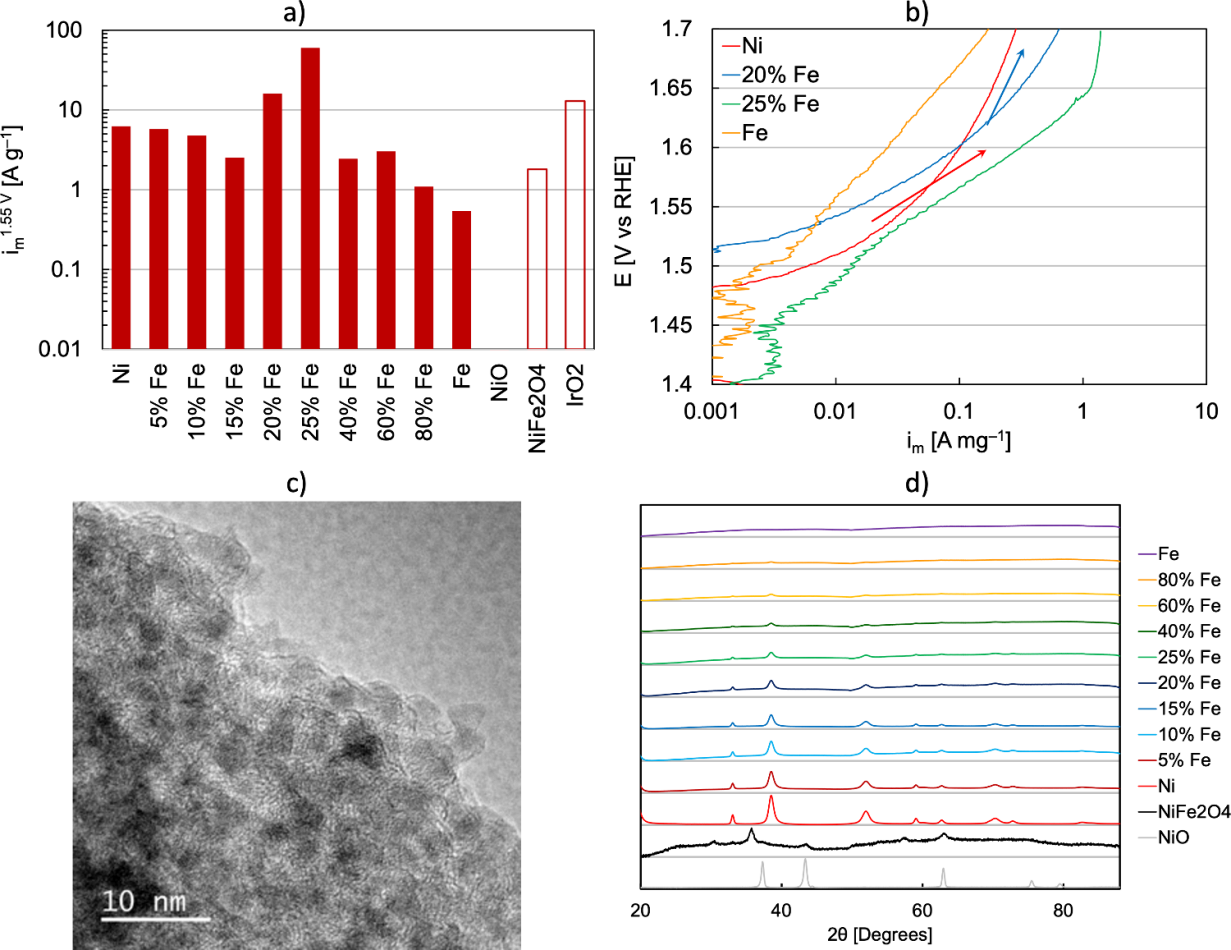
(a) Electrochemistry data of nanoparticles’ activity versus commercial benchmarks; (b) Tafel slope of Ni, Fe, and optimal Fe:Ni nanoparticle catalysts; (c) HRTEM of synthesized, high-performing Fe–Ni nanoparticles with 25% Fe based on ICP-MS; and (d) XRD of commercial catalysts and synthesized nanoparticles.

To build upon the fundamental findings on model surfaces, hydrothermal synthesis was used to form Fe–Ni nanoparticle catalysts with varying compositions (Figure 6a,b). As with the surfaces, a moderate Fe content improved the OER activity at the optimum nanoparticles and a commercial NiFe_2_O_4_ benchmark catalyst by 10 and 40 times, respectively. The optimum (25%) Fe–Ni exhibited 59.7 A/g, IrO_2_ exhibited 12.9 A/g, and RuO_2_ exhibited 44.6 A/g, correlating to a 4.6 times improvement to IrO_2_ and a 34% improvement to RuO_2_. For each catalyst evaluated in RDE testing, deviations were observed at a high current density that indicated the onset of transport losses. For Ni, however, the deviation from 70 mV dec^–1^ occurred at a relatively small current and suggested activity limitations due to a kinetic process as opposed to transport (Figure 6b). Fe doping further enabled higher performance at moderate current density and suggested mechanistic differences between the Ni-only and Fe-doped catalysts. These results may support theoretical calculations in the previous section that found Fe_sub_-NiO could redirect the hydrogen peroxide pathway present on NiO to the preferred O* + H_2_O* necessary for OER. The O* formation step is the rate-limiting step on NiO, but this can be overcome by the inclusion of Fe; moreover, O_2_ formation is spontaneous on Fe_sub_-NiO. XRD and microscopy confirmed relative zone segregation, and codeposition did not appear to create significant alloying. The presence of Fe, however, clearly improved the Ni-OER activity; as with the surfaces, the OER activity decreased as the Fe became too prevalent.

We note that commercial NiO is significantly less active compared with other catalysts, even though HRTEM detected the presence of defects (Figure 1c). For the high-performing (25%) Fe–Ni nanoparticles, TED (SI Figure 28) showcased a nanocrystalline material with lattice spacings close to Ni metal. HRTEM revealed larger particles composed of an agglomeration of small, randomly oriented crystalline particles (Figure 6c). EDS found a substantial amount of O present in some areas of the Fe–Ni nanoparticles, which would support the theoretical model focusing on a metal oxide surface (SI Figure 29). For the synthesized nanoparticles, XRD confirmed a crystalline Ni hydroxide phase that was distinct from the NiO and NiFe_2_O_4_ commercial benchmarks, with a size of ca. 120 Å, which did not appreciably change with composition (Figure 6d). The Fe, however, did not clearly appear in XRD and likely indicated a significantly smaller crystallite size or an amorphous structure altogether. The inhomogeneity of mixed-metal materials depending on the synthesis method or commercial sample is well-known: there can be a mixture of different crystalline phases, varying ratios of metal and metal oxide contents, and a range of exposed facets.^6,8−12^ These inhomogeneities can even be advantageous for manipulating the binding strength of key HER/OER intermediates in mixed-metal and mixed-metal oxide materials.^19,37,50^ The significant increase in activity of the synthesized nanoparticles as compared to commercial catalysts may originate from their inhomogeneities and the advantageous size and edge effects not present in commercially available catalysts. In general, the commercial products examined were more crystalline and exhibited much larger particle sizes when imaged in TEM compared to the synthesized nanoparticle samples.

## Conclusions

The inclusion of Fe can radically improve performance as observed for commercial NiFe_2_O_4_, but the nuanced manipulation of the Fe:Ni ratio via electrodeposition and the synthesized nanoparticles showcase that ca. 20% Fe (18% Fe for electrodeposited Fe onto Ni; 20–25% Fe in Fe–Ni nanoparticles) may be key to a non-PGM catalyst surpassing PGM IrO_2_ in performance. In theoretical calculations on a model (100) NiO rock-salt surface with 12.5% Fe and 87.5% Ni surface site access, Fe sites preferentially formed O* + water and Fe_sub_-NiO could spontaneously form O_2_ either through coadsorbed O* + O* or through deprotonation of the OOH* pathway by OH. This joint theoretical-experimental study showcases how radically the chemistry of a relatively inactive material, NiO, can mimic or even surpass the bonding trends and activity present in the benchmark PGM catalyst IrO_2_. This has immense ramifications in hydrogen technologies, which require a cheaper, more active catalyst at lower current densities to reach worldwide energy objectives such as the United States’ Hydrogen Shot Goal of $1/kg H_2_ in one decade, the European REPowerEU Plan’s for domestic renewable hydrogen production of 10 million tonnes by 2030, and Japan’s Green Growth Strategy to reduce hydrogen costs by one-third in the same time period. Fundamental understanding of reaction mechanisms and subsequent manipulation of the rate-determining steps have far-reaching implications for catalysis: here, theory demonstrated that the inactive NiO can become a superior OER catalyst through an Fe dopant, comparable in activity to the PGM IrO_2_ catalyst; these same strategies can potentially be employed to improve activity in other applications such as CO_2_ reduction for selectivity of high value products, e.g. acids, alcohols, and hydrocarbons, and the nitrogen reduction reaction for ammonia to make it more competitive with the Haber–Bosch process.

## Methods

### Theoretical

Projector augmented wave^51,52^ pseudopotentials with the Perdew–Burke–Ernzerhof (PBE)^53^ functional and Hubbard U^54^ corrections were implemented utilizing the plane-wave DFT code and the Vienna ab initio simulation package (VASP 5.4.4).^55−58^ Wang et al. recommended *U* = 6.4 for Ni, *U* = 4.0 for Fe, and *U* = 3.3 for Co to reproduce the calculated oxidation energies of transition metal oxides;^59^ these *U*-values in the rock-salt antiferromagnetic crystals moreover produced band gaps relatively close to the experimental values (SI Table 1).^54,60,61^ Dopant incorporation was evaluated at various sites (substituted, interstitial, and adsorption).

For surface calculations, in order to more realistically reflect the experimental conditions, dispersion corrections and implicit solvation (VASPsol) were implemented.^62−64^ Adsorbates from a previous work on the OER mechanism on IrO_2_ (110) were recalculated with the PBE pseudopotential in conjunction with dispersion corrections and implicit solvation for comparison with the NiO and doped-NiO calculations. Subsequently, the calculated adsorption energies utilized the following equation:
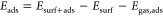
where *E*_surf+ads_ is the total energy of the surface with the adsorbate, *E*_surf_ is the total energy of the clean surface without the adsorbate, and *E*_gas,ads_ is the total energy of the gas-phase adsorbate (OH, O, OOH, O_2_). The starting OH*/OOH* was positioned vertically and horizontally to the surface and rotated every 45° on atomic, hollow, and bridging sites in order to find the numerous local minima relevant to the high potentials of the OER.

We evaluated these local minima utilizing the Boltzmann probability (*P*_*i*_), where the Boltzmann distribution of each local minimum (*e*^–*E*_*i*_/*k*_B_*T*^) was divided by the summation of the local minima at 300 K:
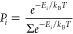
where *T* is the temperature at 300 K, *k*_B_ is the Boltzmann constant, and *E*_*i*_ is the *i*th energy of a local minimum for the adsorbed species. At 300 K, a number of local minima may be populated at the interface. This guided the setup of singly adsorbed OH* to coadsorbed OH*+OH*, where only OH* on neighboring atomic sites were set up. Singly adsorbed OH* on a metal site would be 99–100% populated at room temperature. A minimum of four OH* or OOH* isomers were used as starting points for coadsorption, with additional OH* placed at neighboring metal sites. Postprocessing of isomers was performed, extracting electronic information for bonding analysis such as charge transfer via the Bader charge algorithm.^65−69^

## Experimental Section

Catalysts evaluated for alkaline OER in RDE half-cells included polycrystalline electrodes, admetals electrodeposited onto polycrystalline electrodes, synthesized nanoparticles, and commercial nanoparticle benchmarks. Polycrystalline electrodes included cobalt (Co), nickel (Ni), and iron (Fe) purchased from Pine Instrument Company with a diameter of 5 mm (0.196 cm^2^ electrode area). Commercial nanoparticle benchmarks included Co_3_O_4_ (Alfa Aesar, 44661), NiO (Alfa Aesar, 10819), NiFe_2_O_4_ (US Research Nanomaterials Inc., US3959), IrO_2_ (Alfa Aesar, 43396), and RuO_2_ (Alfa Aesar, 11804).

Electrodeposition of Co and Fe onto Ni utilized the polycrystalline Ni electrode (Pine Instrument Company) as the substrate and modified protocols from McCrory et al.^6^ The Co deposition solution contained 0.76 g of Co sulfate hydrate (CoSO_4_·7H_2_O, Sigma-Aldrich, 99.998%) and 0.62 g of ammonium perchlorate (NH_4_ClO_4_, Sigma-Aldrich, 99.999%) in 150 mL of distilled, deionized water (18.2 MΩ, TOC < 4 ppb), titrated to pH 6.8 with a solution of ammonium hydroxide (Sigma-Aldrich, OmniTrace Ultra). The Fe deposition solution contained 0.38 g of Fe sulfate hydrate (FeSO_4_·7H_2_O, Sigma-Aldrich, 99%) and 0.44 g of ammonium sulfate ((NH_4_)_2_SO_4_, Sigma-Aldrich, ReagentPlus) in 150 mL of distilled, deionized water (18.2 MΩ, TOC < 4 ppb), titrated to pH 2.5 with solutions of ammonium hydroxide (Sigma-Aldrich, OmniTrace Ultra) and 1 M sulfuric acid (H_2_SO_4_, ACS grade).

For electrodeposition experiments, the Ni polycrystalline electrode was inserted into a ChangeDisk Tip (Pine Research Company, AFE5TQ050) and then into glassware with the deposition solution, previously cleaned by submersion overnight in sulfuric acid and Nochromix baths, and then boiled eight times in distilled, deionized water (18.2 MΩ, TOC < 4 ppb).^70^ RDE half-cells further contained a platinum (Pt) wire/mesh counter electrode and a reversible hydrogen electrode (RHE) connected to the main cell by a Luggin capillary. Working electrodes were held at a current of −10 mA cm^–2^ for a variable time at 2500 rpm with an Autolab PGSTAT302N potentiostat (Metrohn Autolab) and Nova 2.1 software. The Ni polycrystalline electrode was then immediately removed from the deposition solution, rinsed with distilled, deionized water, and air-dried three times.

Ni–Fe nanoparticles were synthesized hydrothermally.^71,72^ Mixtures of Ni and Fe acetate hydrate (Sigma-Aldrich, 99.995%) were dissolved in 1.2 mL of distilled, deionized water. Ethanol (13.8 mL) and then ammonia (25%, 2.5 mL) were added dropwise while stirring, and the mixture was heated to 150 °C for 3 h in a 45 mL stainless steel autoclave (Parr Instrument Company). Post synthesis, the nanoparticles were washed 3 times in ethanol.

For RDE half-cell evaluation, 3.49 mg of each nanoparticle catalyst was added to 7.6 mL of distilled, deionized water and 2.4 mL of isopropanol. Inks were iced for 5 min, and then 20 μL of a 5 wt % Nafion dispersion was added. Inks were horn sonicated for 30 s, bath sonicated for 20 min, and horn sonicated for 30 s, all in ice. Inks (10 μL) were pipetted onto inverted gold polycrystalline electrodes rotating at 100 rpm. After pipetting, the rotation was increased to 700 rpm, and the electrodes were allowed to dry in air and at room temperature for 20 min.

All electrodes and catalysts were evaluated for alkaline OER in a polytetrafluoroethylene cell (Pine Instrument Company) containing a gold mesh/wire counter electrode, a mercury/mercurous oxide reference electrode (Koslow Scientific) connected to the main cell by a custom Lugging capillary, and 0.1 M sodium hydroxide (NaOH, Sigma-Aldrich, TraceSelect).^7,11,73^ Prior to testing, the reference electrode was calibrated with a polycrystalline Pt electrode (Pine Instrument Company) rotating at 2500 rpm in a hydrogen-saturated electrolyte during cyclic voltammograms at 10 mV s^–1^, approximately in the potential range of −0.1 to 1.0 V vs RHE. Calibration to RHE was defined as the potential intercept between hydrogen oxidation and evolution, averaged between anodic and cathodic scans. The Pt polycrystalline electrode was then held at −0.5 V vs RHE in a nitrogen-saturated electrolyte for 900 s to electrochemically plate metal contaminants (particularly Fe) prior to testing.^74,75^ After electrode/catalyst working electrodes were inserted, conditioning was completed by 50 cycles in the potential range of 1.2–1.8 V vs RHE (50 mV s ^–1^) at 2500 rpm; OER activities were determined through linear sweep voltammograms at 20 mV s ^–1^ in the same potential range at 2500 rpm. OER activities were corrected during the experiment for the internal resistance drop (typically 23–25 Ω) through a built-in current interrupter at 1.6 V vs RHE.

Cyclic voltammograms were taken at 20 mV s ^–1^ in the potential range of 0–1.4 V vs RHE. For electrodeposited surfaces, quantifying the surface composition was determined from the redox transition charge responses of Ni, Co, and Fe on their respective polycrystalline electrodes—Fe(II)/(III), Co(III/IV), and Ni(III/IV).^76^ In Table 2, charge responses were integrated during anodic scans (oxidation) to avoid the impact of the upper scan limit on the reduction charges. To normalize the OER activity of electrodeposited surfaces to the number of sites, approximate electrochemical surface areas were determined from these transition charges relative to the single-element electrodes, assuming a roughness factor of 1.2. For nanoparticle catalysts, electrochemical surface areas were not determined due to the higher oxide content and the impact of oxides on charges from redox transitions.

**Table 2:** Charge Responses of Co on Ni and Fe on Ni

time held (s)	Co on Ni (% Q)	Fe on Ni (% Q)
5	3	4
10	7	8
15	16	13
20	19	18
40	41	24
60	53	39
100	79	62
120	91	94

Nanoparticle catalyst compositions were determined by inductively coupled plasma mass spectrometry (ICP-MS), taken with a Thermo Scientific iCAP Q. The ICP-MS was calibrated to a blank, internal standard and three Ni, Co, and Fe standards (2, 20, and 200 ppb). ICP-MS data were further taken with a dwell time of 0.15 s and a standard deviation of less than 2% between the measurements.

XPS data were obtained on a Physical Electronics 5600 system using Al Kα radiation. The XPS setup was calibrated with Au metal, which was cleaned via Ar-ion sputtering. The raw atomic concentration has a 5% error due to surface inhomogeneities, surface roughness, and literature sensitivity values for peak integration. Transmission electron microscopy (TEM) samples were prepared by dispersing the catalyst powders onto ultrathin carbon films on lacey carbon TEM support grids. TEM, TED, and EDS were performed on an FEI Tecnai ST30 TEM operated at 300 kV. EDS quantification was performed using the EDS analysis program embedded in FEI TIA software.

## Supporting Information Available

Detailed theoretical data of all isomers and experimental information regarding synthesis and characterization (PDF)

## Abbreviations

OER: oxygen evolution reaction
AEM: anion-exchange membrane
DFT: density functional theory
PGM: platinum group metal
RDE: rotating disk electrode
MEA: membrane electrode assembly
iso: isomer
HRTEM: high-resolution transmission electron microscopy
TED: transmission electron diffraction
EDS: energy-dispersive X-ray spectroscopy
XPS: X-ray photoelectron spectroscopy
XRD: X-ray diffraction
