# Correction to “Fe-Doped Ni-Based Catalysts Surpass Ir-Baselines for Oxygen Evolution Due to Optimal Charge-Transfer Characteristics”

**DOI:** 10.1021/acscatal.5c00322

**Published:** 2025-01-27

**Authors:** Mai-Anh Ha, Shaun M. Alia, Andrew G. Norman, Elisa M. Miller

The updated Acknowledgments are given below.

