# Supplementary material for: Fe-Doped Ni-Based Catalysts Surpass Ir-Baselines for Oxygen Evolution Due to Optimal Charge-Transfer Characteristics
Source: ACS Catal. 2024 Nov 11;14(23):17347–59. doi: 10.1021/acscatal.4c04489 (PMC11629292; doi:10.1021/acscatal.4c04489)
Supplement: Supplementary file 1 — cs4c04489_si_001.pdf [file cs4c04489_si_001.pdf]

Fe-doped Ni-based Catalysts Surpass Ir-baselines for Oxygen Evolution Due to Optimal Charge-Transfer Characteristics

Mai-Anh Ha,*[a] Shaun Alia,[b] Andrew Norman[c] and Elisa Miller[b]

[a.] *Computational Science Center, National Renewable Energy Laboratory, 15013 Denver West Parkway, Golden, Colorado 80401, United States.*
**E-mail: MaiAnh.Ha@nrel.gov*
[b.] *Chemistry and Nanoscience Center, National Renewable Energy Laboratory, 15013 Denver West Parkway, Golden, Colorado 80401, United States.*
[c.] *Materials Science Center, National Renewable Energy Laboratory, 15013 Denver West Parkway, Golden, Colorado 80401, United States.*

Supporting Information

Table 1. Bulk Properties

Bulk	Space Group	L.C. (Å)	$\lvert\mu_{B,avg}\rvert$	$\lvert\mu_B\rvert_{exp}$	$\Delta q_{M,avg}$ (e)	$\Delta q_{O,avg}$ (e)	B_g	$B_{g,exp}$	U
NiO	Fm-3m	4.17	1.76	1.64-1.90	1.27	-1.27	4.12	4.2	6.4
CoO	Fm-3m	4.26	2.66	3.35-3.80	1.31	-1.31	2.39	2.4	3.3
FeO	Fm-3m	4.31	3.66	3.32	1.27	-1.27	2.66	2.4	4

Table 2. Surface Properties

Surface	Space Group	L.C. (Å)	$\lvert\mu_{B,avg}\rvert$	$\lvert\mu_B\rvert_{exp}$	$\Delta q_{M,avg}$ (e)	$\Delta q_{O,avg}$ (e)	U
NiO	Fm-3m	4.17	1.76	1.64-1.90	1.24	-1.24	6.4
CoO	Fm-3m	4.26	2.64	3.35-3.80	1.24	-1.24	3.3
FeO	Fm-3m	4.31	3.68	3.32	1.24	-1.24	4

Table 3. M_{sub}-doped NiO Surface Properties (ck)

Surface	L.C. (Å)	$\lvert\mu_B\rvert$ Ni	$\lvert\mu_{B,avg}\rvert$ Ni	$\lvert\mu_B\rvert$ M	Δq_{Ni} (e)	$\Delta q_{Ni,avg}$ (e)	Δq_M (e)	U_M
NiO	4.17	1.76-1.77	1.76	-	1.21-1.25	1.24	-	6.4
Co-NiO	4.17	1.76-1.77	1.76	2.68	1.21-1.26	1.24	1.29	3.3
Fe-NiO	4.17	1.75-1.77	1.76	3.69	1.18-1.26	1.24	1.26	4

a) Interstitial Site

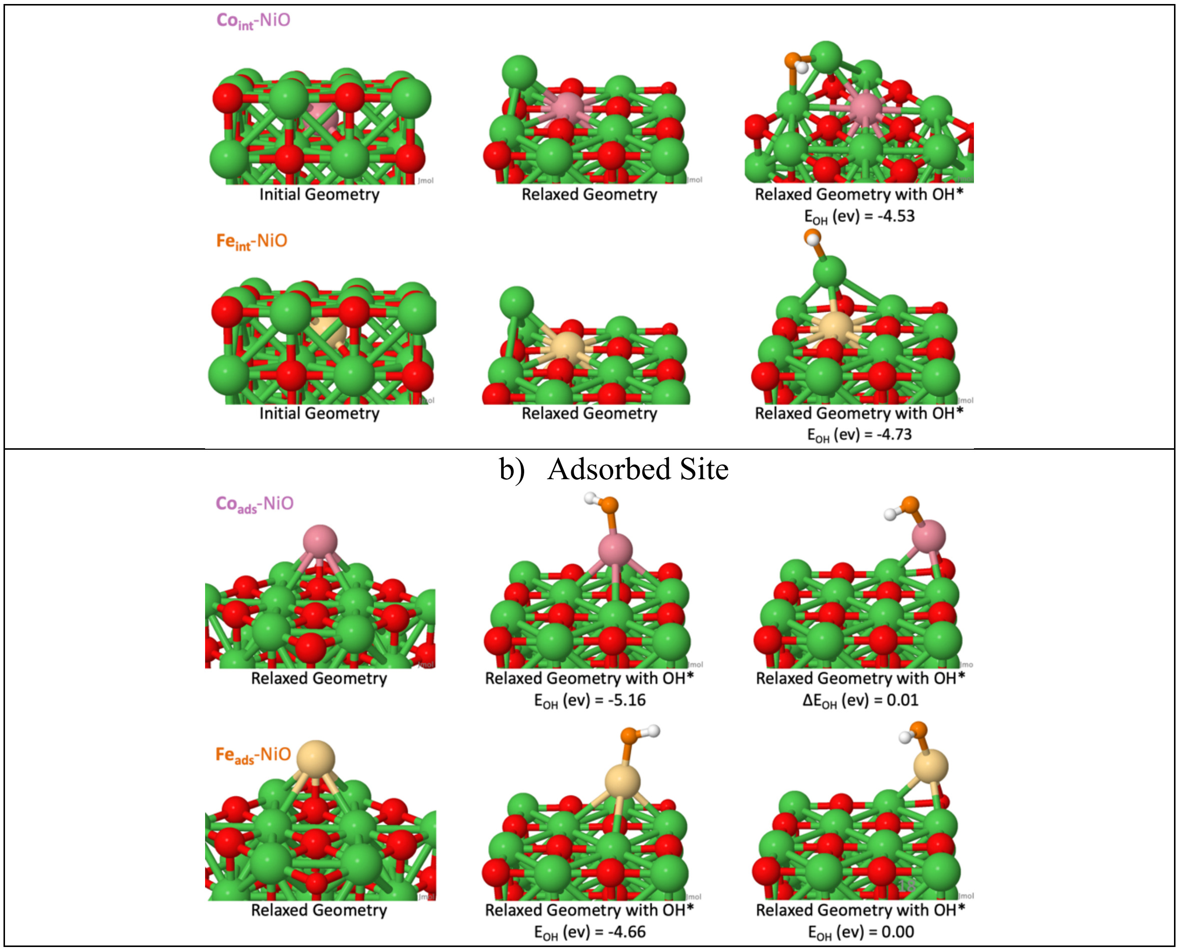

Figure 1. Other possible dopant sites and their relative stabilities: a) interstitial site relaxed to have the dopants in a pseudo-substitution site; upon adsorption of OH*, the dopant metal substituted Ni; b) the adsorbed site exhibited low enthalpy for the mobility of the dopant as a monomer upon OH* adsorption.

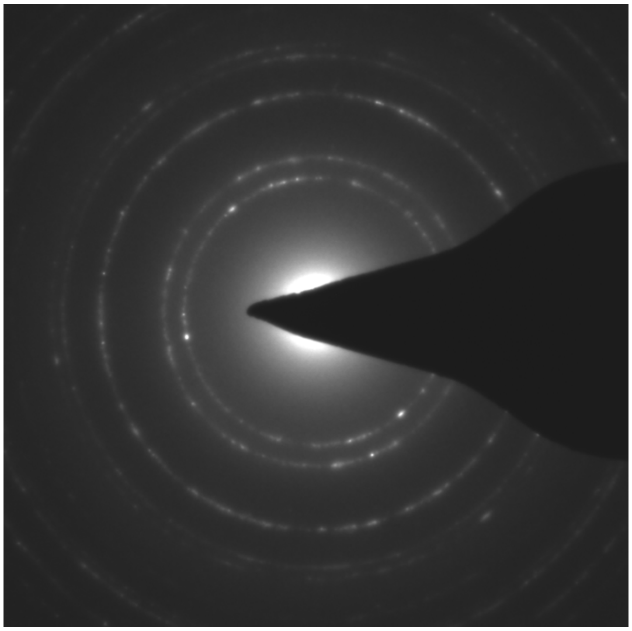

Figure 2. Transmission electron diffraction (TED) of **commercial** NiO catalysts.

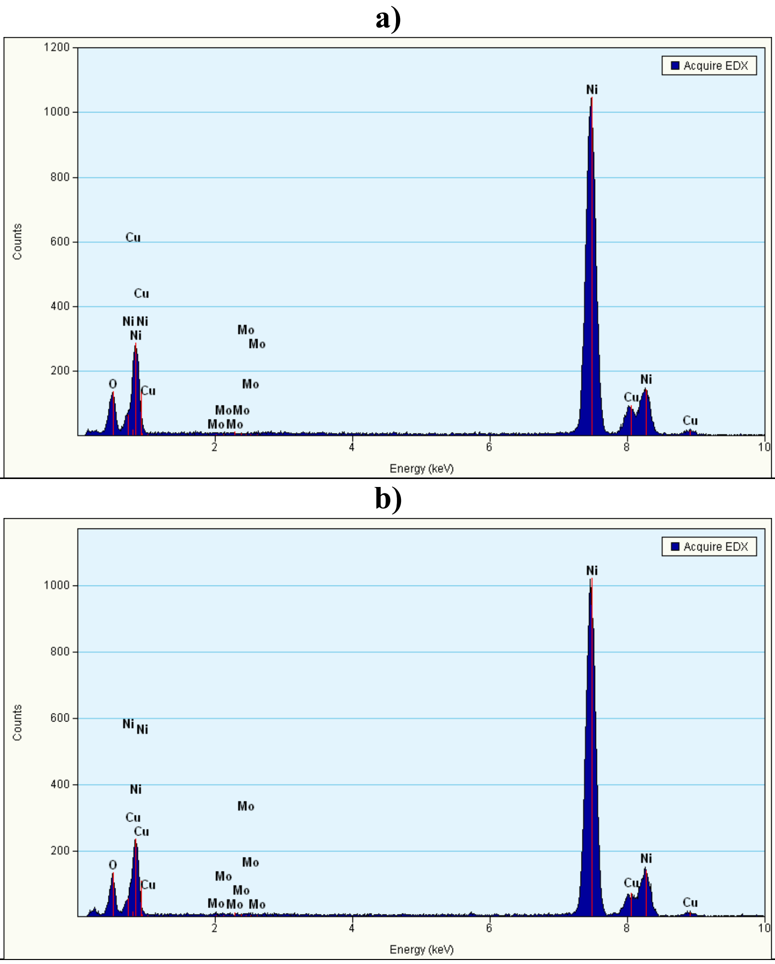

Figure 3. Energy-dispersive X-ray spectroscopy (EDS) data of commercial NiO catalysts.

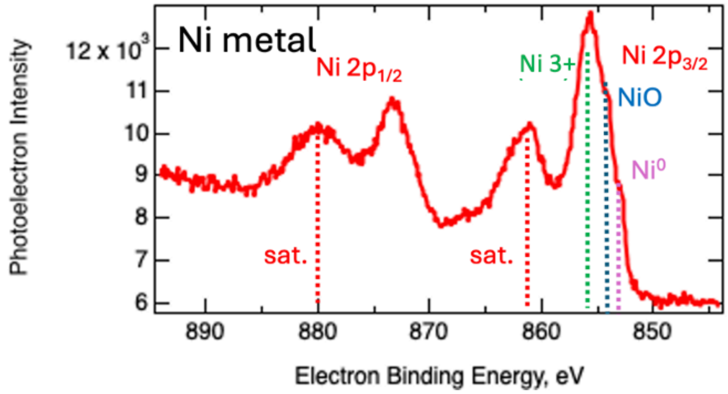

Figure 4. XPS Data of commercial Ni metal.

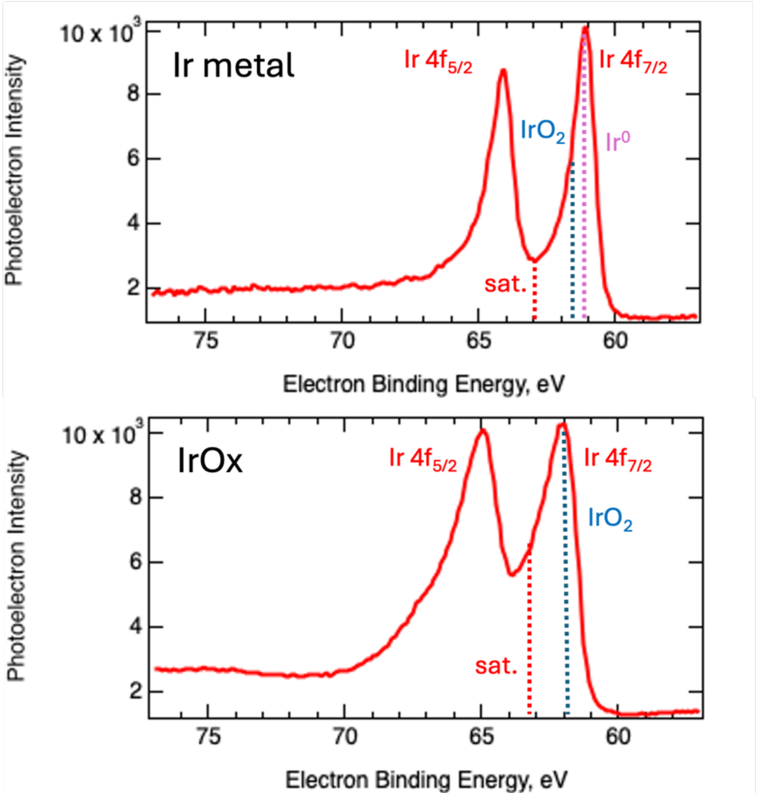

Figure 5. XPS Data of commercial Ir metal (top) and IrO_x (bottom).

XPS Experimental Discussion

For the Iridium XPS data, the overlayed spectra clearly show that the Ir(JM) spectrum (red) has narrower peaks at lower binding energy compared to the IrOx (AA) spectrum.

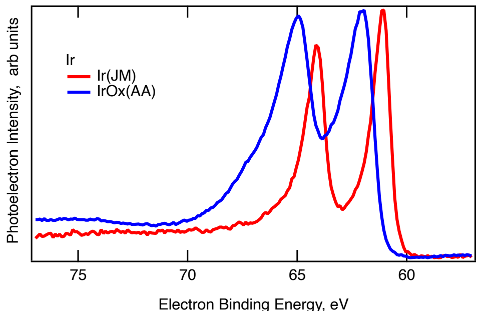

Figure 6. Iridium XPS data of Ir(JM) and IrOx (AA).

The fit peak positions are consistent with Ir metal (blue trace - 61.6 eV our data vs. 60.80 eV reference Freakley et al).[1] The difference between our data and the reference is that our samples are measured as-received and are not sputtered clean. Therefore, the amount of surface oxidation will shift the peak positions from Ir metal references. The assignment to the IrO_2 peak positions (green traces 61.76 eV Ir(JM) and 62.13 eV IrOx(AA) vs. 61.9 - 62.5 eV by Freakley et al.) are very close to this literature value.[1]

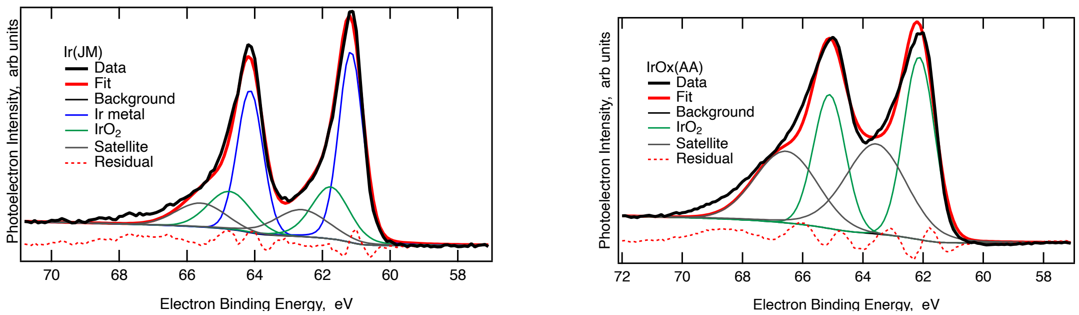

Figure 7. XPS Fits of Ir(JM) and IrOx (AA).

For the Ni XPS spectra, we also see Ni metal in the commercial sample, but it is quite small. From the overlayed Ni XPS data, the slight shift and shoulder due to Ni metal (blue trace) can be seen compared to the NiOx (red trace).

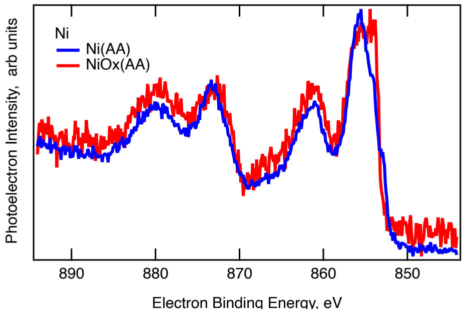

Figure 8. XPS of Ni (AA) and NiOx (AA).

The peak fit for Ni metal (blue trace) in the Ni(AA) sample is close to reference (blue trace 852.81 eV vs 852.60 eV by *Biesinger et al.*).[2] The difference is likely due to the surface oxidation since this sample was measured as-received and not sputtered clean. The NiO peak positions (green traces 854.2 eV Ni(AA) and 854.12 eV NiOx(AA) vs 853.7 eV by *Biesinger et al.*) are also close and

likely different due to the different surface oxidation.[2] The cyan traces assigned to NiO(OH) is close to 856 eV for both samples. This is 1 eV higher than the reference value.[2] **Therefore, we will change this assignment to Ni+3 since we cannot convincingly assign as NiO(OH).**

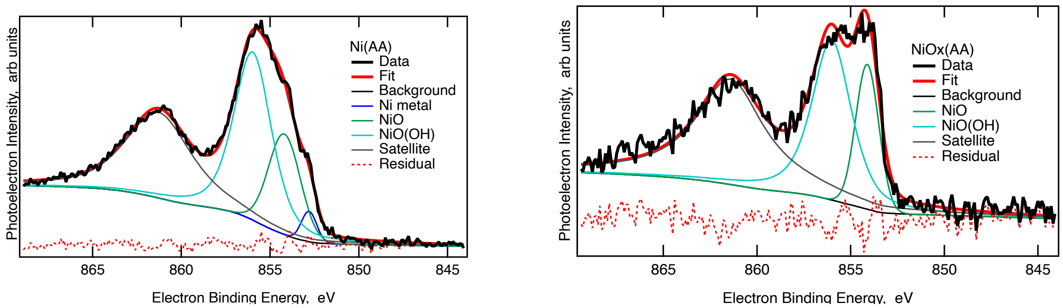

Figure 9. XPS fits of Ni (AA) and NiOx (AA).

Additional Theory Discussion

In deference to our readers' interest in free energies, we also provide here the free energy versions of figures and certain OER minima:

$$\Delta G = \Delta(E + ZPE) - T\Delta S$$

Where the total energy of an adsorbed O_xH_y intermediate on the relevant surface or gas phase adsorbate is E. The zero-point energy (ZPE) was calculated from vibrational frequency calculations of O_xH_y intermediates adsorbed on the surface (only frequencies of global minima structures were calculated):

$$\text{ZPE} = \sum_{i=1}^{n} \frac{1}{2} h v_i$$

The contributions due to the vibrational entropies of adsorbed O_xH_y intermediates was also evaluated:

$$S_{vib} = k_B \sum_{i=1}^{n} \left[\frac{hv}{k_B T(e^{\frac{hv}{k_B T}} - 1)} - \ln\left(1 - e^{\frac{-hv}{k_B T}}\right) \right]$$

For reference molecules O_2, OH, and H_2O we utilized NIST tables for the standard entropy.[3] For the difference surfaces, the free energy was the enthalpy or total energy of the surface. The reaction profile is as follows, depending on the specific intermediates present on the different surfaces:

$$G_{surf} + G_{OH} \rightarrow G_{OH*}$$

$$G_{OH*} \rightarrow G_{O*+H*}$$

$$G_{O*+H*} + G_{OH} \rightarrow G_{O*} + G_{H2O}$$

$$G_{O*} + G_{OH} \rightarrow G_{O*+OH*} \text{ (or } G_{OOH*}\text{)}$$

$$G_{O*+OH*} \text{ (or } G_{OOH*}\text{)} \rightarrow G_{OO*+H*}$$

$$G_{OO^*+H^*} + G_{OH} \rightarrow G_{OO} + G_{H2O}$$

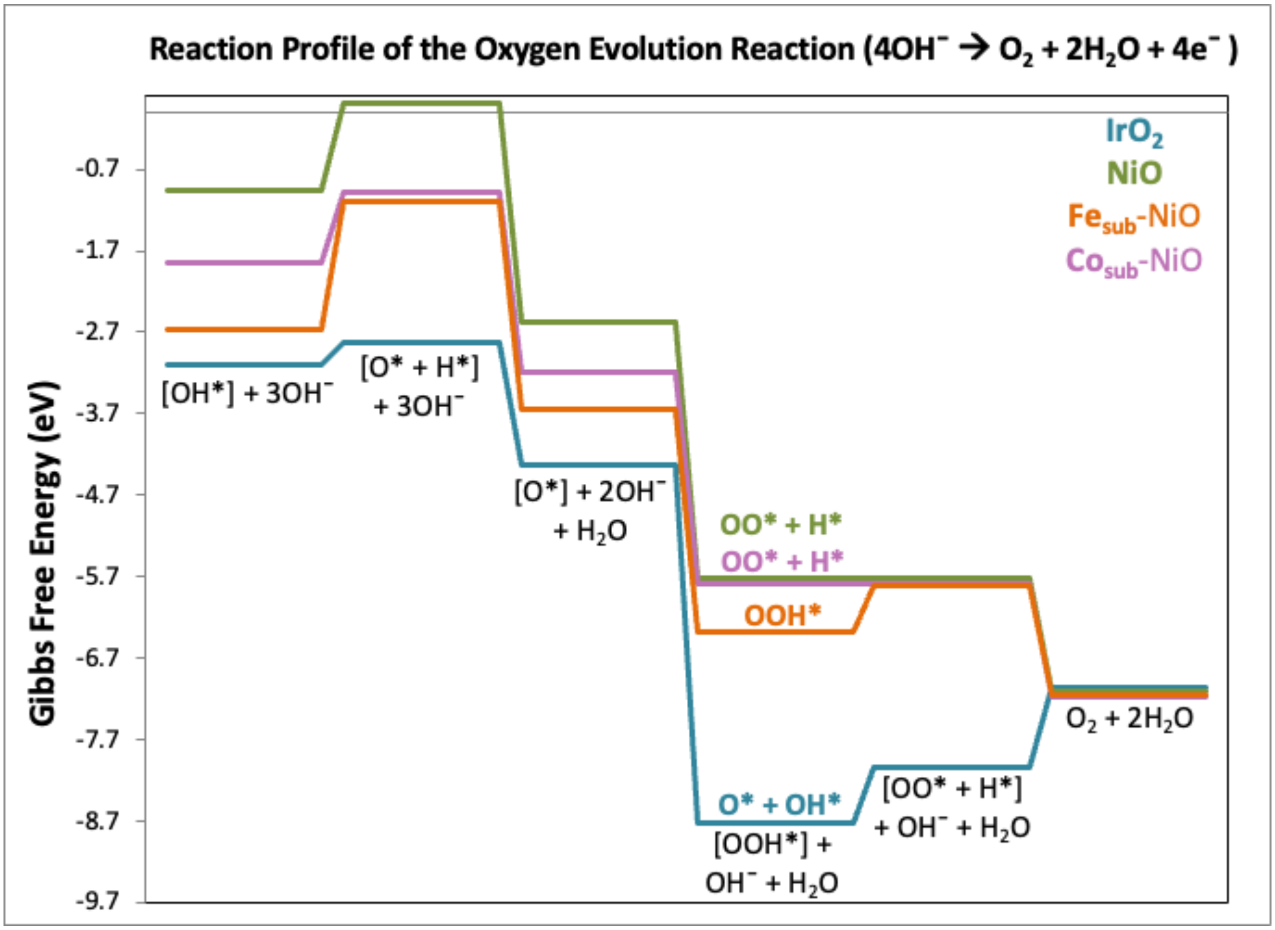

Figure 10. Gibbs Free Energy Reaction Profile of the oxygen evolution reaction on various surfaces.

In comparison to the total energies of these intermediates, the trends remain the same. However, free energy of adsorption for G_{OH} is now -0.96 for NiO, -3.10 for IrO_2, -1.84 for Co_{sub}-NiO, and -2.68 for Fe_{sub}-NiO.

**OH*** **I** (ΔE=0.00)	**OH*** **II** (ΔE=0.00)	**OH*** **III** (ΔE=0.06)	**OH*** **IV** (ΔE=0.23)
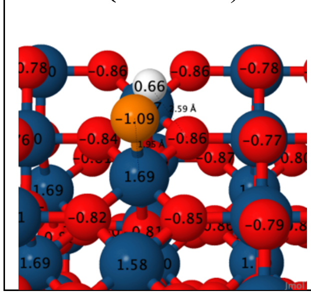	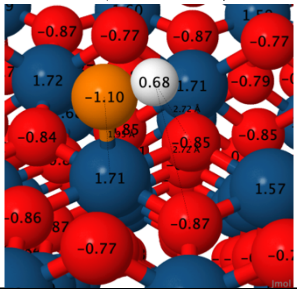	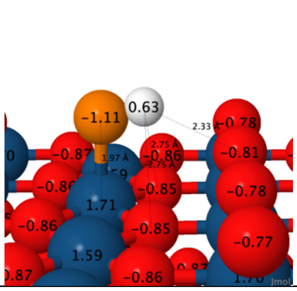	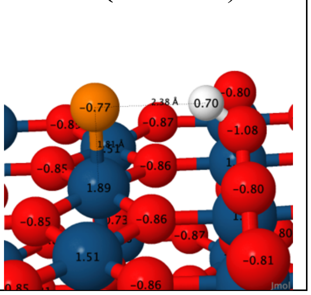

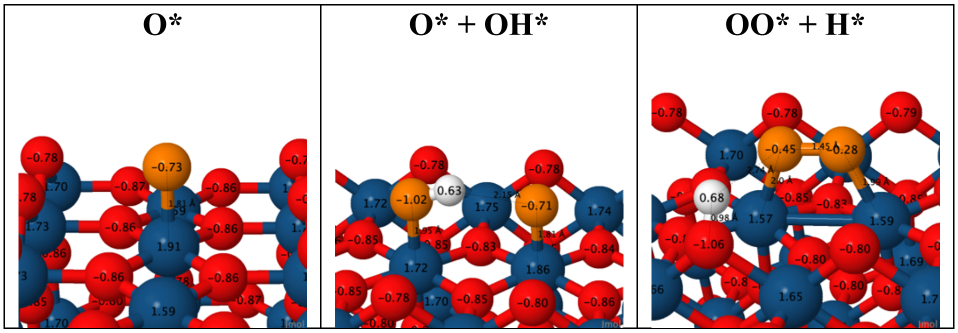

Figure 11. Lowest minima of adsorbed O_xH_y* isomers on the IrO_2 (110) surface.

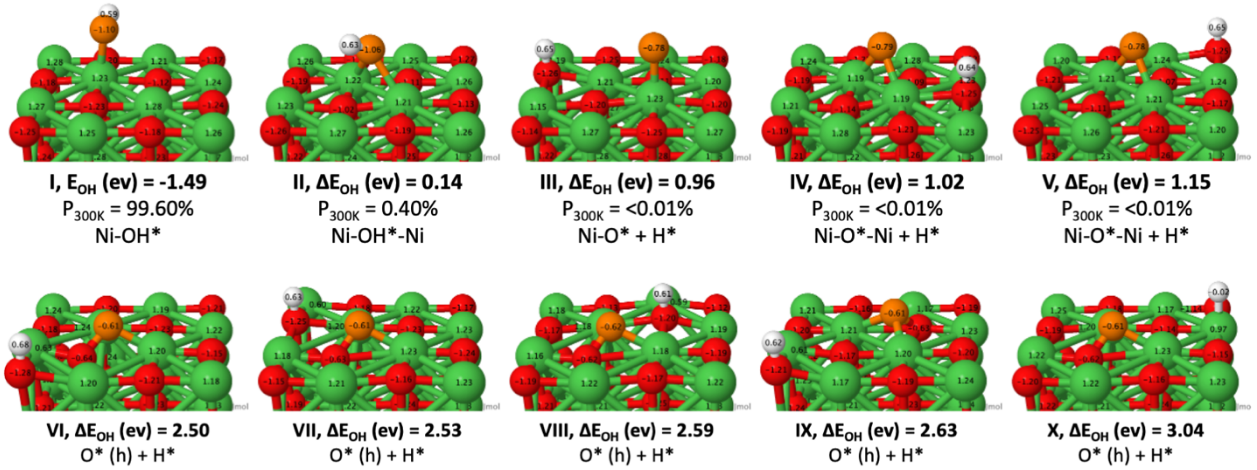

Figure 12. Lowest minima of adsorbed OH* on the rock-salt NiO (100) surface with adsorbed energy of the global minimum structure (E_{ads}), relative energies of local minima with respect to the global minimum structure (E_{ads}), and Boltzmann populations at 300 K. Bader charges are shown on atoms. Nickel atoms are in green, oxygen atoms belonging to the NiO surface are in red, oxygen atoms belonging to the adsorbed species are in orange, and hydrogen atoms are in white.

Table 4. OH* Adsorption on NiO

Isomer	ΔE_{OH} (eV)	Species	r_{O-H} (Å)	$r_{surf-ads}$ (Å)	q_{Ni} (e)	$q_{O,surf}$ (e)	$q_{OH(ads)}$
I	0.00	OH*	0.98	1.95	1.20-1.29	-1.27 to -1.12	-0.50
II	0.14	Ni-OH*-Ni	0.98	2.18-2.29, (Ni-O)<br>0.98, (O_{surf}-H)	1.21-1.29	-1.27 to -1.02	-0.43
III	0.96	O* + H*	3.25	2.02-2.07, (Ni-O)<br>0.98, (O_{surf}-H)	1.19-1.28	-1.28 to -1.09	-0.15
IV	1.02	Ni-O*-Ni + H*	4.73	1.88, (Ni-O)<br>0.98, (O_{surf}-H)	1.15-1.28	-1.28 to -1.11	-0.13

V	1.15	Ni-O*-Ni + H*	4.20	2.05, (Ni-O)<br>0.98, (O_{surf}-H)	1.19-1.27	-1.28 to -1.07	-0.13
VI	2.50	O* (h) + H*	3.72	1.93-1.96, (Ni-O)<br>0.98, (O_{surf}-H)<br>1.49, (O_{surf}-O)	0.63-1.29	-1.28 to -0.64	0.07
VII	2.53	O* (h) + H*	3.01	1.95-1.97, (Ni-O)<br>0.98, (O_{surf}-H)<br>1.47, (O_{surf}-O)	0.60-1.27	-1.27 to -0.63	0.03
VIII	2.59	O* (h) + H*	2.49	1.95-1.97, (Ni-O)<br>0.98, (O_{surf}-H)<br>1.47, (O_{surf}-O)	0.59-1.28	-1.28 to -0.62	-0.01
IX	2.63	O* (h) + H*	5.30	1.95-1.97, (Ni-O)<br>0.98, (O_{surf}-H)<br>1.47, (O_{surf}-O)	0.61-1.27	-1.28 to -0.63	0.01
X	3.04	O* (h) + H*	1.11	1.95-1.96, (Ni-O)<br>1.45, (Ni_{surf}-H)<br>1.47, (O_{surf}-O)	0.97-1.28	-1.28 to -0.62	-0.63

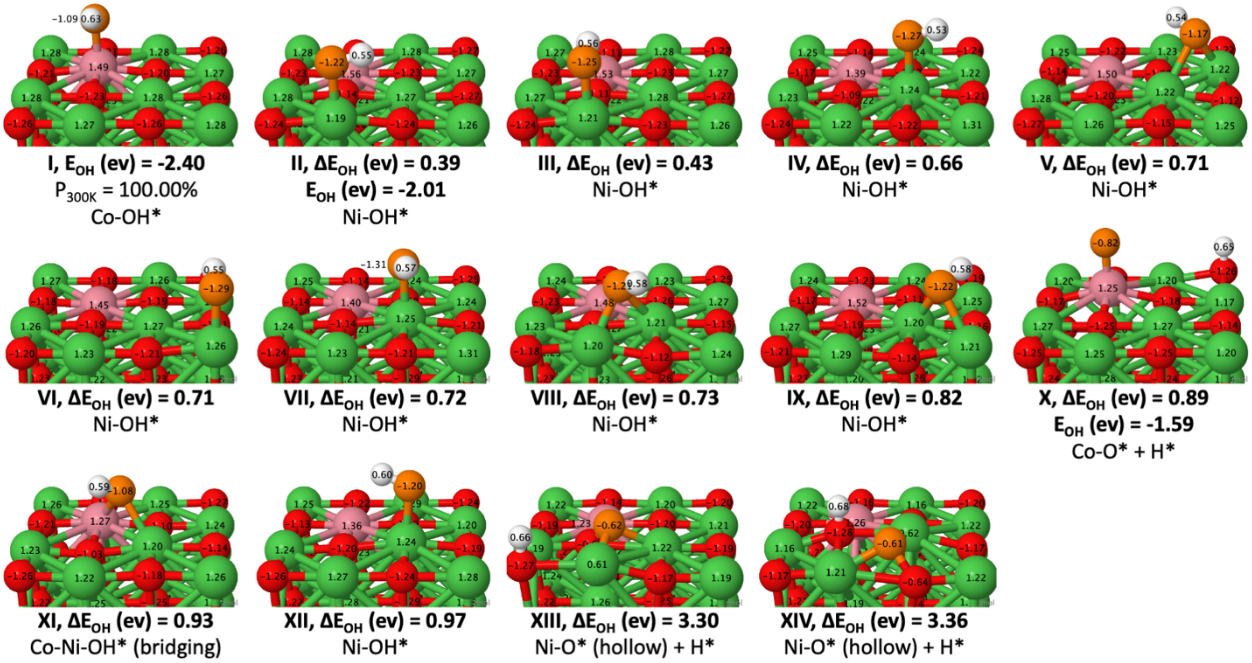

Figure 13. Lowest minima of adsorbed OH* on the rock-salt Co_{sub}-NiO (100) surface with adsorbed energy of the global minimum structure (E_{ads}), relative energies of local minima with respect to the global minimum structure (E_{ads}), and Boltzmann populations at 300 K. Bader charges are shown on atoms. Cobalt atoms are in pink, nickel atoms are in green, oxygen atoms belonging to the Co_{sub}-NiO surface are in red, oxygen atoms belonging to the adsorbed species are in orange, and hydrogen atoms are in white.

Table 5. OH* Adsorption on Co_{sub}-NiO

Isomer	ΔE_{OH} (eV)	Species	$r_{O\text{-}H}$ (Å)	$r_{surf\text{-}ads}$ (Å)	q_{Co} (e)	q_{Ni} (e)	$q_{O,surf}$ (e)	$q_{OH(ads)}$
I	0.00	Co-OH*	0.98	1.85, (Co-O)	1.49	1.22-1.28	-1.28 to -1.20	-0.46
II	0.39	Ni-OH*	0.97	1.92, (Ni-O)	1.56	1.19-1.29	-1.29 to -1.11	-0.67
III	0.43	Ni-OH*	0.97	1.93, (Ni-O)	1.53	1.20-1.28	-1.29 to -1.11	-0.70
IV	0.66	Ni-OH*	0.97	1.94, (Ni-O)	1.39	1.21-1.31	-1.29 to -1.07	-0.73
V	0.71	Ni-OH*	0.97	2.06-2.25, (Ni-O)	1.50	1.20-1.28	-1.27 to -1.09	-0.63
VI	0.71	Ni-OH*	0.97	1.94, (Ni-O)	1.45	1.21-1.28	-1.29 to -1.12	-0.75
VII	0.72	Ni-OH*	0.97	1.94, (Ni-O)	1.40	1.21-1.31	-1.29 to -1.08	-0.74
VIII	0.73	Ni-OH*	0.97	2.09-2.21, (Ni-O)	1.48	1.20-1.27	-1.28 to -1.10	-0.63
IX	0.82	Ni-OH*	0.97	2.13-2.15, (Ni-O)	1.52	1.20-1.29	-1.27 to -1.11	-0.64
X	0.89	Co-O* + H*	4.76	1.69, (Co-O)	1.25	1.17-1.28	-1.28 to -1.13	-0.17
XI	0.93	Ni-OH*-Co	0.98	2.16, (Co-O) 2.18, (Ni-O)	1.27	1.20-1.27	-1.28 to -1.03	-0.48
XII	0.97	Ni-OH*	4.71	1.94, (Ni-O)	1.36	1.20-1.29	-1.29 to -1.10	-0.59
XIII	3.30	Ni-O* + H*	3.08	1.51, (O_{surf}-O), 1.93-1.99, (Ni-O) 0.98, (O_{surf}-H)	1.23	0.61-1.28	-1.27 to -0.68	0.04
XIV	3.36	Ni-O* + H*	2.58	1.48, (O_{surf}-O),	1.26	0.62-1.28	-1.28 to -0.64	0.07

				1.95-1.96, (Ni-O) 0.98, (O_{surf}-H)				

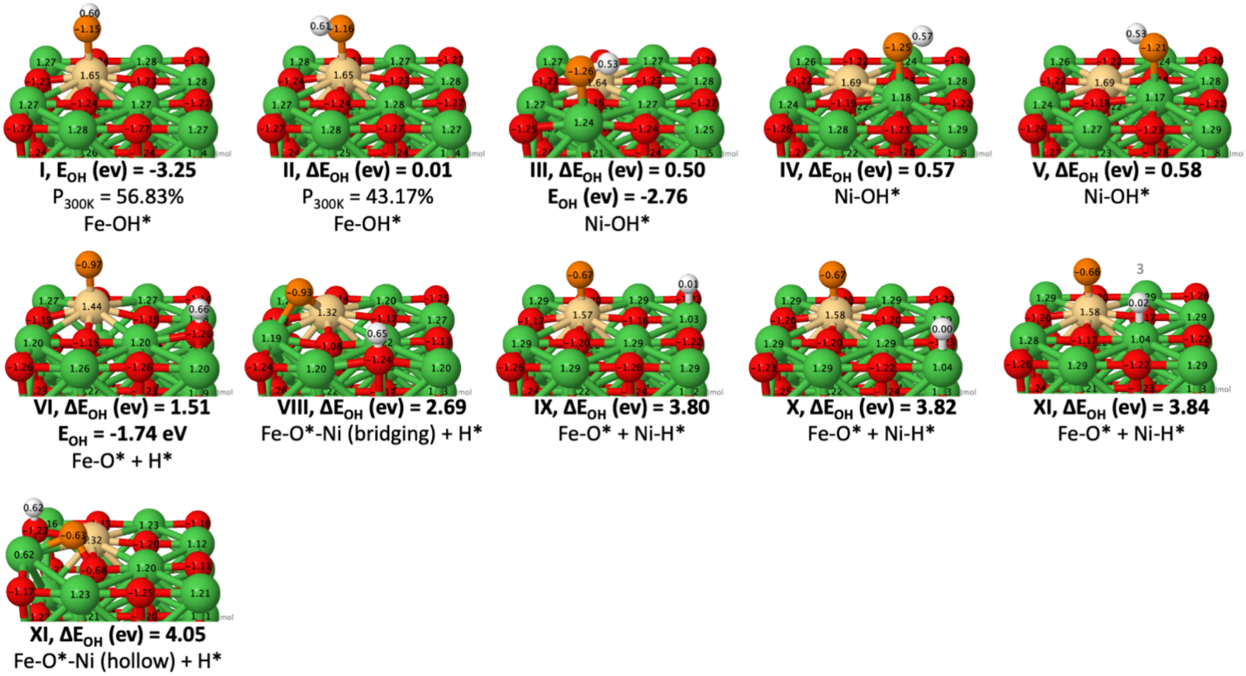

Figure 14. Lowest minima of adsorbed OH* on the rock-salt Fe_{sub}-NiO (100) surface with adsorbed energy of the global minimum structure (E_{ads}), relative energies of local minima with respect to the global minimum structure (E_{ads}), and Boltzmann populations at 300 K. Bader charges are shown on atoms. Iron atoms are in yellow, nickel atoms are in green, oxygen atoms belonging to the Fe_{sub}-NiO surface are in red, oxygen atoms belonging to the adsorbed species are in orange, and hydrogen atoms are in white.

Table 6. OH* Adsorption on Fe_{sub}-NiO

Isomer	ΔE_{OH} (eV)	Species	r_{O-H} (Å)	$r_{surf-ads}$ (Å)	q_{Fe} (e)	q_{Ni} (e)	$q_{O,surf}$ (e)	$q_{OH(ads)}$
I	0.00	Fe-OH*	0.97	1.91	1.65	1.23-1.28	-1.29 to -1.22	-0.55
II	0.01	Fe-OH*	0.97	1.91	1.65	1.24-1.28	-1.29 to -1.23	-0.55
III	0.50	Ni-OH*	0.97	1.94	1.64	1.21-1.29	-1.30 to -1.16	-0.73
IV	0.57	Ni-OH*	0.97	1.94	1.69	1.18-1.29	-1.28 to -1.18	-0.68

V	0.58	Ni-OH*	0.97	1.94	1.69	1.17-1.29	-1.28 to -1.18	-0.68
VI	1.51	Fe-O* + H*	4.76	1.74, (Fe-O) 0.98, (O_{surf}-H)	1.44	1.18-1.28	-1.30 to -1.14	-0.31
VII	2.69	Ni-O*-Fe + H*	4.17	2.01, (Fe-O) 2.02, (Ni-O) 0.98, (O_{surf}-H)	1.32	1.19-1.27	-1.27 to -1.08	-0.28
VIII	3.80	Fe-O* + H*	4.17	1.66, (Fe-O) 1.45, (Ni_{surf}-H)	1.57	1.03-1.29	-1.26 to -1.16	-0.67
IX	3.82	Fe-O* + H*	5.91	1.66, (Fe-O) 1.46, (Ni_{surf}-H)	1.58	1.04-1.29	-1.26 to -1.20	-0.67
X	3.84	Fe-O* + H*	3.00	1.66, (Fe-O) 1.46, (Ni_{surf}-H)	1.58	1.04-1.29	-1.26 to -1.17	-0.63
XI	4.05	Fe-O* (h) + H*	2.61	2.02, (Fe-O) 1.96, (Ni-O) 1.52, (O_{surf}-O) 2.47, (Ni_{surf}-H)	1.32	0.62-1.29	-1.27 to -0.68	-0.01

EDS

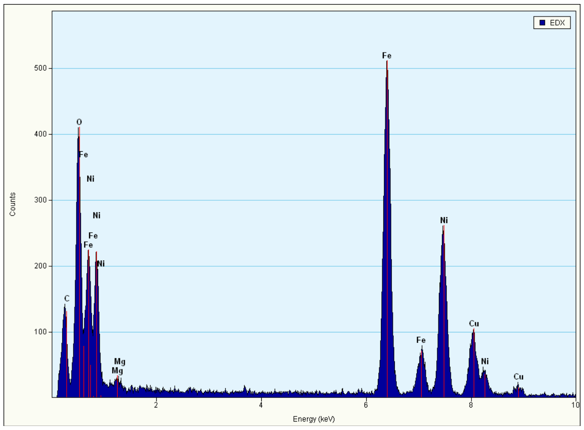

Element	Weight %	Atomic %	Uncert. %	Correction	k-Factor
-------	--------	--------	---------	----------	--------
O(K)	27.78	57.73	0.40	0.49	2.008
Fe(K)	46.84	27.88	0.34	0.99	1.359
Ni(K)	25.37	14.37	0.28	0.99	1.456

TED

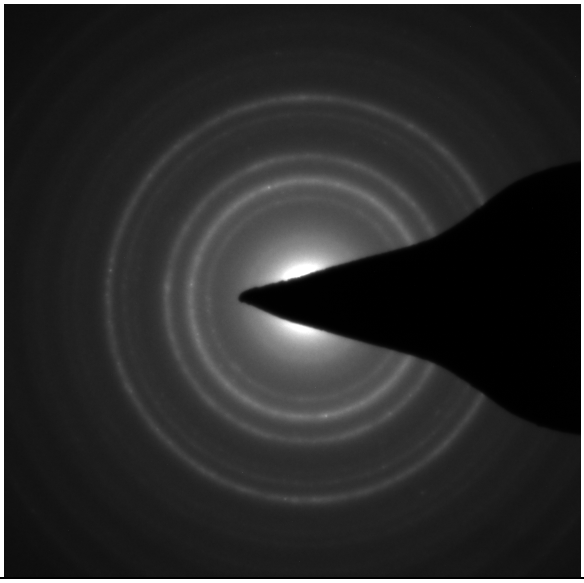

Figure 15. EDS and TED data of $NiFe_2O_4$.

a) O* on NiO

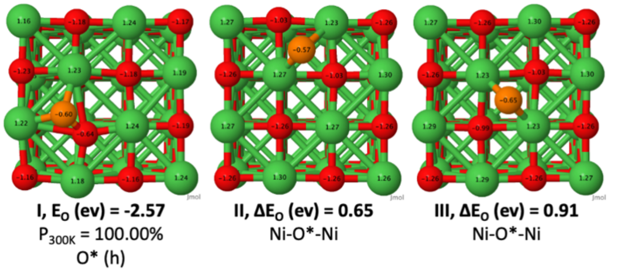

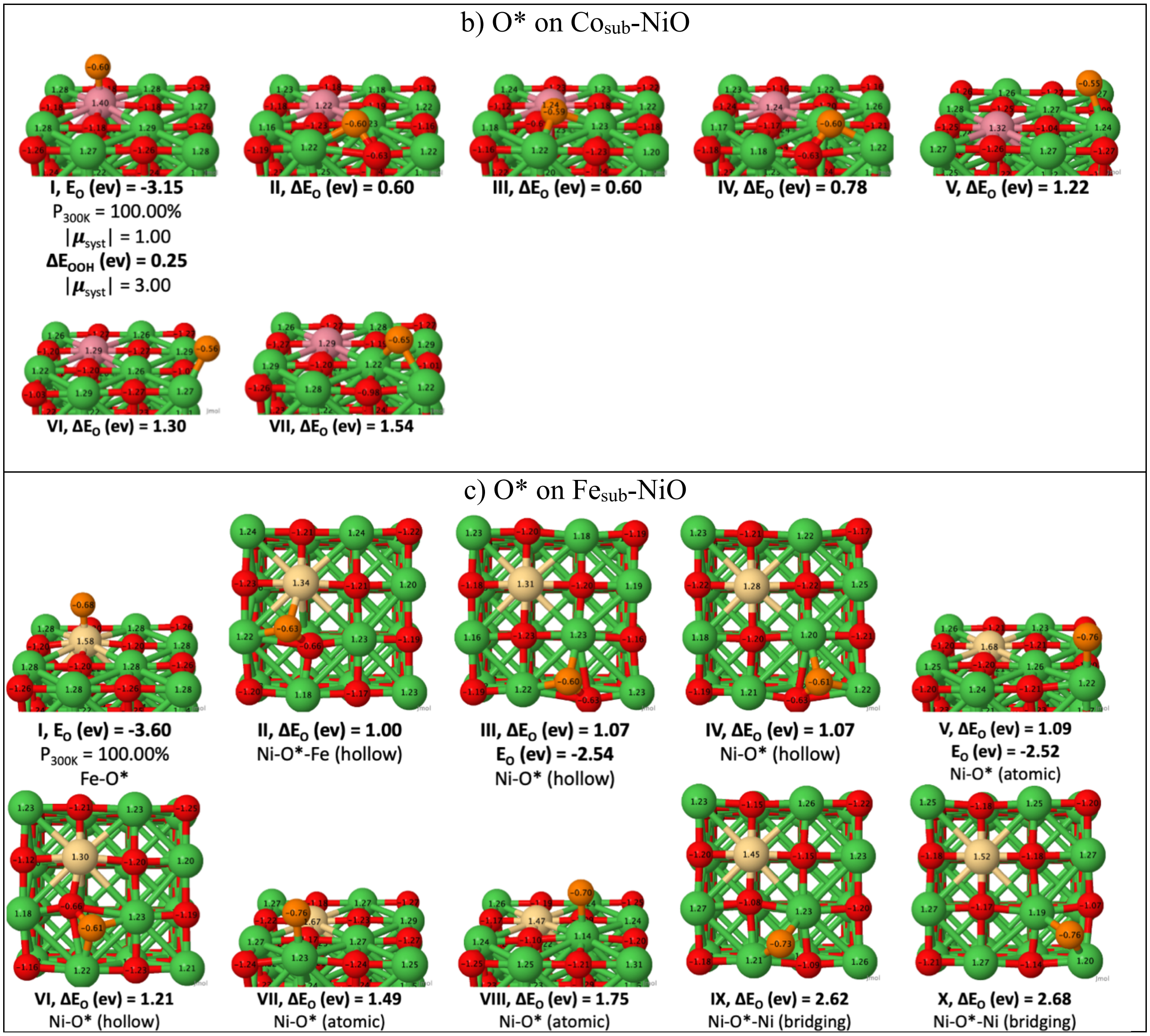

Figure 16. Lowest minima of adsorbed O* on the rock-salt NiO (100), Co$_{sub}$-NiO (100), Fe$_{sub}$-NiO (100) surfaces with adsorbed energy of the global minimum structure (E_{ads}), relative energies of local minima with respect to the global minimum structure (E_{ads}), and Boltzmann populations at 300 K. Bader charges are shown on atoms. Cobalt atoms are in pink, iron atoms are in yellow, nickel atoms are in green, oxygen atoms belonging to the surface are in red, oxygen atoms belonging to the adsorbed species are in orange, and hydrogen atoms are in white.

Table 7. O* Adsorption on NiO

Isomer	ΔE_O (eV)	$r_{surf-ads}$ (Å)	q_{Ni} (e)	$q_{O,surf}$ (e)	$q_{O(ads)}$
I	0.00	1.95-1.96, (Ni-O)<br>1.47, (O_{surf}-O)	1.16-1.26	-1.27 to -0.64	-0.60
II	0.65	1.95-2.27, (Ni-O)<br>2.27, (O_{surf}-O)	1.22-1.30	-1.27 to -1.03	-0.57
III	0.91	2.05, (Ni-O)	1.20-1.30	-1.26 to -0.99	-0.65

		2.24-2.27, (O_{surf}-O)				

Table 8. O* Adsorption on Co_{sub}-NiO

Isomer	ΔE_O (eV)	$r_{surf\text{-}ads}$ (Å)	q_{Co} (e)	q_{Ni} (e)	$q_{O,surf}$ (e)	$q_{O(ads)}$
I	0.00	1.62, (Co-O)	1.40	1.21-1.29	-1.26 to -1.18	-0.60
II	0.60	1.96, (Ni-O)<br>1.47, (O_{surf}-O)	1.22	1.16-1.26	-1.28 to -0.63	-0.60
III	0.60	1.95-1.96, (Ni-O)<br>1.47, (O_{surf}-O)	1.24	1.18-1.27	-1.27 to -0.67	-0.59
IV	0.78	1.96, (Ni-O)<br>1.47, (O_{surf}-O)	1.24	1.17-1.26	-1.27 to -0.63	-0.60
V	1.22	1.85, (Ni-O)<br>2.38-2.46, (O_{surf}-O)	1.32	1.22-1.28	-1.27 to -1.04	-0.55
VI	1.30	1.95, (Ni-O)<br>2.27, (O_{surf}-O)	1.29	1.21-1.29	-1.27 to -1.03	-0.56
VII	1.54	2.05, (Ni-O)<br>2.24-2.28, (O_{surf}-O)	1.29	1.20-1.29	-1.27 to -0.98	-0.65

Table 9. O* Adsorption on Fe_{sub}-NiO

Isomer	ΔE_O (eV)	$r_{surf\text{-}ads}$ (Å)	q_{Fe} (e)	q_{Ni} (e)	$q_{O,surf}$ (e)	$q_{O(ads)}$
I	0.00	1.66, (Fe-O)	1.58	1.22-1.28	-1.26 to -1.20	-0.68
II	1.00	2.02, (Fe-O)<br>1.99, (Ni-O)<br>1.49, (O_{surf}-O)	1.34	1.18-1.26	-1.28 to -0.66	-0.63
III	1.07	1.95-1.96, (Ni-O)<br>1.47, (O_{surf}-O)	1.31	1.16-1.28	-1.28 to -0.63	-0.60
IV	1.07	1.95-1.96, (Ni-O)<br>1.47, (O_{surf}-O)	1.28	1.18-1.28	-1.27 to -0.63	-0.61
V	1.09	1.87, (Ni-O)	1.68	1.21-1.28	-1.29 to -1.20	-0.76
VI	1.21	1.95-1.96, (Ni-O)<br>1.47, (O_{surf}-O)	1.30	1.18-1.28	-1.28 to -0.66	-0.61
VII	1.49	1.87, (Ni-O)	1.67	1.22-1.28	-1.30 to -1.17	-0.76
VIII	1.75	1.87, (Ni-O)	1.47	1.14-1.31	-1.29 to -1.09	-0.70
IX	2.62	1.99-2.09, (Ni-O)<br>2.27, (O_{surf}-O)	1.45	1.20-1.29	-1.29 to -1.08	-0.73
X	2.67	2.03-2.05, (Ni-O)<br>2.27, (O_{surf}-O)	1.52	1.19-1.27	-1.27 to -1.07	-0.76

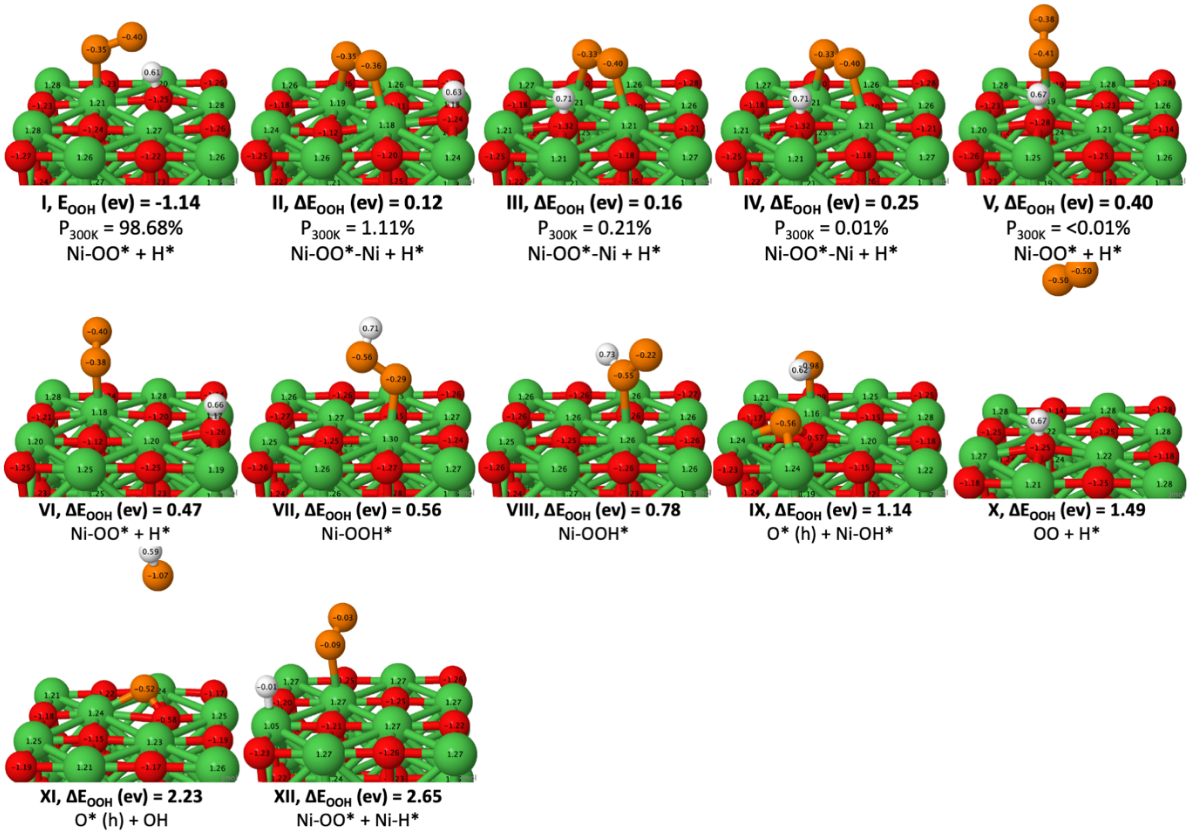

Figure 17. Lowest minima of adsorbed OOH* on the rock-salt NiO (100) surface with adsorbed energy of the global minimum structure (E_{ads}), relative energies of local minima with respect to the global minimum structure (E_{ads}), and Boltzmann populations at 300 K. Bader charges are shown on atoms. Nickel atoms are in green, oxygen atoms belonging to the NiO surface are in red, oxygen atoms belonging to the adsorbed species are in orange, and hydrogen atoms are in white.

Table 10. OOH* Adsorption on NiO

Isomer	ΔE_{OOH} (eV)	Species	r_{O-O} (Å)	r_{O-H} (Å)	$r_{surf-ads}$ (Å)	q_{Ni} (e)	$q_{O,surf}$ (e)	$q_{OOH(ads)}$
I	0.00	Ni-OO* + H*	1.35	1.48-2.13	2.04, (Ni-O) 1.06, (O_{surf}-H)	1.20-1.28	-1.27 to -1.22	-0.14
II	0.20	Ni-OO*-Ni + H*	1.35	2.90-4.06	2.09 (Ni-O) 0.98, (O_{surf}-H)	1.18-1.27	-1.28 to -1.11	-0.07
III	0.34	Ni-OO*-Ni + H*	1.35	4.55	2.10, (Ni-O) 0.98, (O_{surf}-H)	1.17-1.28	-1.28 to -1.12	-0.06

IV	0.52	Ni-OO*-Ni + H*	1.36	2.02-2.04	2.17-2.19, (Ni-O) 0.98, (O_{surf}-H)	1.20-1.27	-1.32 to -1.10	-0.03
V	0.55	Ni-OO* + H*	1.34	1.94-3.23	1.99-2.41, (Ni-O) 0.99, (O_{surf}-H)	1.19-1.28	-1.28 to -1.14	-0.11
VI	0.78	Ni-OO* + H*	1.35	4.66-5.41	2.00, (Ni-O) 0.99, (O_{surf}-H)	1.17-1.28	-1.28 to -1.12	-0.11
VII	1.00	Ni-OOH*	1.38	0.99	2.05, (Ni-O)	1.24-1.30	-1.27 to -1.15	-0.14
VIII	1.24	Ni-OOH*	1.35	0.99	2.32, (Ni-O)	1.24-1.27	-1.27 to -1.23	-0.05
IX	1.31	O*(h) + Ni-OH*	2.92	1.75-3.02	1.85, (Ni-OH) 1.97-1.99, (Ni-O) 1.45, (O_{surf}-O)	1.16-1.28	-1.28 to -0.57	-0.92
X	1.62	OO + H*	1.36	4.62-5.07	0.98, (O_{surf}-H)	1.19-1.28	-1.28 to -1.14	-0.33
XI	2.21	O*(h) + OH	4.22	0.98	1.96-1.98, (Ni-O)	1.21-1.26	-1.27 to -0.58	-0.99
XII	2.32	OO + Ni-H*	1.25	2.90-4.05	1.46, (Ni_{surf}-H)	1.05-1.27	-1.27 to -1.20	-0.13

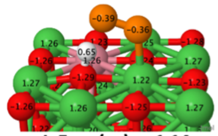

I, E_{OOH} (ev) = -1.14
P_{300K} = 23.77%
$Ni-O_2^*$ + H*

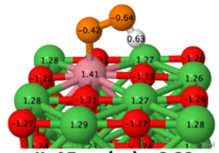

II, ΔE_{OOH} (ev) = 0.00
E_{OOH} (ev) = -1.14
P_{300K} = 22.75%
Co-OOH*

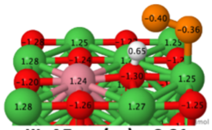

III, ΔE_{OOH} (ev) = 0.01
P_{300K} = 13.84%
$Ni-O_2^*$ + H*

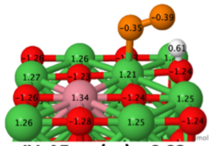

IV, ΔE_{OOH} (ev) = 0.02
P_{300K} = 13.09%
$Ni-O_2^*$ + H*

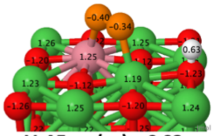

V, ΔE_{OOH} (ev) = 0.02
P_{300K} = 10.46%
$Co-O_2^*$-Ni + H*

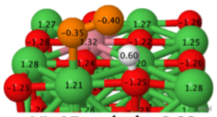

VI, ΔE_{OOH} (ev) = 0.02
P_{300K} = 10.01%
$Ni-O_2^*$ + H*

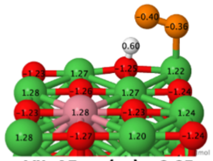

VII, ΔE_{OOH} (ev) = 0.05
P_{300K} = 3.38%
$Ni-O_2^*$ + H*

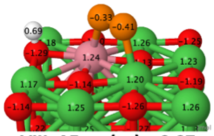

VIII, ΔE_{OOH} (ev) = 0.07
P_{300K} = 1.37%
$Co-O_2^*$-Ni + H*

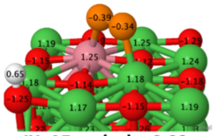

IX, ΔE_{OOH} (ev) = 0.09
P_{300K} = 0.69%
$Co-O_2^*$-Ni + H*

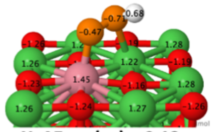

X, ΔE_{OOH} (ev) = 0.12
E_{OOH} (ev) = -1.02
P_{300K} = 0.26%
Co-OOH*-Ni

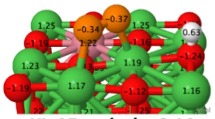

XI, ΔE_{OOH} (ev) = 0.14
P_{300K} = 0.13%
$Ni-O_2^*$-Ni + H*

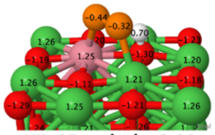

XII, ΔE_{OOH} (ev) = 0.14
P_{300K} = 0.11%
$Co-O_2^*$-Ni + H*

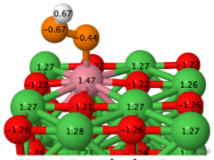

XIII, ΔE_{OOH} (ev) = 0.15
P_{300K} = 0.07%
Co-OOH*

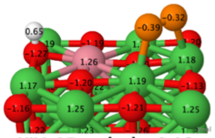

XIV, ΔE_{OOH} (ev) = 0.16
P_{300K} = 0.05%
$Ni-O_2^*$-Ni + H*

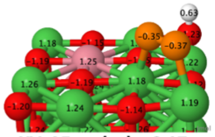

XV, ΔE_{OOH} (ev) = 0.17
P_{300K} = 0.04%
$Ni-O_2^*$-Ni + H*

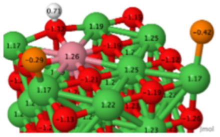

XVI, ΔE_{OOH} (ev) = 0.24
$Ni-O_2^*$-Ni + H*

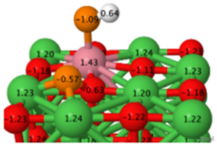

XVII, ΔE_{OOH} (ev) = 0.42
Ni-O*(h) + Co-OH*

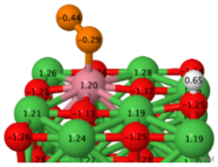

XVIII, ΔE_{OOH} (ev) = 0.42
$Co-O_2^*$ + H*

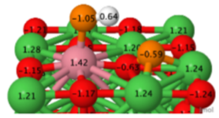

XIX, ΔE_{OOH} (ev) = 0.43
Ni-O*(h) + Co-OH*

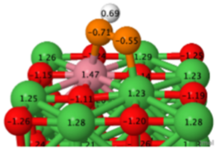

XX, ΔE_{OOH} (ev) = 0.43
Ni-OOH*-Co

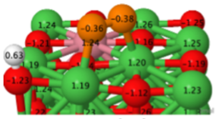

XXI, ΔE_{OOH} (ev) = 0.51
$Ni-O_2^*$-Ni + H*

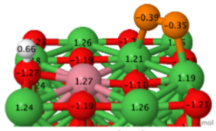

XXII, ΔE_{OOH} (ev) = 0.52
$Ni-O_2^*$-Ni + H*

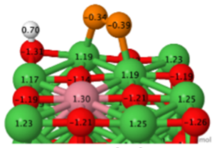

XXIII, ΔE_{OOH} (ev) = 0.55
$Ni-O_2^*$-Ni + H*

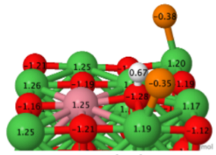

XXIV, ΔE_{OOH} (ev) = 0.55
$Ni-O_2^*$-Ni + H*

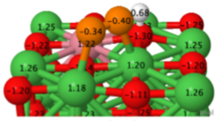

XXV, ΔE_{OOH} (ev) = 0.55
$Ni-O_2^*$-Ni + H*

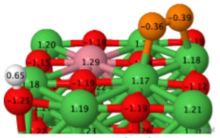

XXVI, ΔE_{OOH} (ev) = 0.56
$Ni-O_2^*$-Ni + H*

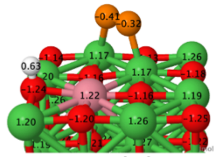

XXVII, ΔE_{OOH} (ev) = 0.58
$Ni-O_2^*$-Ni + H*

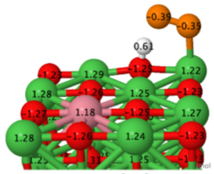

XXVIII, ΔE_{OOH} (ev) = 0.77
$Ni-O_2^*$-Ni + H*

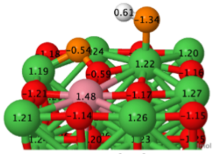

XXIX, ΔE_{OOH} (ev) = 1.02
Ni-O*(h) + Ni-OH*

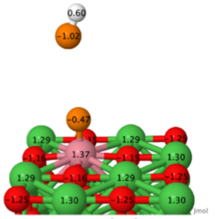

XXX, ΔE_{OOH} (ev) = 1.81
Co-O* + OH

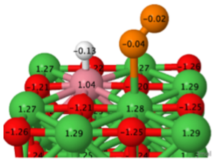

XXXI, ΔE_{OOH} (ev) = 2.46
Ni-OO* + Co-H*

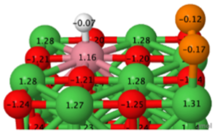

XXXII, ΔE_{OOH} (ev) = 2.51
Ni-OO* + Co-H*

Figure 18. Lowest minima of adsorbed OOH* on the rock-salt Co_{sub}-NiO (100) surface with adsorbed energy of the global minimum structure (E_{ads}), relative energies of local minima with respect to the global minimum structure (E_{ads}), and Boltzmann populations at 300 K. Bader charges are shown on atoms. Cobalt atoms are in pink, nickel atoms are in green, oxygen atoms belonging to the Co_{sub}-NiO surface are in red, oxygen atoms belonging to the adsorbed species are in orange, and hydrogen atoms are in white.

Table 11. OOH* Adsorption on Co_{sub}-NiO

Isomer	ΔE_{OOH} (eV)	Species	r_{O-O} (Å)	r_{O-H} (Å)	$r_{surf-ads}$ (Å)	q_{Co} (e)	q_{Ni} (e)	$q_{O,surf}$ (e)	$q_{OOH(ads)}$
I	0.00	Ni-OO* + H*	1.35	1.48-2.14	2.04, (Ni-O) 1.06, (O_{surf}-H)	1.26	1.20-1.27	-1.29 to -1.22	-0.10
II	0.00	Co-OOH*	1.43	1.02	1.90, (Co-O) 1.30, (O_{surf}-H)	1.41	1.23-1.29	-1.27 to -1.19	-0.43
III	0.01	Ni-OO* + H*	1.35	1.48-2.12	2.05, (Ni-O) 1.06, (O_{surf}-H)	1.24	1.20-1.28	-1.30 to -1.20	-0.11
IV	0.02	Ni-OO* + H*	1.35	1.48-2.14	2.04, (Ni-O) 1.06, (O_{surf}-H)	1.34	1.20-1.28	-1.28 to -1.22	-0.14
V	0.02	Co-OO*-Ni + H*	1.36	2.83-3.98	2.07, (Co-O) 2.06, (Ni-O) 0.98, (O_{surf}-H)	1.25	1.16-1.27	-1.28 to -1.12	-0.11
VI	0.02	Ni-OO* + H*	1.35	1.47-2.13	2.04, (Ni-O) 1.06, (O_{surf}-H)	1.32	1.20-1.29	-1.28 to -1.22	-0.15
VII	0.05	Ni-OO* + H*	1.35	1.47-2.13	2.06, (Ni-O) 1.06, (O_{surf}-H)	1.28	1.20-1.28	-1.27 to -1.22	-0.15
VIII	0.07	Co-OO*-Ni + H*	1.36	3.01-4.16	2.07, (Co-O) 2.07, (Ni-O) 0.98, (O_{surf}-H)	1.24	1.17-1.28	-1.29 to -1.13	-0.05
IX	0.09	Co-OO*-Ni + H*	1.36	4.54-4.56	2.07, (Co-O) 2.07, (Ni-O) 0.98, (O_{surf}-H)	1.25	1.17-1.27	-1.28 to -1.12	-0.08

X	0.12	Co-OOH*-Ni	1.47	0.98	1.92, (Co-O) 2.35, (Ni-O)	1.45	1.20-1.28	-1.26 to -1.11	-0.50
XI	0.14	Ni-OO*-Ni + H*	1.35	2.90-4.06	2.10, (Ni-O) 0.98, (O_{surf}-H)	1.22	1.16-1.26	-1.28 to -1.12	-0.08
XII	0.14	Co-OO*-Ni + H*	1.36	2.02-2.03	2.10, (Co-O) 2.12, (Ni-O) 0.98, (O_{surf}-H)	1.25	1.20-1.27	-1.30 to -1.11	-0.06
XIII	0.15	Co-OOH*	1.44	0.98	1.89, (Co-O)	1.47	1.23-1.28	-1.29 to -1.21	-0.43
XIV	0.16	Ni-OO*-Ni + H*	1.35	4.98-5.70	2.09-2.12, (Ni-O) 0.98, (O_{surf}-H)	1.26	1.17-1.26	-1.28 to -1.10	-0.06
XV	0.17	Ni-OO*-Ni + H*	1.35	4.95-5.67	2.11, (Ni-O) 0.98, (O_{surf}-H)	1.25	1.17-1.26	-1.28 to -1.12	-0.08
XVI	0.24	Ni-OO*-Ni + H*	1.35	5.51-6.81	2.09-2.12, (Ni-O) 0.98, (O_{surf}-H)	1.26	1.17-1.27	-1.32 to -1.12	0.01
XVII	0.42	Ni-O* + Co-OH*	3.06	0.97	1.87, (Co-OH_{ads}) 1.94, (Ni-O_{ads}) 1.46, (O_{surf}-O_{ads})	1.43	1.18-1.28	-1.28 to -0.62	-1.01
XVIII	0.42	Co-OO* + H*	1.35	4.61-5.87	2.00, (Co-O) 0.98, (O_{surf}-H)	1.20	1.16-1.28	-1.28 to -1.13	-0.08
XIX	0.43	Ni-O* + Co-OH*	2.89	0.98	1.84, (Co-O) 1.95-1.96, (Ni-O) 1.47, (O_{surf}-O)	1.42	1.20-1.28	-1.27 to -0.63	-0.99
XX	0.43	Ni-OOH*-Co	1.48	0.98	2.15, (Co-O) 2.05, (Ni-O)	1.47	1.21-1.29	-1.27 to -1.11	-0.58
XXI	0.51	Ni-OO*-Ni + H*	1.35	2.88-4.07	2.09-2.11, (Ni-O)	1.24	1.19-1.27	-1.28 to -1.12	-0.11

					0.98, (O_{surf}-H)				
XXII	0.52	Ni-OO*-Ni + H*	1.35	4.95-5.74	2.10, (Ni-O) 0.98, (O_{surf}-H)	1.27	1.18-1.27	-1.28 to -1.11	-0.09
XXIII	0.55	Ni-OO*-Ni + H*	1.35	2.91-4.07	2.09, (Ni-O) 0.98, (O_{surf}-H)	1.25	1.17-1.26	-1.28 to -1.12	-0.06
XXIV	0.55	Ni-OO*-Ni + H*	1.35	2.87-4.06	2.09-2.11, (Ni-O) 0.98, (O_{surf}-H)	1.28	1.19-1.26	-1.28 to -1.12	-0.11
XXV	0.56	Ni-OO*-Ni + H*	1.35	2.87-4.07	2.09-2.11, (Ni-O) 0.98, (O_{surf}-H)	1.22	1.18-1.27	-1.30 to -1.11	-0.06
XXVI	0.56	Ni-OO*-Ni + H*	1.35	5.46-6.78	2.11-2.12, (Ni-O) 0.98, (O_{surf}-H)	1.29	1.17-1.27	-1.28 to -1.12	-0.09
XXVII	0.58	Ni-OO*-Ni + H*	1.35	4.57-4.59	2.09-2.10, (Ni-O) 0.98, (O_{surf}-H)	1.22	1.17-1.27	-1.28 to -1.13	-0.09
XXVIII	0.77	Ni-OO* + H*	1.35	1.47-2.13	2.05, (Ni-O_{ads}) 1.06, (O_{surf}-H)	1.18	1.20-1.30	-1.31 to -1.21	-0.14
XXIX	1.02	Ni-OH* + Ni-O*	3.31	0.97	1.93-1.96, (Ni-O_{ads}) 1.44, (O_{surf}-O_{ads}) 1.93, (Ni-OH_{ads})	1.48	1.19-1.27	-1.28 to -0.59	-1.27
XXX	1.81	Co-O* + OH	3.66	0.98	1.62, (Co-O)	1.37	1.21-1.30	-1.26 to -1.15	-0.90
XXXI	2.46	Ni-OO* + Co-H*	1.24	3.08-3.61	2.04, (Ni-O) 1.46, (Co_{surf}-H)	1.04	1.24-1.29	-1.27 to -1.20	-0.19
XXXII	2.51	Ni-OO* + Co-H*	1.27	5.46-6.11	2.11, (Ni-O)	1.16	1.23-1.31	-1.26 to -1.20	-0.36

					1.47, (Co_{surf}-H)				

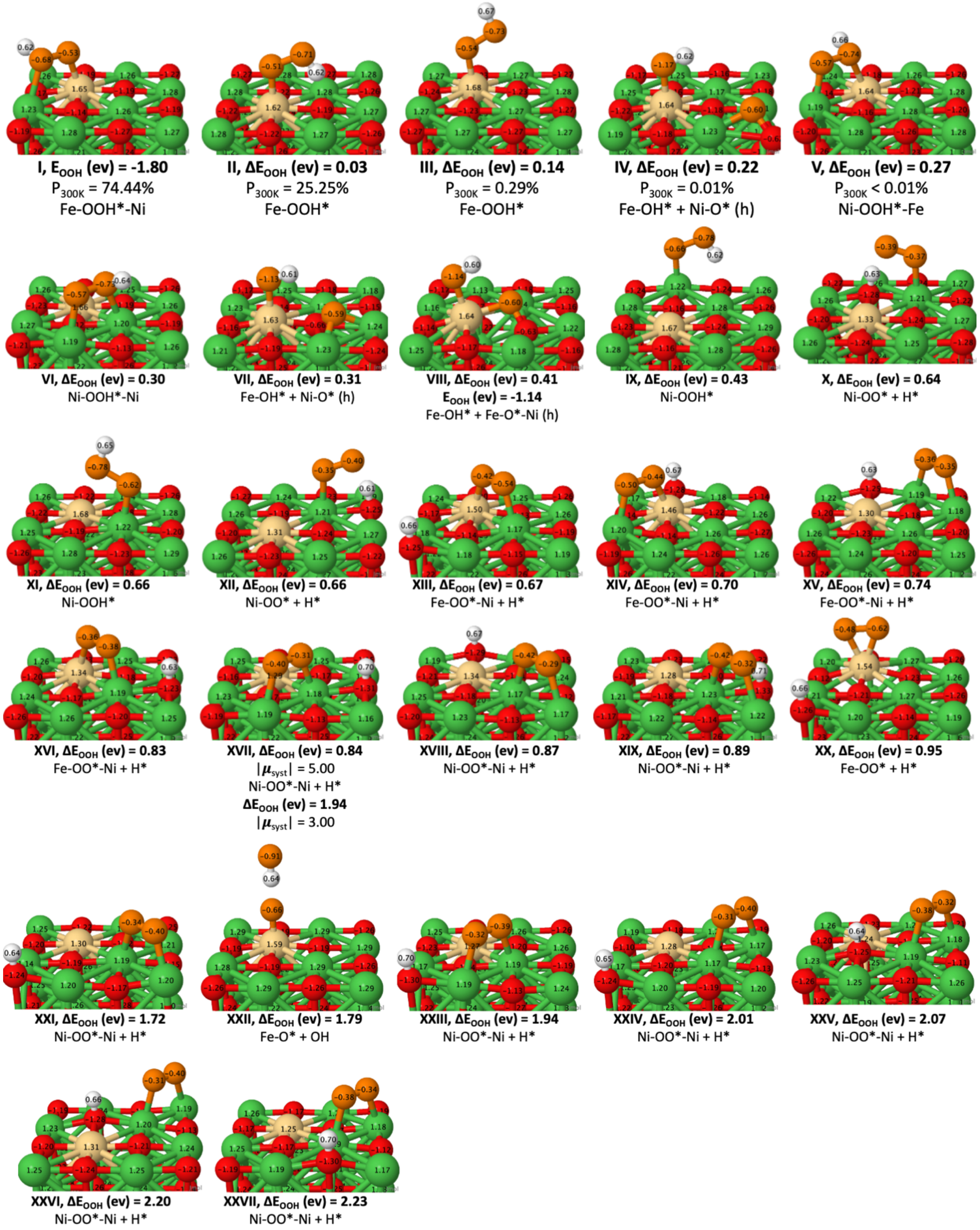

Figure 19. Lowest minima of adsorbed OOH* on the rock-salt Fe_{sub}-NiO (100) surface with adsorbed energy of the global minimum structure (E_{ads}), relative energies of local minima with

respect to the global minimum structure (E_{ads}), and Boltzmann populations at 300 K. Bader charges are shown on atoms. Iron atoms are in yellow, nickel atoms are in green, oxygen atoms belonging to the Fe_{sub}-NiO surface are in red, oxygen atoms belonging to the adsorbed species are in orange, and hydrogen atoms are in white.

Table 12. OOH* Adsorption on Fe_{sub}-NiO

Isomer	ΔE_{OOH} (eV)	Species	r_{O-O} (Å)	r_{O-H} (Å)	$r_{surf-ads}$ (Å)	q_{Fe} (e)	q_{Ni} (e)	$q_{O,surf}$ (e)	$q_{OOH(ads)}$
I	0.00	Fe-OOH*-Ni	1.48	0.98	1.99, (Fe-O)<br>2.27, (Ni-O)	1.65	1.19-1.28	-1.27 to -1.14	-0.58
II	0.03	Fe-OOH*	1.47	1.00	1.93, (Fe-O)	1.62	1.21-1.28	-1.27 to -1.19	-0.60
III	0.14	Fe-OOH*	1.47	0.98	1.93, (Fe-O)	1.68	1.23-1.28	-1.29 to -1.22	-0.59
IV	0.22	Fe-OH* + Ni-O*	4.28	0.97	2.41, (Fe-OH_{ads})<br>1.95, (Ni-O_{ads})<br>1.47, (O_{surf}-O)	1.64	1.17-1.28	-1.27 to -0.62	-1.15
V	0.27	Ni-OOH*-Fe	1.50	0.98	2.21, (Fe-O)<br>2.01, (Ni-O)	1.64	1.19-1.28	-1.27 to -1.14	-0.64
VI	0.30	Ni-OOH*-Ni	1.51	0.98	1.99-2.18, (Ni-O)	1.66	1.19-1.27	-1.28 to -1.12	-0.66
VII	0.31	Fe-OH* + Ni-O*	3.09	0.97	1.89, (Fe-OH)<br>1.95-1.96, (Ni-O)<br>1.47, (O_{surf}-O)	1.63	1.18-1.26	-1.27 to -0.66	-1.11
VIII	0.41	Fe-OH* + Ni-O*	2.77	0.98	1.91, (Fe-OH)<br>1.97, (Ni-O)<br>1.47, (O_{surf}-O)	1.64	1.13-1.26	-1.28 to -0.63	-1.14
IX	0.43	Ni-OOH*	1.50	0.99	1.95, (Ni-O)	1.67	1.21-1.29	-1.26 to -1.16	-0.82

					1.79, (O_{surf}-H)				
X	0.64	Ni-OO* + OH*	1.35	1.49-2.14	2.05, (Ni-O) 1.29, (O_{surf}-H)	1.33	1.18-1.27	-1.28 to -1.20	-0.13
XI	0.66	Ni-OOH*	1.50	0.97	1.95, (Ni-O)	1.68	1.22-1.29	-1.28 to -1.18	-0.76
XII	0.66	Ni-OO* + OH*	1.35	1.47-2.13	2.04, (Ni-O) 1.06, (O_{surf}-H)	1.31	1.19-1.27	-1.27 to -1.19	-0.14
XIII	0.67	Fe-OO*-Ni + OH*	1.41	4.54-4.56	1.97, (Fe-O) 2.04, (Ni-O) 0.98, (O_{surf}-H)	1.50	1.17-1.26	-1.28 to -1.13	-0.30
XIV	0.70	Fe-OO*-Ni + OH*	1.35	2.92-4.09	2.09-2.10, (Ni-O) 0.98, (O_{surf}-H)	1.46	1.17-1.27	-1.28 to -1.14	-0.27
XV	0.74	Ni-OO*-Ni + OH*	1.35	2.92-4.09	2.09-2.10, (Ni-O) 0.98, (O_{surf}-H)	1.30	1.18-1.28	-1.28 to -1.12	-0.07
XVI	0.83	Fe-OO*-Ni + OH*	1.35	2.75-3.92	2.20, (Fe-O) 2.08, (Ni-O) 0.98, (O_{surf}-H)	1.34	1.19-1.27	-1.28 to -1.17	-0.11
XVII	0.84	Ni-OO*-Ni + OH*	1.35	2.86-4.02	2.08-2.13, (Ni-O) 0.98, (O_{surf}-H)	1.29	1.14-1.29	-1.31 to -1.13	-0.01
XVIII	0.87	Ni-OO*-Ni + OH*	1.35	5.57-6.86	2.11-2.12, (Ni-O) 0.98, (O_{surf}-H)	1.34	1.17-1.27	-1.29 to -1.12	-0.04
XIX	0.89	Ni-OO*-Ni + OH*	1.36	2.01	2.18-2.19, (Ni-O)	1.28	1.20-1.27	-1.33 to -1.14	-0.03

					0.98, (O_{surf}-H)				
XX	0.95	Fe-OO* + OH*	1.46	1.96	1.96, (Fe-O) 0.98, (O_{surf}-H)	1.54	1.19-1.27	-1.27 to -1.12	-0.45
XXI	1.72	Ni-OO*-Ni + OH*	1.37	5.02-5.76	2.05-2.07, (Ni-O) 0.98, (O_{surf}-H)	1.30	1.14-1.30	-1.28 to -1.15	-0.10
XXII	1.79	Fe-O* + OH	2.55	1.01-1.55	1.68, (Fe-O)	1.59	1.22-1.29	-1.26 to -1.19	-0.93
XXIII	1.94	Ni-OO*-Ni + OH*	1.35	0.98	2.09-2.11, (Ni-O)	1.27	1.17-1.30	-1.30 to -1.13	-0.01
XXIV	2.01	Ni-OO*-Ni + OH*	1.35	5.46-6.76	2.09-2.12, (Ni-O) 0.98, (O_{surf}-H)	1.28	1.17-1.30	-1.29 to -1.10	-0.06
XXV	2.07	Ni-OO*-Ni + OH*	1.35	2.91-4.07	2.08-2.12, (Ni-O) 0.98, (O_{surf}-H)	1.24	1.18-1.28	-1.28 to -1.11	-0.06
XXVI	2.20	Ni-OO*-Ni + OH*	1.35	2.90-4.04	2.08-2.10, (Ni-O) 0.98, (O_{surf}-H)	1.31	1.19-1.25	-1.28 to -1.12	-0.05
XXVII	2.23	Ni-OO*-Ni + OH*	1.35	2.89-4.04	2.10-2.11, (Ni-O) 0.98, (O_{surf}-H)	1.25	1.17-1.26	-1.30 to -1.12	-0.02

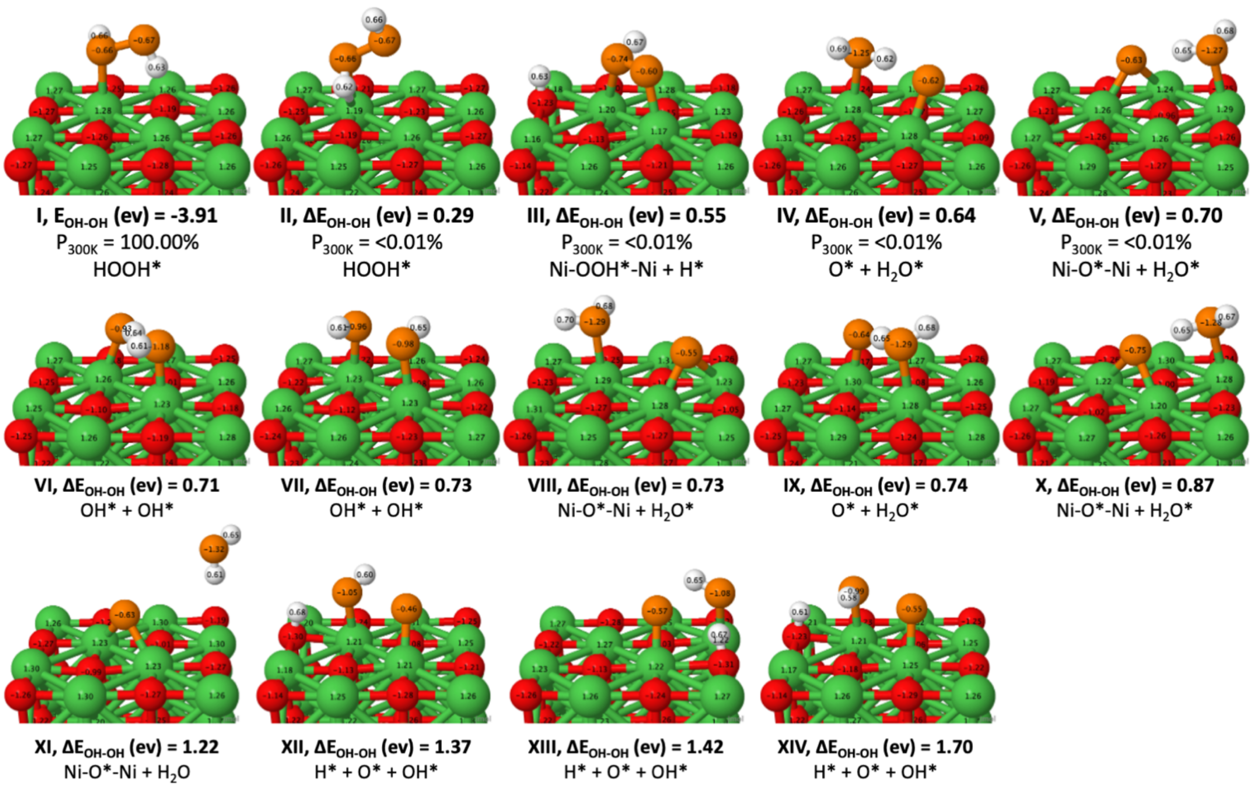

Figure 20. Lowest minima of co-adsorbed geometries resulting from OH* + OH* on the rock-salt NiO (100) surface with adsorbed energy of the global minimum structure (E_{ads}), relative energies of local minima with respect to the global minimum structure (E_{ads}), and Boltzmann populations at 300 K. Bader charges are shown on atoms. Nickel atoms are in green, oxygen atoms belonging to the NiO surface are in red, oxygen atoms belonging to the adsorbed species are in orange, and hydrogen atoms are in white.

.

Table 13. OH* + OH* Adsorption on NiO

Isomer	ΔE_{OH+OH} (eV)	Species	r_{O-O} (Å)	r_{O-H} (Å)	$r_{surf-ads}$ (Å)	q_{Ni} (e)	$q_{O,surf}$ (e)	$q_{OH+OH(ads)}$
I	0.00	HOOH*	1.48	0.98-1.04	2.17, (Ni-O) 1.55, (O_{surf}-H)	1.22-1.28	-1.28 to -1.19	-0.04
II	0.29	HOOH*	1.48	0.98-1.04	2.29, (Ni-O)	1.19-1.27	-1.27 to -1.19	-0.05
III	0.55	Ni-OOH*-Ni + H*	1.51	0.98	2.01-2.16, (Ni-OOH_{ads}) 0.98, (O_{surf}-H)	1.16-1.28	-1.28 to -1.13	-0.04
IV	0.64	O* + H_2O*	2.74	0.97-1.00	1.88, (Ni-O_{ads}) 2.11, (Ni-$O_{ads}H_2$)	1.22-1.32	-1.27 to -1.07	-0.55
V	0.70	Ni-O*-Ni + H_2O*	2.93	0.98-0.99	2.00-2.25, (Ni-O_{ads})	1.21-1.29	-1.26 to -1.08	-0.67

					2.15, (Ni-$O_{ads}H_2$)			
VI	0.71	OH* + OH*	2.61	0.97-1.03	1.93-1.96, (Ni-O_{ads}H)	1.20-1.29	-1.28 to -0.96	-0.57
VII	0.73	OH* + OH*	2.37	0.98	1.99, (Ni-O_{ads}H)	1.22-1.28	-1.26 to -1.01	-0.87
VIII	0.73	O* + H_2O*	3.44	0.98	1.93-2.35, (Ni-O_{ads}) 2.12, (Ni-$O_{ads}H_2$)	1.21-1.30	-1.27 to -1.08	-0.61
IX	0.74	O* + H_2O*	2.56	0.97-1.04	1.75, (Ni-O_{ads}) 2.07, (Ni-$O_{ads}H_2$)	1.21-1.31	-1.27 to -1.05	-0.47
X	0.87	O* + H_2O*	2.70	0.98-1.01	2.00-2.25, (Ni-O_{ads}) 2.13, (Ni-$O_{ads}H_2$)	1.20-1.30	-1.28 to -1.00	-0.70
XI	1.22	O* + H_2O	4.52	0.97-0.99	2.05-2.28, (Ni-O_{ads})	1.19-1.30	-1.27 to -0.99	-0.69
XII	1.37	H* + O* + OH*	2.43	0.97	1.89, (Ni-O_{ads}) 1.97, (Ni-O_{ads}H) 0.99, (O_{surf}-H_{ads})	1.18-1.31	-1.30 to -1.08	-0.23
XIII	1.42	H* + O* + OH*	2.38	0.98	1.88, (Ni-O_{ads}) 1.99, (Ni-O_{ads}H) 1.01, (O_{surf}-H_{ads})	1.20-1.27	-1.31 to -1.03	-0.34
XIV	1.70	H* + O* + OH*	2.39	0.98	1.90, (Ni-O_{ads}) 1.99, (Ni-O_{ads}H) 0.99, (O_{surf}-H_{ads})	1.17-1.32	-1.29 to -1.06	-0.35

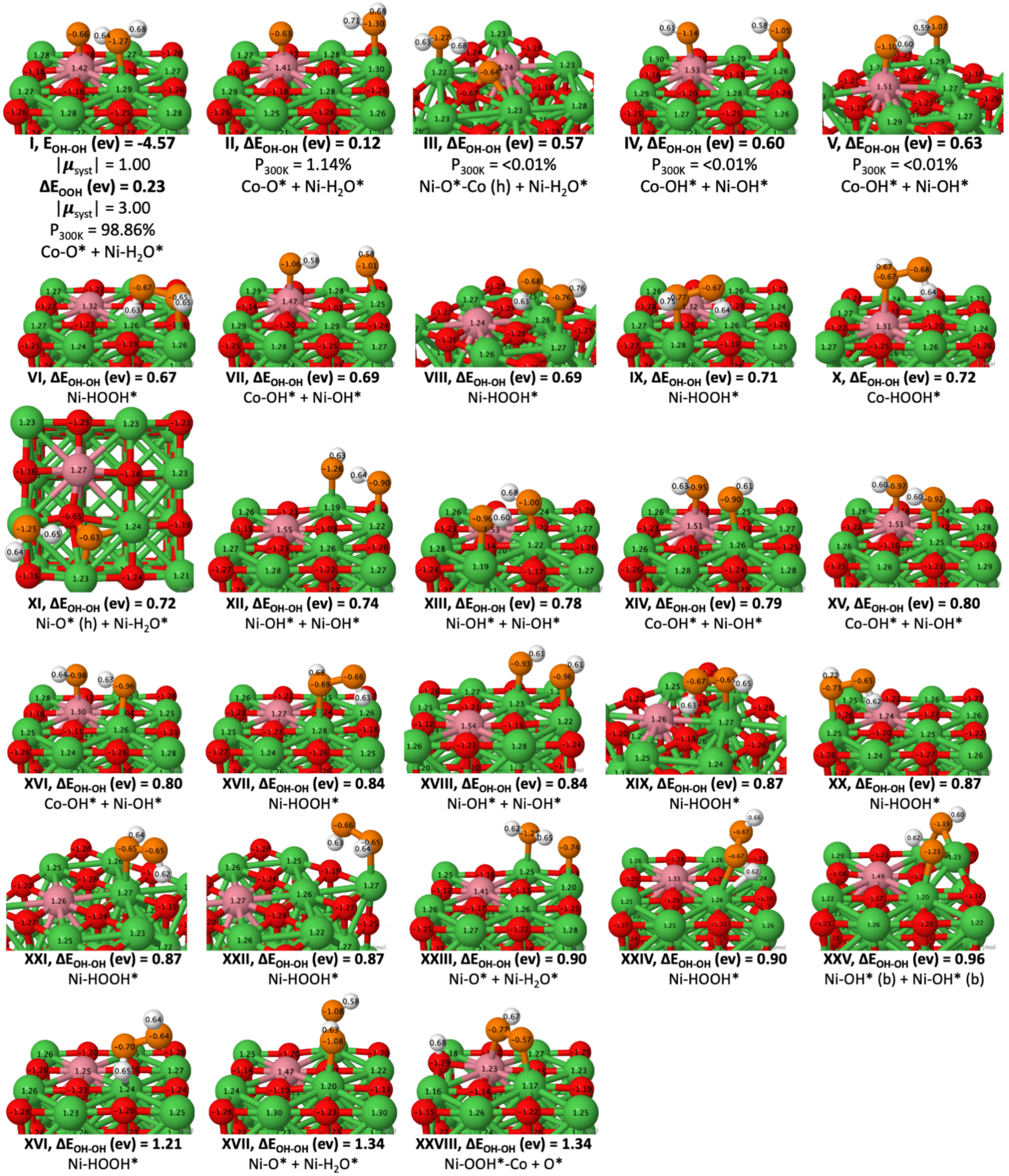

Figure 21. Lowest minima of co-adsorbed OH* + OH* on the rock-salt Co_{sub}-NiO (100) surface with adsorbed energy of the global minimum structure (E_{ads}), relative energies of local minima with respect to the global minimum structure (E_{ads}), and Boltzmann populations at 300 K. Bader charges are shown on atoms. Cobalt atoms are in pink, nickel atoms are in green, oxygen atoms belonging to the Co_{sub}-NiO surface are in red, oxygen atoms belonging to the adsorbed species are in orange, and hydrogen atoms are in white.

Table 14. $OH^* + OH^*$ Adsorption on Co_{sub}-NiO

Isomer	ΔE_{OH+OH} (eV)	Species	$r_{O\text{-}O}$ (Å)	$r_{O\text{-}H}$ (Å)	$r_{surf\text{-}ads}$ (Å)	q_{Co} (e)	q_{Ni} (e)	$q_{O,surf}$ (e)	$q_{OH+OH(ads)}$
I	0.00	Co-O* + Ni-H_2O*	2.68	0.97-1.01	1.64, (Co-O_{ads}) 2.10, (Ni-$O_{ads}H_2$)	1.42	1.21-1.29	-1.27 to -1.15	-0.60
II	0.12	Co-O* + Ni-H_2O*	4.06	0.98	1.62, (Co-O_{ads}) 2.11, (Ni-$O_{ads}H_2$)	1.41	1.21-1.30	-1.27 to -1.16	-0.54
III	0.57	Co-O*-Ni + Ni-H_2O*	2.70	0.98-1.00	2.04, (Co-O_{ads}) 2.06, (Ni-O_{ads}) 2.15, (Ni-$O_{ads}H_2$)	1.24	1.21-1.28	-1.28 to -0.67	-0.59
IV	0.60	Co-OH* + Ni-OH*	4.25	0.98	1.84, (Co-O_{ads}H) 1.94, (Ni-O_{ads}H)	1.53	1.22-1.30	-1.26 to -1.12	-1.00
V	0.63	Co-OH* + Ni-OH*	3.75	0.98	1.85, (Co-O_{ads}H) 1.94, (Ni-O_{ads}H)	1.51	1.21-1.29	-1.26 to -1.09	-0.99
VI	0.67	Ni-HOOH*	1.48	0.98-1.04	2.17, (Ni-O) 1.56, (O_{surf}-H)	1.32	1.22-1.28	-1.29 to -1.19	-0.04
VII	0.69	Co-OH* + Ni-OH*	3.51	0.98	1.88, (Co-OH_{ads}) 1.96, (Ni-OH_{ads})	1.47	1.21-1.29	-1.28 to -1.02	-0.92
VIII	0.69	Ni-HOOH*	1.48	0.98-1.02	2.16, (Ni-O) 1.64, (O_{surf}-H)	1.24	1.22-1.27	-1.28 to -1.20	-0.04
IX	0.71	Ni-HOOH*	1.48	0.98-1.04	2.17, (Ni-O) 1.55, (O_{surf}-H)	1.32	1.22-1.28	-1.29 to -1.19	-0.04
X	0.72	Co-HOOH*	1.48	0.98-1.04	2.21, (Co-O) 1.55, (O_{surf}-H)	1.31	1.23-1.27	-1.28 to -1.20	-0.04
XI	0.72	Ni-O* (h) + Ni-H_2O*	2.69	0.97-1.00	1.99-2.00, (Ni-O_{ads}) 1.49, (O_{surf}-O)	1.27	1.21-1.29	-1.28 to -0.65	-0.59

					2.17, (Ni-$O_{ads}H_2$)				
XII	0.74	Ni-OH*	2.53	0.97-1.07	1.91-1.97, (Ni-O_{ads}H)	1.55	1.19-1.28	-1.27 to -1.04	-0.89
XIII	0.78	Ni-OH*	2.36	0.98	1.99, (Ni-O_{ads}H)	1.53	1.19-1.29	-1.27 to -1.10	-0.67
XIV	0.79	Co-OH* + Ni-OH*	2.31	0.98	1.95, (Co-OH_{ads}) 2.02, (Ni-OH_{ads})	1.51	1.22-1.28	-1.27 to -1.13	-0.62
XV	0.80	Co-OH* + Ni-OH*	2.50	0.98	1.96, (Co-OH_{ads}) 1.98, (Ni-OH_{ads})	1.51	1.22-1.29	-1.27 to -1.12	-0.68
XVI	0.80	Co-OH* + Ni-OH*	2.71	0.98-1.00	1.84, (Co-OH_{ads}) 1.95, (Ni-OH_{ads})	1.30	1.21-1.30	-1.26 to -1.04	-0.68
XVII	0.84	Ni-HOOH*	1.48	0.98-1.04	2.17, (Ni-O) 1.54, (O_{surf}-H)	1.27	1.18-1.28	-1.27 to -1.18	-0.04
XVIII	0.84	Ni-OH*	2.41	0.98	1.98, (Ni-OH_{ads})	1.54	1.20-1.28	-1.26 to -1.12	-0.68
XIX	0.87	Ni-HOOH*	1.48	0.98-1.04	2.17, (Ni-O) 1.54, (O_{surf}-H)	1.26	1.19-1.27	-1.27 to -1.19	-0.04
XX	0.87	Ni-HOOH*	1.48	0.98-1.04	2.17, (Ni-O) 1.53, (O_{surf}-H)	1.24	1.18-1.26	-1.27 to -1.20	-0.04
XXI	0.87	Ni-HOOH*	1.48	0.98-1.04	2.17, (Ni-O) 1.55, (O_{surf}-H)	1.26	1.22-1.27	-1.29 to -1.19	-0.04
XXII	0.87	Ni-HOOH*	1.48	0.98-1.04	2.17, (Ni-O) 1.55, (O_{surf}-H)	1.27	1.19-1.27	-1.28 to -1.19	-0.04
XXIII	0.90	Ni-O* + Ni-H_2O*	2.52	0.98-1.07	1.88, (Ni-O_{ads}) 2.05, (Ni-$O_{ads}H_2$)	1.41	1.20-1.28	-1.27 to -1.12	-0.73
XXIV	0.96	Ni-HOOH*	1.48	0.98-1.04	2.37, (Ni-O)	1.33	1.20-1.27	-1.29 to -1.19	-0.06
XXV	1.05	Ni-OH*	3.12	0.97	1.97-1.98, (Ni-OH_{ads})	1.49	1.19-1.29	-1.27 to -1.05	-1.20
XXVI	1.14	Ni-HOOH*	1.48	0.98-1.04	2.37, (Ni-O) 1.56, (O_{surf}-H)	1.25	1.23-1.27	-1.28 to -1.20	-0.05

XXVII	1.21	Ni-OH*	3.78	0.97-0.98	1.96, (Ni-OH_{ads})	1.47	1.20-1.30	-1.28 to -1.07	-0.96
XXVIII	1.34	Co-HOO*-Ni + H*	1.51	0.98	2.21, (Co-O) 2.00, (Ni-O) 0.98, (O_{surf}-H)	1.23	1.16-1.27	-1.29 to -1.14	0.01

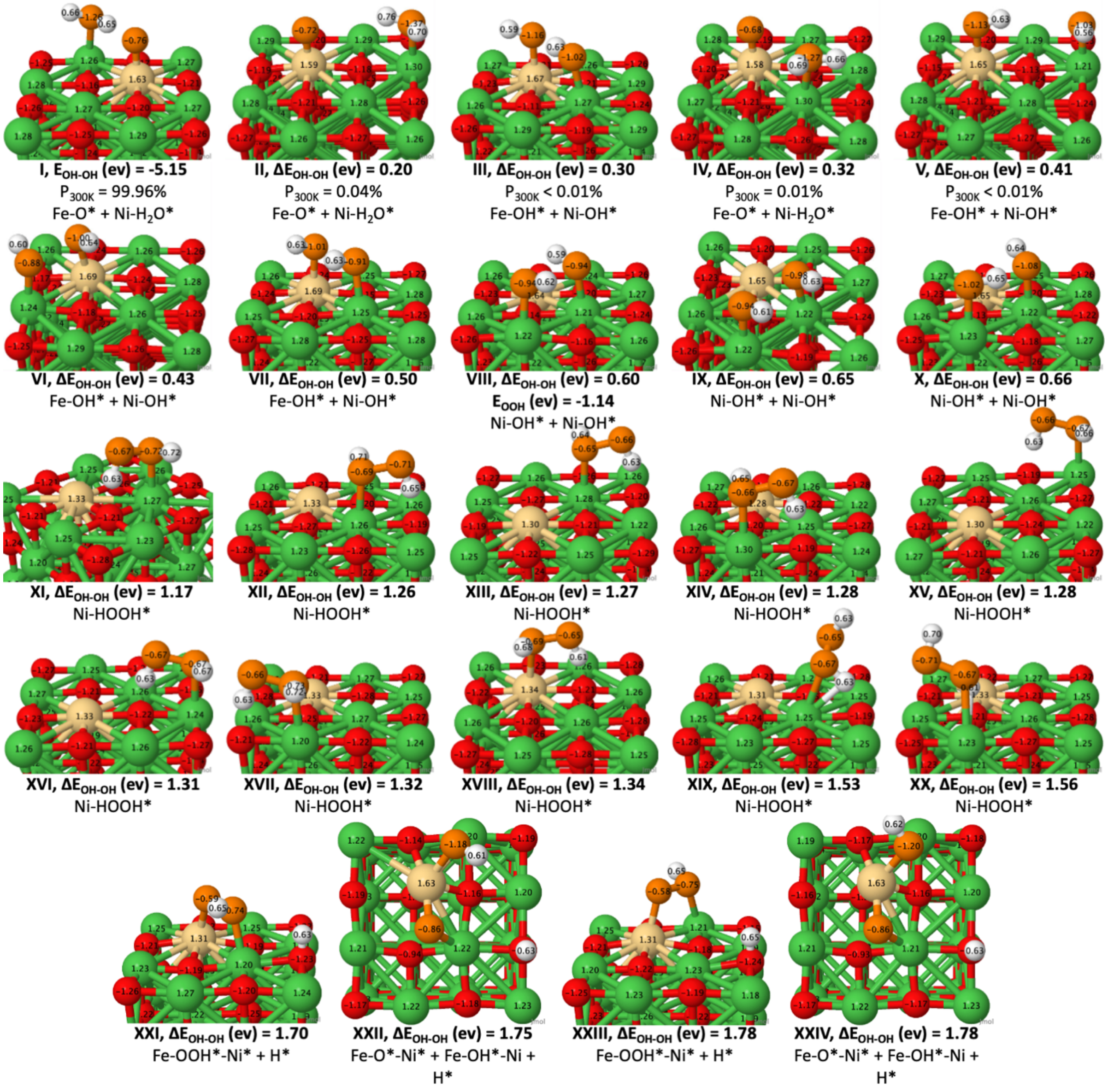

Figure 22. Lowest minima of co-adsorbed OH* + OH* on the rock-salt Fe_{sub}-NiO (100) surface with adsorbed energy of the global minimum structure (E_{ads}), relative energies of local minima with respect to the global minimum structure (E_{ads}), and Boltzmann populations at 300 K. Bader charges are shown on atoms. Iron atoms are in yellow, nickel atoms are in green, oxygen atoms

belonging to the Fe_{sub}-NiO surface are in red, oxygen atoms belonging to the adsorbed species are in orange, and hydrogen atoms are in white.

Table 15. OH* + OH* Adsorption on Fe_{sub}-NiO

Isomer	ΔE_{OH+OH} (eV)	Species	r_{O-O} (Å)	r_{O-H} (Å)	$r_{surf-ads}$ (Å)	q_{Fe} (e)	q_{Ni} (e)	$q_{O,surf}$ (e)	$q_{OH+OH(ads)}$
I	0.00	Fe-O* + Ni-H_2O*	2.63	0.97-1.02	1.69, (Fe-O_{ads}) 2.09, (Ni-$O_{ads}H_2$)	1.63	1.21-1.29	-1.28 to -1.16	-0.70
II	0.20	Fe-O* + Ni-H_2O*	4.09	0.97-0.98	1.66, (Fe-O_{ads}) 2.10, (Ni-$O_{ads}H_2$)	1.59	1.22-1.30	-1.27 to -1.18	-0.63
III	0.30	Fe-OH* + Ni-OH*	2.76	0.97-1.00	1.93, (Fe-O_{ads}H) 1.94, (Ni-O_{ads}H)	1.67	1.21-1.29	-1.26 to -1.11	-0.96
IV	0.32	Fe-O* + Ni-H_2O*	3.29	0.97-0.98	1.66, (Fe-O_{ads}) 2.12, (Ni-$O_{ads}H_2$)	1.58	1.22-1.30	-1.28 to -1.19	-0.60
V	0.41	Fe-OH* + Ni-OH*	4.03	0.97-0.98	1.89, (Fe-O_{ads}H) 1.92, (Ni-O_{ads}H)	1.65	1.21-1.29	-1.27 to -1.13	-0.97
VI	0.43	Fe-OH* + Ni-OH*	2.36	0.98	2.00, (Fe-O_{ads}H) 2.00, (Ni-O_{ads}H)	1.69	1.22-1.29	-1.27 to -1.17	-0.64
VII	0.50	Fe-OH* + Ni-OH*	2.40	0.98	1.99, (Fe-O_{ads}H) 2.01, (Ni-O_{ads}H)	1.69	1.22-1.28	-1.27 to -1.15	-0.66
VIII	0.60	Ni-OH*	2.36	0.97-0.98	1.98-2.00, (Ni-O_{ads}H)	1.64	1.21-1.28	-1.27 to -1.14	-0.68
IX	0.65	Ni-OH*	2.40	0.98	1.98-2.00, (Ni-O_{ads}H)	1.65	1.22-1.28	-1.26 to -1.13	-0.68
X	0.66	Ni-OH*	2.60	0.97-1.00	1.97-1.98, (Ni-O_{ads}H)	1.65	1.21-1.28	-1.27 to -1.13	-0.82
XI	1.17	Ni-HOOH*	1.48	0.98-1.04	2.17, (Ni-O) 1.54, (O_{surf}-H)	1.33	1.20-1.27	-1.28 to -1.21	-0.05
XII	1.26	Ni-HOOH*	1.47	0.98-1.04	2.18, (Ni-O) 1.53, (O_{surf}-H)	1.33	1.19-1.26	-1.28 to -1.19	-0.04

XIII	1.27	Ni-HOOH*	1.48	0.98-1.04	2.17, (Ni-O) 1.55, (O_{surf}-H)	1.30	1.19-1.28	-1.29 to -1.19	-0.04
XIV	1.28	Ni-HOOH*	1.48	0.98-1.04	2.17, (Ni-O) 1.55, (O_{surf}-H)	1.28	1.21-1.30	-1.28 to -1.19	-0.04
XV	1.28	Ni-HOOH*	1.48	0.98-1.04	2.18, (Ni-O) 1.55, (O_{surf}-H)	1.30	1.19-1.27	-1.27 to -1.19	-0.04
XVI	1.31	Ni-HOOH*	1.48	0.98-1.04	2.19, (Ni-O) 1.55, (O_{surf}-H)	1.33	1.19-1.28	-1.28 to -1.21	-0.05
XVII	1.32	Ni-HOOH*	1.48	0.98-1.04	2.21, (Ni-O) 1.60, (O_{surf}-H)	1.33	1.20-1.28	-1.28 to -1.21	-0.04
XVIII	1.34	Fe-HOOH*	1.48	0.98-1.04	2.27, (Fe-O) 1.57, (O_{surf}-H)	1.34	1.21-1.26	-1.28 to -1.20	-0.05
XIX	1.53	Ni-HOOH*	1.48	0.98-1.05	2.34, (Ni-O) 1.53, (O_{surf}-H)	1.31	1.21-1.26	-1.28 to -1.19	-0.06
XX	1.56	Ni-HOOH*	1.48	0.98-1.04	2.46, (Ni-O) 1.54, (O_{surf}-H)	1.33	1.19-1.27	-1.28 to -1.21	-0.06
XXI	1.70	Fe-OOH*-Ni + H*	1.53	0.98	2.02, (Fe-OOH_{ads}) 2.13, (Ni-OOH_{ads}) 0.98, (O_{surf}-H)	1.31	1.19-1.27	-1.28 to -1.19	-0.05
XXII	1.75	Fe-O*-Ni + Fe-OH*-Ni + H*	3.12	0.97	1.84, (Fe-O_{ads}) 2.18, (Ni-O_{ads}) 1.98, (Fe-O_{ads}H) 2.12, (Ni-O_{ads}H) 0.98, (O_{surf}-H)	1.63	1.18-1.28	-1.28 to -0.94	-0.80
XXIII	1.78	Fe-OOH*-Ni + H*	1.53	0.98	2.15, (Fe-OOH_{ads}) 0.98, (O_{surf}-H)	1.31	1.18-1.27	-1.28 to -1.15	-0.04
XXIV	1.78	Fe-O*-Ni + Fe-OH*-Ni + H*	3.12	0.97	1.84, (Fe-O_{ads}) 2.18, (Ni-O_{ads}) 1.98, (Fe-O_{ads}H) 2.12, (Ni-O_{ads}H) 0.98, (O_{surf}-H)	1.63	1.18-1.28	-1.28 to -0.93	-0.80

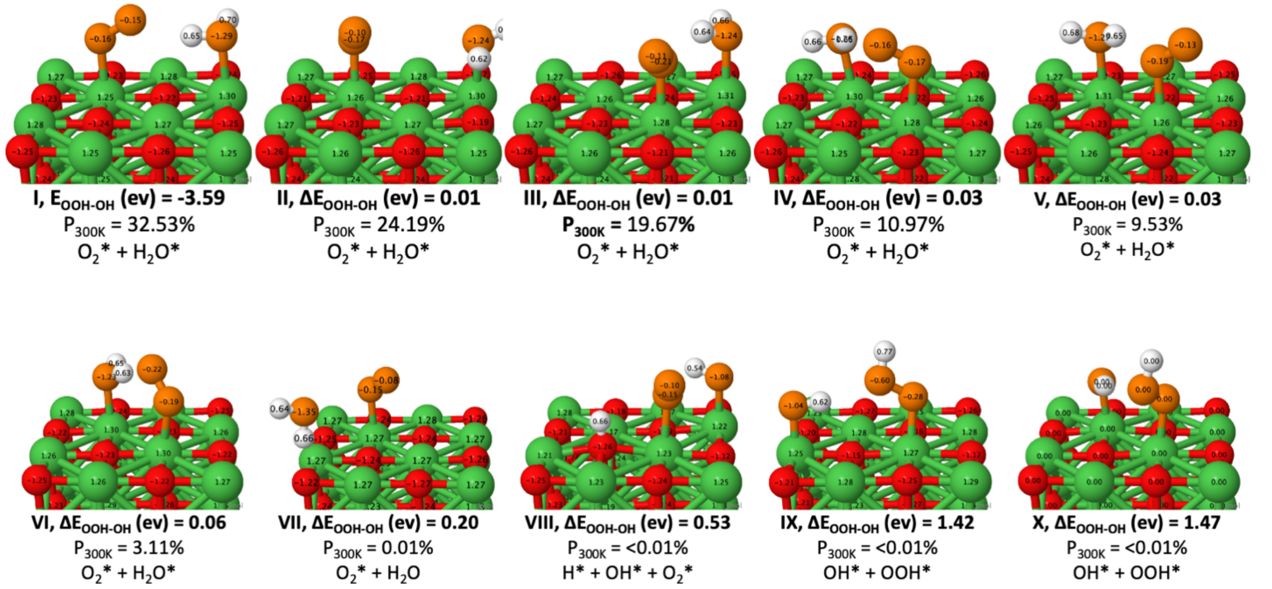

Figure 17. Lowest minima of co-adsorbed geometries resulting from OOH* + OH* on the rock-salt NiO (100) surface with adsorbed energy of the global minimum structure (E_{ads}), relative energies of local minima with respect to the global minimum structure (E_{ads}), and Boltzmann populations at 300 K. Bader charges are shown on atoms. Nickel atoms are in green, oxygen atoms belonging to the NiO surface are in red, oxygen atoms belonging to the adsorbed species are in orange, and hydrogen atoms are in white.

Table 16. OOH* + OH* Adsorption on NiO

Isomer	ΔE_{OH} (eV)	Species	r_{O-O} (Å)	r_{O-H} (Å)	$r_{surf-ads}$ (Å)	q_{Ni} (e)	$q_{O,surf}$ (e)	$q_{OOH+OH(ads)}$
I	0.00	OO* + H_2O*	1.23	0.98-0.99	2.13, (Ni-OO_{ads})	1.20-1.27	-1.27 to -1.20	-0.01
II	0.01	OO* + H_2O*	1.23	0.97-0.99	2.12, (Ni-$O_{ads}H_2$)	1.21-1.27	-1.27 to -1.21	-0.05
III	0.01	OO* + H_2O*	1.26	0.98	2.13, (Ni-OO_{ads}) 2.12, (Ni-$O_{ads}H_2$)	1.20-1.30	-1.27 to -1.19	-0.18
IV	0.03	OO* + H_2O*	1.26	0.98-0.99	2.15, (Ni-OO_{ads}) 2.16, (Ni-$O_{ads}H_2$)	1.20-1.29	-1.27 to -1.18	-0.17
V	0.03	OO* + H_2O*	1.26	0.98	2.15, (Ni-OO_{ads}) 2.14, (Ni-$O_{ads}H_2$)	1.20-1.28	-1.27 to -1.20	-0.16
VI	0.06	OO* + H_2O*	1.26	0.98	2.14, (Ni-OO_{ads}) 2.14, (Ni-$O_{ads}H_2$)	1.21-1.31	-1.27 to -1.18	-0.19
VII	0.20	OO* + H_2O	1.26	0.97-0.98	2.13, (Ni-OO_{ads}) 2.14, (Ni-$O_{ads}H_2$)	1.21-1.31	-1.27 to -1.19	-0.21

VIII	0.53	H* + OH* + OO*	1.25	0.98-0.99	2.16, (Ni-OO_{ads}) 2.17, (Ni-OH_{ads}) 2.17, (O_{surf} -H)	1.20-1.29	-1.27 to -1.18	-0.13
IX	1.42	OH* + OOH*	1.37	0.98-0.99	1.85, (Ni-OH_{ads}) 2.02, (Ni-OOH_{ads})	1.20-1.29	-1.27 to -1.17	-0.22
X	1.47	OH* + OOH*	1.37	0.97-0.99	1.94, (Ni-OH_{ads}) 2.05, (Ni-OOH_{ads})	1.20-1.29	-1.27 to -1.19	-0.15

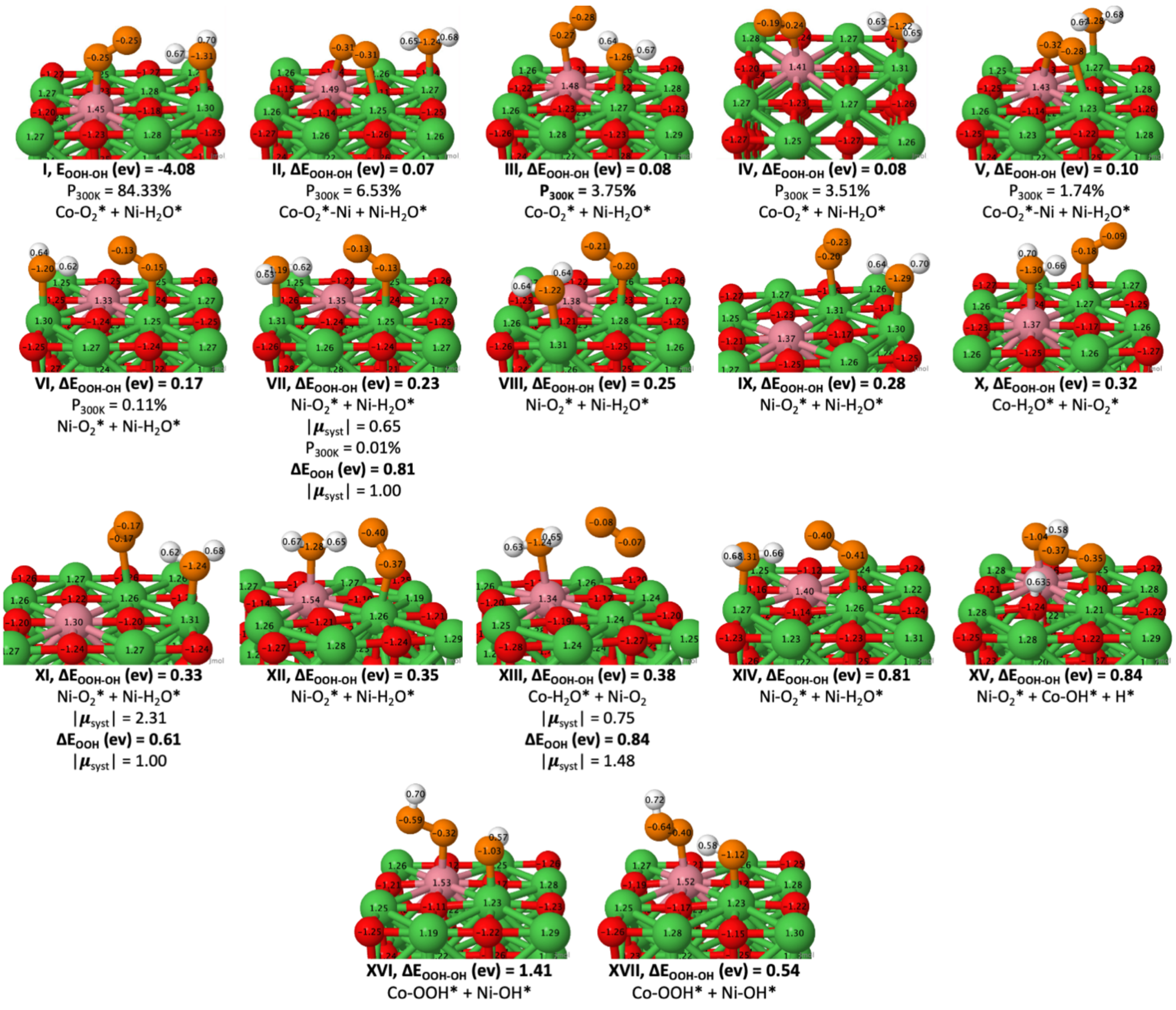

Figure 23. Lowest minima of co-adsorbed OOH* + OH* on the rock-salt Co_{sub}-NiO (100) surface with adsorbed energy of the global minimum structure (E_{ads}), relative energies of local minima with respect to the global minimum structure (E_{ads}), and Boltzmann populations at 300 K. Bader charges are shown on atoms. Cobalt atoms are in pink, nickel atoms are in green, oxygen atoms

belonging to the Co_{sub}-NiO surface are in red, oxygen atoms belonging to the adsorbed species are in orange, and hydrogen atoms are in white.

Table 17. OOH* + OH* Adsorption on Co_{sub}-NiO

Isomer	ΔE_{OOH+OH} (eV)	Species	r_{O-O} (Å)	r_{O-H} (Å)	$r_{surf-ads}$ (Å)	q_{Co} (e)	q_{Ni} (e)	$q_{O,surf}$ (e)	$q_{OOH+OH(ads)}$
I	0.00	O_2* + H_2O*	1.28	0.97-0.98	2.13, (Co-OO_{ads}) 2.11, (Ni-$O_{ads}H_2$)	1.39	1.20-1.26	-1.27 to -1.17	-0.29
II	0.07	O_2* + H_2O*	1.27	0.97-0.99	2.03, (Co-OO_{ads}) 2.11, (Ni-$O_{ads}H_2$)	1.35	1.20-1.27	-1.27 to -1.15	-0.23
III	0.08	O_2* + H_2O*	1.29	0.97-0.98	2.13, (Co-OO_{ads}) 2.13, (Ni-$O_{ads}H_2$)	1.38	1.21-1.27	-1.27 to -1.16	-0.36
IV	0.08	O_2* + H_2O*	1.25	0.98-0.99	2.32, (Ni-OO_{ads}) 2.16, (Ni-$O_{ads}H_2$)	1.27	1.20-1.28	-1.27 to -1.21	-0.10
V	0.10	O_2* + H_2O*	1.26	0.98	2.28, (Ni-OO_{ads}) 2.14, (Ni-$O_{ads}H_2$)	1.29	1.20-1.29	-1.27 to -1.20	-0.15
VI	0.17	O_2 + H_2O*	1.24	0.97-0.99	2.17, (Ni-$O_{ads}H_2$)	1.22	1.20-1.26	-1.27 to -1.17	-0.01
VII	0.23	O_2* + H_2O*	1.25	0.98	2.30, (Ni-OO_{ads}) 2.15, (Ni-$O_{ads}H_2$)	1.30	1.20-1.28	-1.27 to -1.21	-0.11
VIII	0.25	O_2* + H_2O*	1.28	0.97-0.98	2.30, (Ni-OO_{ads}) 2.15, (Ni-$O_{ads}H_2$)	1.39	1.21-1.28	-1.27 to -1.17	-0.30
IX	0.28	O_2* + H_2O*	1.32	0.97-0.99	2.24, (Ni-OO_{ads}) 2.11, (Ni-$O_{ads}H_2$)	1.44	1.20-1.30	-1.27 to -1.13	-0.55
X	0.32	O_2 + H_2O*	1.23	0.98-0.99	2.17, (Ni-$O_{ads}H$)	1.29	1.20-1.27	-1.27 to -1.19	0.02

XI	0.33	O_2 + H_2O*	1.23	0.98-0.99	2.18, (Ni-$O_{ads}H$)	1.28	1.19-1.29	-1.27 to -1.21	-0.01
XII	0.35	O_2* + H_2O*	1.31	0.97-0.99	2.30, (Ni-OO_{ads}) 2.13, (Ni-$O_{ads}H_2$)	1.43	1.19-1.31	-1.27 to -1.16	-0.50
XIII	0.38	O_2 + H_2O*	1.24	0.98	2.24, (Co-$O_{ads}H$)	1.27	1.19-1.27	-1.27 to -1.17	-0.06
XIV	0.81	O_2* + H_2O*	1.26	0.97-0.98	2.32, (Ni-OO_{ads}) 2.20, (Co-$O_{ads}H_2$)	1.36	1.20-1.28	-1.27 to -1.18	-0.19
XV	0.89	O_2* + H_2O*	1.28	0.97-0.99	2.13, (Ni-OO_{ads}) 2.12, (Ni-$O_{ads}H_2$)	1.30	1.21-1.29	-1.27 to -1.16	-0.28
XVI	1.41	O_2* + H_2O*	1.27	0.97-0.98	2.14, (Ni-OO_{ads}) 2.14, (Ni-$O_{ads}H_2$)	1.32	1.20-1.26	-1.27 to -1.20	-0.23
XVII	1.54	O_2* + H_2O*	1.27	0.98-0.99	2.17, (Ni-OO_{ads}) 2.17, (Co-$O_{ads}H_2$)	1.36	1.20-1.28	-1.27 to -1.16	-0.28

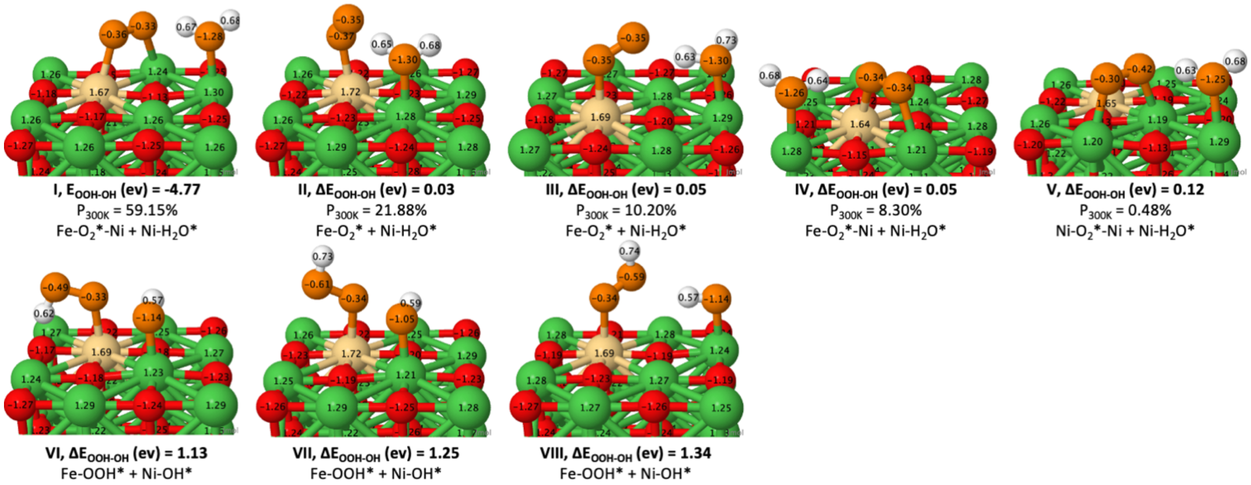

Figure 24. Lowest minima of co-adsorbed OOH* + OH* on the rock-salt Fe_{sub}-NiO (100) surface with adsorbed energy of the global minimum structure (E_{ads}), relative energies of local minima with respect to the global minimum structure (E_{ads}), and Boltzmann populations at 300 K. Bader

charges are shown on atoms. Iron atoms are in yellow, nickel atoms are in green, oxygen atoms belonging to the Fe_{sub}-NiO surface are in red, oxygen atoms belonging to the adsorbed species are in orange, and hydrogen atoms are in white.

Table 18. OOH* + OH* Adsorption on Fe_{sub}-NiO

Isomer	ΔE_{OOH+OH} (eV)	Species	r_{O-O} (Å)	r_{O-H} (Å)	$r_{surf-ads}$ (Å)	q_{Fe} (e)	q_{Ni} (e)	$q_{O,surf}$ (e)	$q_{OOH+OH(ads)}$
I	0.00	Fe-O_2*-Ni + Ni-H_2O*	1.34	0.98-0.99	2.16, (Fe-OO_{ads}) 2.16, (Ni-OO_{ads}) 2.11, (Ni-$O_{ads}H_2$)	1.67	1.18-1.30	-1.28 to -1.13	-0.62
II	0.03	Fe-O_2* + Ni-H_2O*	1.34	0.97-1.02	2.07, (Fe-OO_{ads}) 2.06, (Ni-$O_{ads}H_2$)	1.72	1.22-1.29	-1.28 to -1.22	-0.69
III	0.05	Fe-O_2* + Ni-H_2O*	1.33	0.97-1.00	2.06, (Fe-OO_{ads}) 2.09, (Ni-$O_{ads}H_2$)	1.69	1.22-1.29	-1.28 to -1.18	-0.64
IV	0.05	Fe-O_2* + Ni-H_2O*	1.34	0.98	2.17, (Fe-OO_{ads}) 2.12, (Ni-$O_{ads}H_2$)	1.64	1.21-1.28	-1.28 to -1.14	-0.62
V	0.12	Ni-O_2*-Ni + Ni-H_2O*	1.35	0.98-0.99	2.11-2.16 (Ni-OO_{ads}) 2.11, (Ni-$O_{ads}H_2$)	1.65	1.19-1.29	-1.28 to -1.13	-0.65
VI	1.13	Fe-OOH* + Ni-OH*	1.37	0.97-1.08	2.11, (Fe-OOH_{ads}) 1.96, (Ni-OH_{ads})	1.69	1.20-1.29	-1.27 to -1.17	-0.78
VII	1.25	Fe-OOH* + Ni-OH*	1.40	0.97-0.98	2.11, (Fe-OOH_{ads}) 1.97, (Ni-OH_{ads})	1.72	1.21-1.29	-1.26 to -1.19	-0.67
VIII	1.34	Fe-OOH* + Ni-OH*	1.39	0.98	2.11, (Fe-OOH_{ads}) 1.97, (Ni-OH_{ads})	1.69	1.22-1.28	-1.27 to -1.19	-0.76

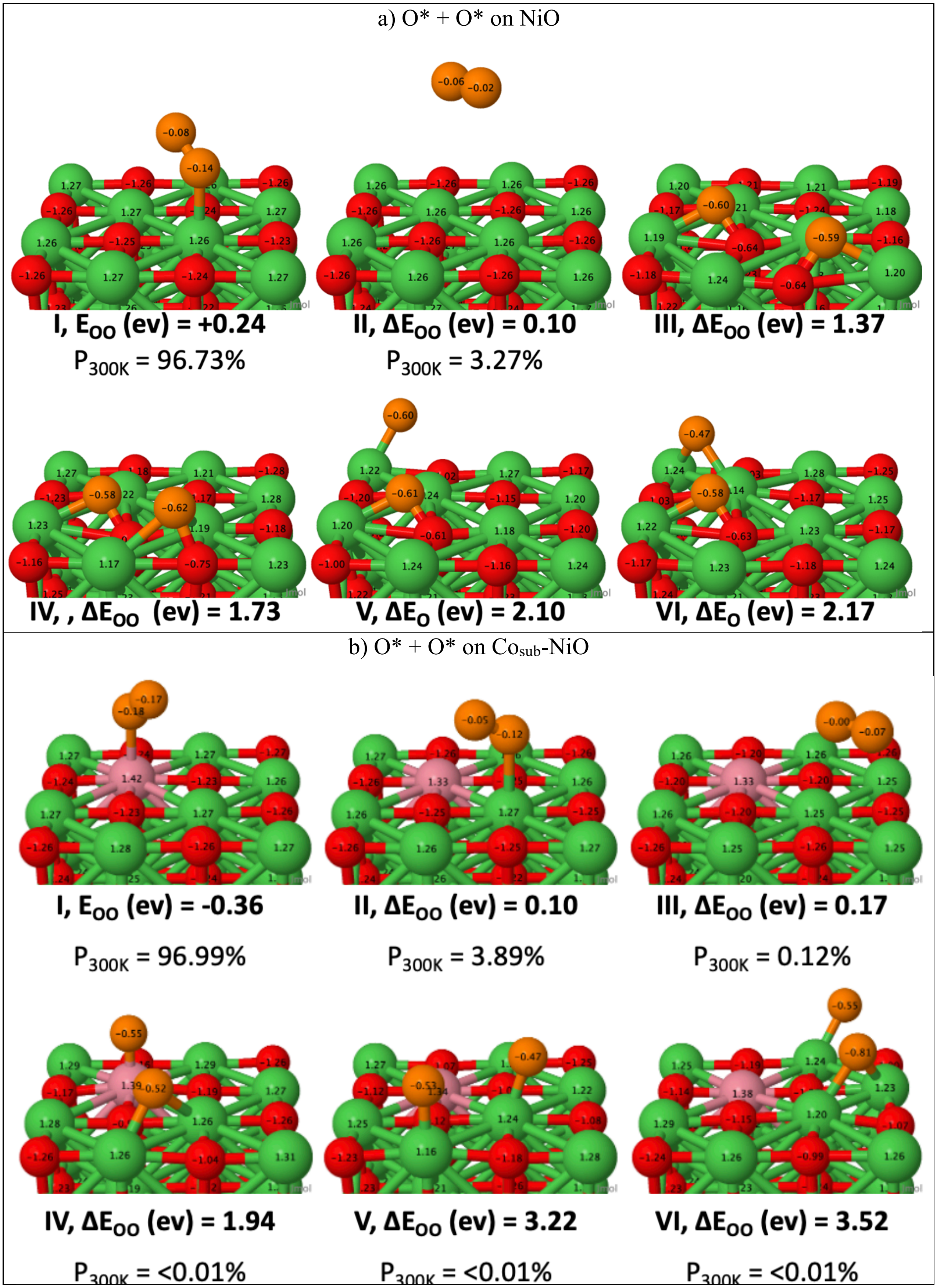
a) O* + O* on NiO
I, E_OO (ev) = +0.24
P_300K = 96.73%
II, ΔE_OO (ev) = 0.10
P_300K = 3.27%
III, ΔE_OO (ev) = 1.37
IV, , ΔE_OO (ev) = 1.73
V, ΔE_O (ev) = 2.10
VI, ΔE_O (ev) = 2.17
b) O* + O* on Co_sub-NiO
I, E_OO (ev) = -0.36
P_300K = 96.99%
II, ΔE_OO (ev) = 0.10
P_300K = 3.89%
III, ΔE_OO (ev) = 0.17
P_300K = 0.12%
IV, ΔE_OO (ev) = 1.94
P_300K = <0.01%
V, ΔE_OO (ev) = 3.22
P_300K = <0.01%
VI, ΔE_OO (ev) = 3.52
P_300K = <0.01%

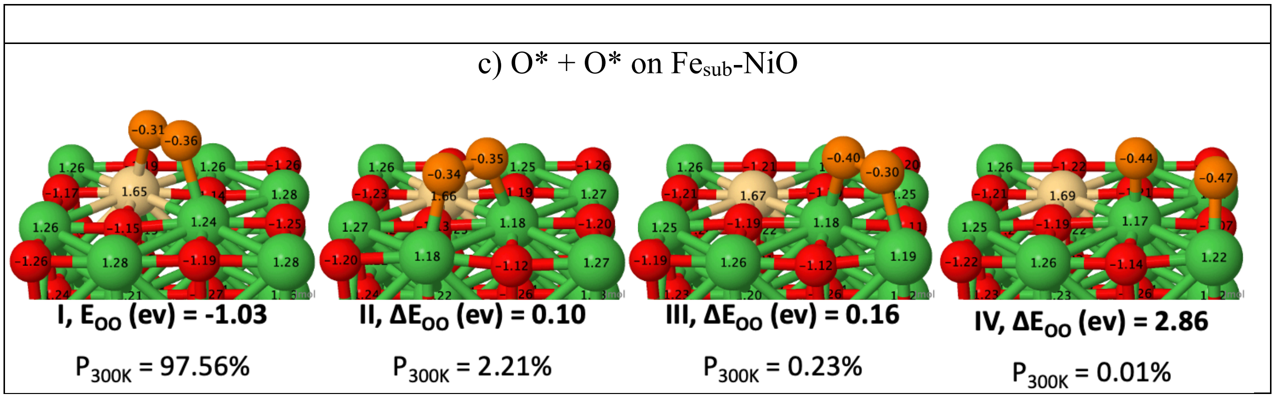

Figure 25. Lowest minima of co-adsorbed O* + O* on the rock-salt NiO, Co$_{sub}$-NiO, Fe$_{sub}$-NiO (100) surface with adsorbed energy of the global minimum structure (E$_{ads}$), relative energies of local minima with respect to the global minimum structure (E$_{ads}$), and Boltzmann populations at 300 K. Bader charges are shown on atoms. Iron atoms are in yellow, nickel atoms are in green, oxygen atoms belonging to the Fe$_{sub}$-NiO surface are in red, oxygen atoms belonging to the adsorbed species are in orange, and hydrogen atoms are in white.

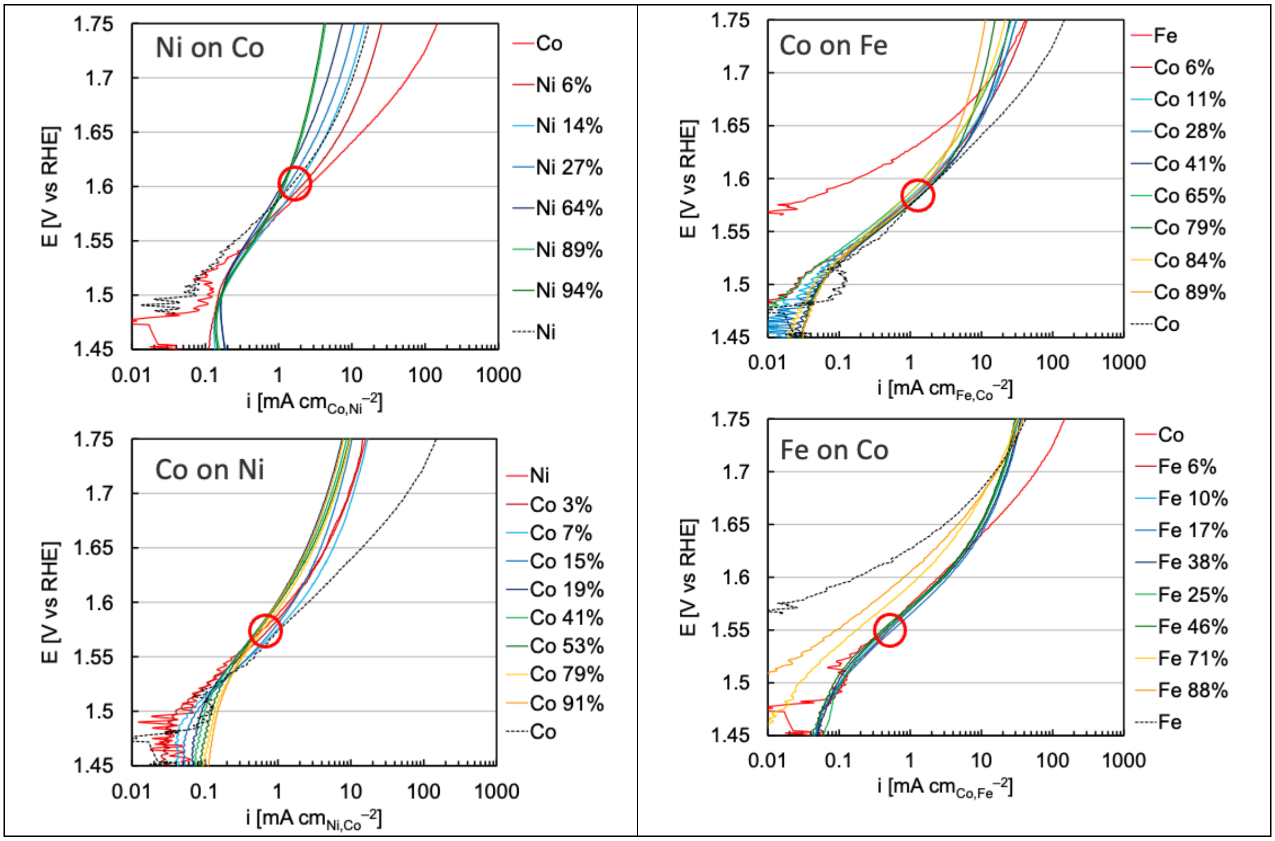

Figure 26. Electrochemistry data for Ni/Co, Co/Fe electrodeposited combinations.

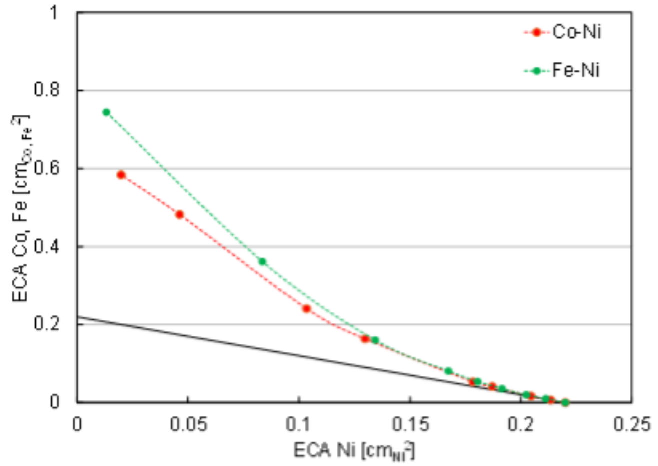

Figure 27. Electrochemical surface areas (ECAs) of cobalt (y-axis), iron (y-axis), and nickel (x-axis) following electrochemical deposition experiments for cobalt deposited onto nickel (red) and iron deposited onto nickel (green). ECA measurements were determined by redox transitions, using polycrystalline electrodes for the Coulombic charge conversion and assuming a roughness factor of 1.1. The black line denotes a linear replacement of nickel ECA with cobalt/iron and assumes a monolayered deposition, demonstrating heterogeneity in the electrochemical deposition process.

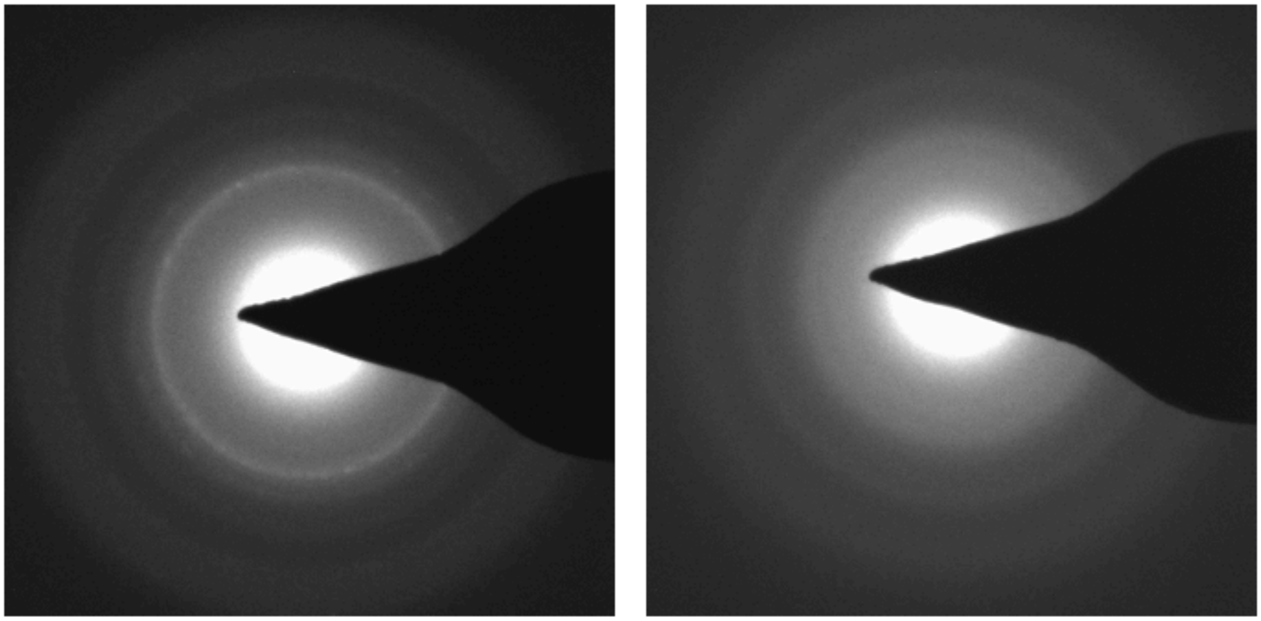

Figure 28. TED patterns of high performing Ni-Fe synthesized nanoparticles.

a)

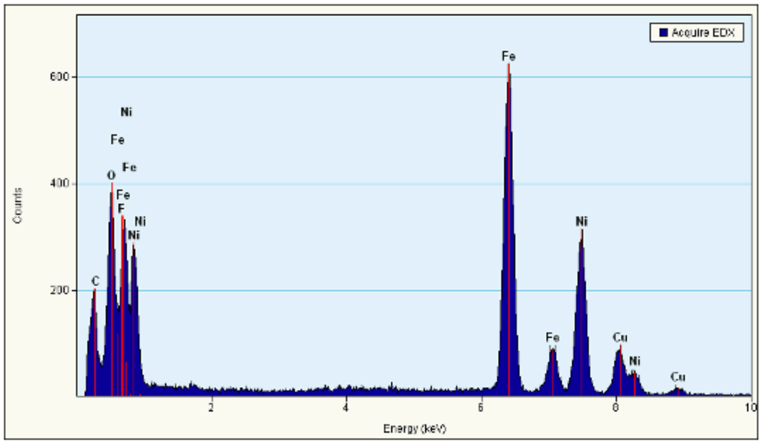

Element	Weight %	Atomic %	Uncert. %	Correction	k-Factor
-------	-------- -------- ---------		----------	--------	
O(K)	27.65	57.57	0.34	0.49	2.008
Fe(K)	47.73	28.46	0.32	0.99	1.359
Ni(K)	24.60	13.96	0.26	0.99	1.456

b)

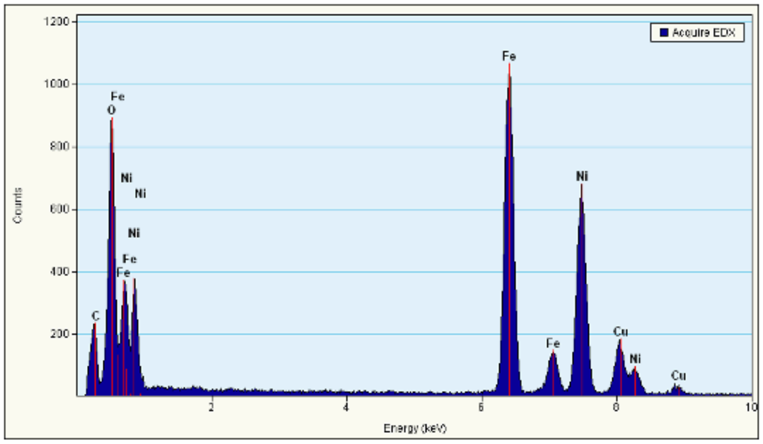

Element	Weight %	Atomic %	Uncert. %	Correction	k-Factor
-------	-------- -------- ---------		----------	--------	
O(K)	29.62	59.99	0.25	0.49	2.008
Fe(K)	40.71	23.62	0.21	0.99	1.359
Ni(K)	29.66	16.37	0.19	0.99	1.456

Figure 29. EDS of high performing Ni-Fe synthesized nanoparticles.

(1) Freakley, S. J.; Ruiz-Esquius, J.; Morgan, D. J. The X-ray photoelectron spectra of Ir, IrO2 and IrCl3 revisited. *Surf. Interface Anal.* **2017**, *49* (8), 794-799.

(2) Biesinger, M. C.; Payne, B. P.; Grosvenor, A. P.; Lau, L. W.; Gerson, A. R.; Smart, R. S. C. Resolving surface chemical states in XPS analysis of first row transition metals, oxides and hydroxides: Cr, Mn, Fe, Co and Ni. *Appl. Surf. Sci.* **2011**, *257* (7), 2717-2730.
(3) Chase, M.; Davies, C.; Downey, J.; Frurip, D. J. Phys. Chem. Ref. Data. *JANAF Thermochemical Tables* **1998**, *4*.
